# Bearing capacity attenuation and energy response of end-anchored bodies under dynamic disturbance with different frequencies

**DOI:** 10.1371/journal.pone.0354419

**Published:** 2026-10-23

**Authors:** Xiaolong Zhen, Wen Wang, Xiaowei Lu, Guang Zhao, Ning Li, Chuanjiu Zhang, Shuren Wang

**Affiliations:** 1 School of Energy Science and Engineering, Henan Polytechnic University, Jiaozuo, Henan, China; 2 Collaborative Innovation Center of Coal Work Safety and Clean High-Efficiency Utilization, Jiaozuo, Henan, China; 3 Zhengzhou Institute for Advanced Research of Henan Polytechnic University, Zhengzhou, China; 4 School of Civil Engineering, Luoyang Institute of Science and Technology, Luoyang, China; 5 Shendong Coal Group of China Energy Co., Ltd., Shenmu, Shanxi, China; 6 School of Civil Engineering, Henan Polytechnic University, Jiaozuo, China; Hohai University, CHINA

## Abstract

Dynamic disturbances significantly impact the stability of end-anchored systems, yet their bearing capacity attenuation and energy response evolution remain poorly understood. This study investigated sandstone end-anchored specimens with anchorage lengths of 300 mm and 600 mm. By conducting comparative static pull-out tests and pull-out tests following sinusoidal cyclic disturbances via a custom-designed apparatus, coupled with Particle Flow Code 2D (PFC^2D^) numerical simulations, the capacity attenuation, energy dissipation, and internal damage evolution were analyzed. Results reveal a negative correlation between bearing capacity and disturbance frequency. Low-frequency (10 Hz) disturbances cause severe damage, triggering extensive interfacial debonding, whereas 50–200 Hz disturbances induce highly localized damage. The failure process involves elastic, slip, debonding-slip, and complete detachment stages, governed by a tensile-shear mode. This is characterized by axial debonding along the cable-anchoring agent interface and radial or oblique tensile micro-cracking within the anchoring agent. Low-frequency energy propagates efficiently over long distances and continuously accumulates, whereas high-frequency energy dissipates rapidly. Furthermore, extending the anchorage length to 600 mm effectively enhances system rigidity and dynamic resistance. Numerical simulations confirm that lower frequencies accelerate tensile crack development, exacerbating internal damage and capacity degradation.

## 1. Introduction

As deep coal mining in China continues to expand, underground working faces are persistently subjected to multi-source dynamic loads including mining-induced blasting vibration and formation microseisms [1]. These dynamic loads originate from three primary sources: mining-induced blasting and excavation, rock mass movements such as overburden fracturing and coal pillar instability [2], and geological activations like fault dislocations and microseisms. Consequently, roadway anchor cable systems are continuously exposed to these sustained and complex dynamic disturbances. In some mines, underground anchor cables undergo more than 20 disturbance events per day on average, with cumulative disturbances exceeding tens of thousands within two years [3] Frequent cyclic dynamic disturbances markedly accelerate the fatigue damage evolution of anchor cables [4] resulting in issues such as debonding at the anchorage interface, fracture of the free segment, and loosening at the tray, as illustrated in Fig 1. Against this background, a systematic study on the mechanical response characteristics and fatigue damage evolution laws of anchor cables under frequent dynamic disturbances, and the clarification of the intrinsic correlation between dynamic disturbances and anchor cable fatigue failure, can provide theoretical support for the optimal design of anchor cable support systems in deep roadways [5]. Ultimately, this research provides compelling engineering implications for optimizing anchorage specifications and deploying frequency-selective anti-impact support components, thereby enhancing the long-term reliability of support systems in rockburst-prone deep roadways [6].

**Fig 1:**
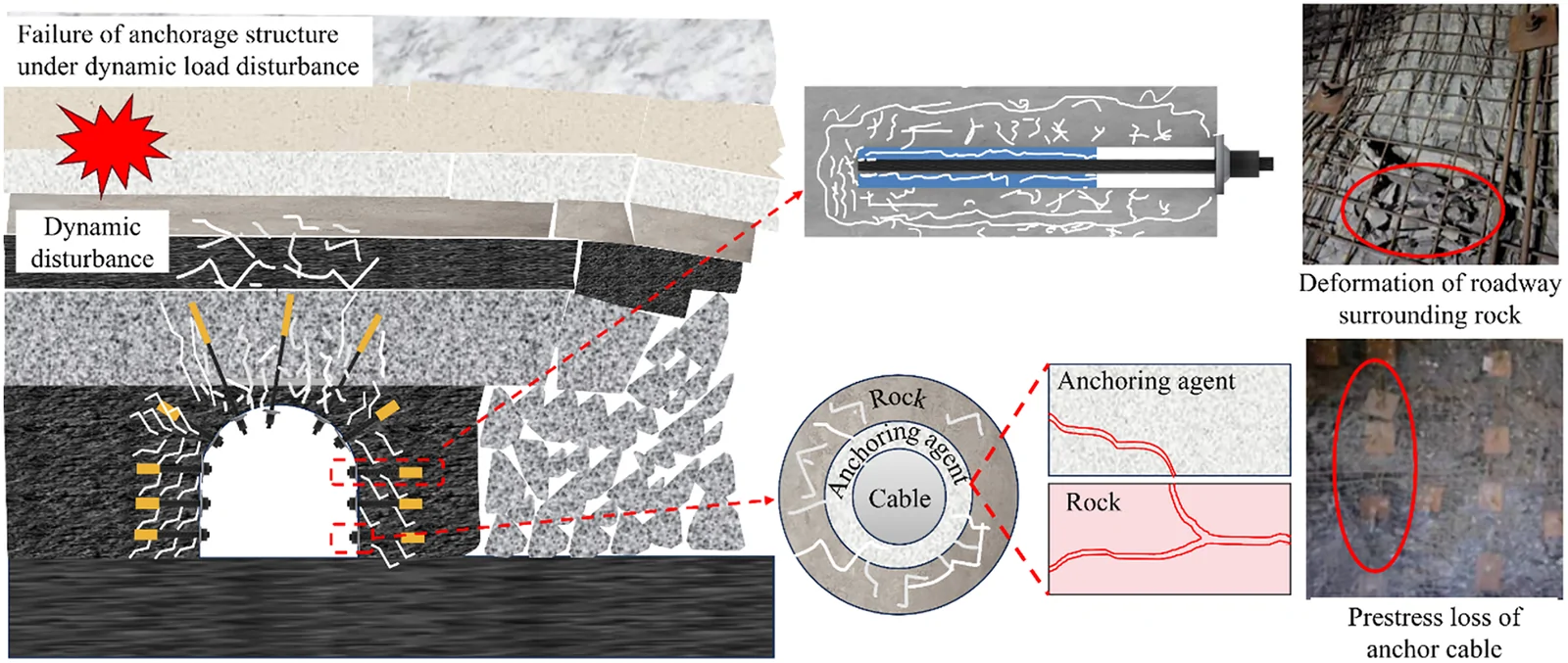
Schematic of anchor cable failure in the field.

Numerous scholars worldwide have conducted extensive research on the bearing damage evolution and mechanical response of anchorage structures. Initial studies primarily focused on static pull-out behaviors, progressive failure mechanisms, and the impact of installation quality [7]. Researchers have established realistic trilinear bond-slip models [8] and explored the micro-failure process of resin-rock interfaces under various radial pressures [9]. Furthermore, the detrimental impacts of installation eccentricity [10] and resin gloving [11] have been comprehensively evaluated, leading to innovative reinforcement methods such as rotation-stirring and borehole expansion [12], as well as optimizations for surface-wound structures and varying anchorage lengths [13,14]. Building upon static mechanics, recent research has increasingly shifted towards structural integrity under dynamic and cyclic loading conditions. Studies have developed innovative numerical simulation methods [15] and tracked the debonding process of interfaces using advanced monitoring technologies like Fiber Bragg Grating (FBG) and Acoustic Emission (AE) [16]. For instance, Zhao et al. [3] distinguished dynamic cracking patterns using the DMKL algorithm, while Wang et al. [17] confirmed that under cyclic impact, damage accumulation is highly correlated with impact energy, with tensile cracks dominating approximately 85% of the internal failure. Furthermore, from an energy perspective, scholars have explored the dynamic responses and energy dissipation mechanisms of rocks surrounding anchored roadways subjected to impact loads [18]. Current constitutive models and empirical tests have successfully characterized the evolution patterns of total, elastic, and dissipated energy densities [19,20] ultimately guiding the development of novel energy-absorbing support components [21,22].

Despite these significant advancements, existing research predominantly focuses on the magnitude of impact energy or isolated impact events. This leaves a critical knowledge gap: the specific influence of disturbance frequency on energy response characteristics and interfacial debonding laws [23] remains insufficiently understood. In deep underground environments, dynamic loads possess distinct spectral signatures, such as low-frequency microseisms versus high-frequency blasting vibrations. To distinctly differentiate from previous studies that rely on generic dynamic impacts or passive field monitoring, this research explicitly isolates the disturbance frequency spectrum (10–200 Hz) as the core controlled variable.

Therefore, taking the 61203 working face of a mine in Ordos as the engineering background, systematic experiments were conducted using a custom-designed external active sinusoidal excitation test bench. By coupling macroscopic static/dynamic pull-out tests with mesoscopic PFC^2D^ numerical simulations, this study directly compares the capacity attenuation, energy transfer laws, and internal damage evolution modes (tensile vs. shear) across different frequency regimes. Ultimately, this research not only provides a refined theoretical basis for optimizing frequency-selective anti-impact support parameters in deep roadways, but also innovatively exploits these frequency-dependent debonding mechanisms to propose a novel method for the controllable debonding and green recovery of waste anchor cables.

## 2. Test survey

### 2.1. Engineering background

A coal mine in Ordos, Inner Mongolia Autonomous Region features deep coal burial, densely developed faults, and adopts blasting excavation. Superimposed with frequent mining-induced seismic disturbances, roadways suffer significant deformation, and the surrounding rock support structure is prone to fatigue failure under dynamic disturbance. The mine exploits the No. 6# coal seam, with an average thickness of 11 m, a burial depth of 500 m, and a dip angle of 2°. The coal seam structure is complex, and five hard sandstone layers (each >6 m thick) are developed within 100 m above the seam. Stratigraphically, the advance section of the working face is primarily characterized by fine-grained sandstone. Mechanical characterization indicates a uniaxial compressive strength (UCS) of 31.64 MPa, categorizing the roof lithology as a medium-hard stratum. Excavation activities, encompassing both roadway development and face advancement, are frequently punctuated by high-intensity dynamic load disturbances, which systematically induce seismic rock bursts and substantial roadway deformation. Observed instabilities are manifest as partial cable ruptures or slackening, pronounced roof mesh sagging, and significant floor heave. These manifestations of structural distress collectively undermine the integrity of the integrated support network and the surrounding rock mass.

Microseismic monitoring data of the mine’s 61203 working face from February to March 2024 show that the working face records 58 microseismic events per day on average, accounting for 75.5% of the mine’s total, indicating frequent disturbances to the anchorage structure. These microseismic events feature low frequency and high amplitude. The dominant frequency of 4 × 10^3^ J energy events is 0–80 Hz, while that of 5 × 10^3^ J events is 90–200 Hz. The total daily energy is below 6 × 10^3^ J, and the maximum single-event energy is less than 5 × 10^3^ J. Accordingly, the dynamic load vibration frequency range of the anchorage structure is determined as 10–200 Hz. To evaluate the anchorage performance of the surrounding rock and the anchoring system in the No. 6# coal seam, pull-out tests of 300-mm short encapsulated resin anchors were conducted in the 61203 auxiliary haulage roadway in October 2024, following GB/T 35056−2018 Technical Specification for Bolt Support of Coal Mine Roadway. Field support parameters were adopted: borehole diameter = 30 mm, matched with MSZ2335 anchoring agent and 1 × 19S-21.8 mm anchor cables. Test results indicate that under identical anchoring locations and cable diameters, the anchorage force of the solid coal rib is markedly higher than that of the gob-side rib. Specifically, the anchorage force reaches 146.52 kN for the solid coal rib, but only 130.26 kN for the gob-side rib in the 61203 auxiliary haulage roadway.

Given the frequent disturbances of 10–200 Hz low-frequency high-amplitude dynamic loads on the mine’s anchorage structures and the poor anchorage performance of gob-side surrounding rock, this study adopts the in-situ microseismic monitoring frequency range. End-anchored bodies under similar geological conditions were investigated. Anchorage specimens were fabricated using similar simulation methods. Laboratory tests and numerical simulations were combined to analyze the bearing capacity attenuation laws and vibration energy response characteristics of end-anchored bodies under various disturbance frequencies and anchorage lengths, and to reveal the internal damage evolution mechanism under dynamic load disturbances.

### 2.2. Testing apparatus

The pull-out load was applied to the anchorage body via a comprehensive mechanical property test system for mine support materials. In this device, the vertical hydraulic unit was mounted on the upper beam to supply supporting force for the upper chuck through upright columns. Cooperating with tension-compression sensors, the system can test the tensile performance of anchor cables and the pull-out strength of anchorage specimens. Key technical parameters are as follows: the vertical displacement measurement range is 500 mm; the maximum tensile and compressive capacities are 1000 kN and 1500 kN, respectively; the maximum tensile spacing is 1500 mm; and the loading speed ranges from 0.5 to 100 mm/min. During pull-out tests, anchorage specimens were fixed with auxiliary fixtures (Fig 2). A constant loading rate of 6 mm/min was adopted for the indoor tests within the above speed interval.

**Fig 2:**
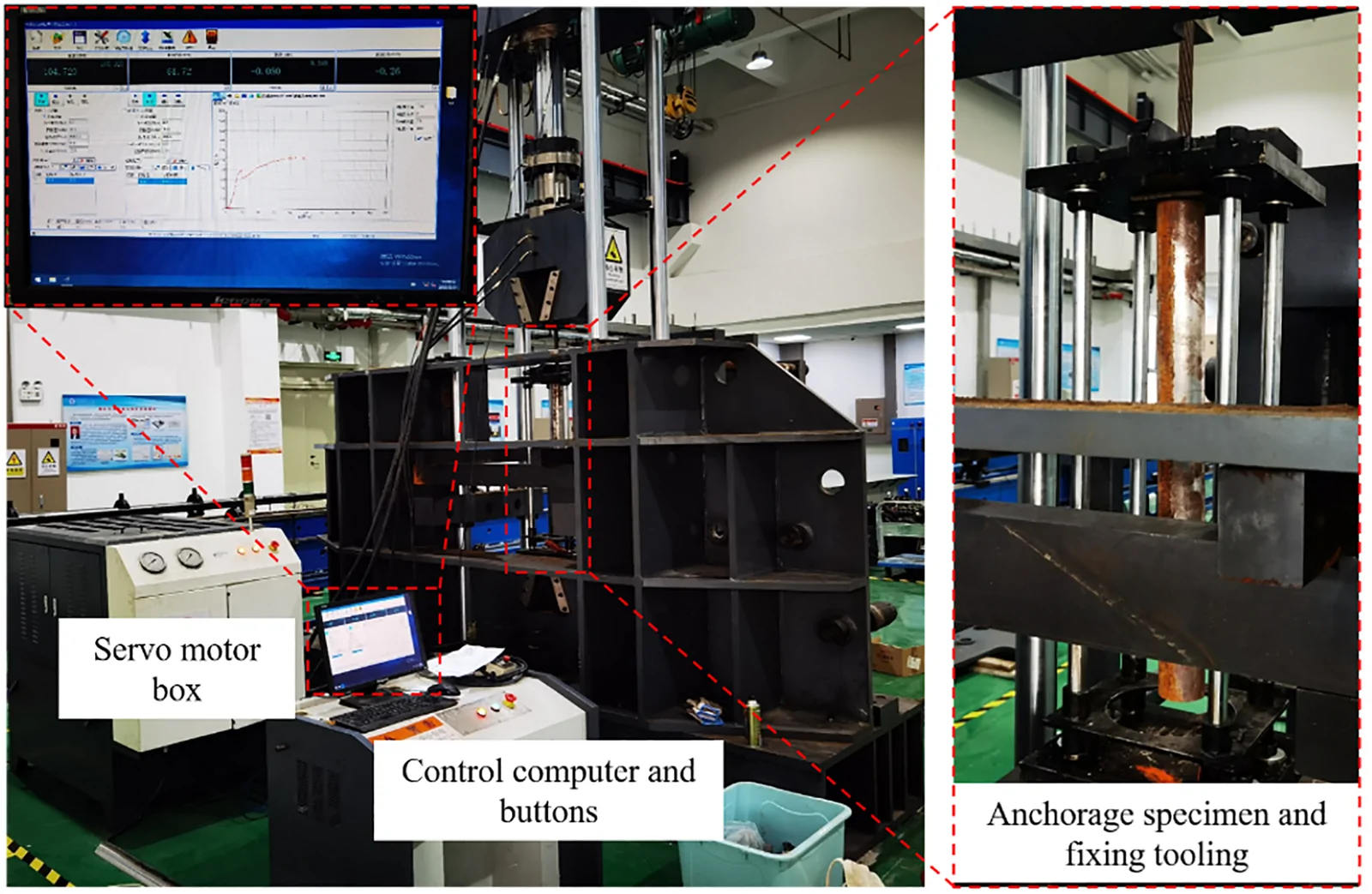
Comprehensive mechanical performance test apparatus for mine support materials.

A dedicated anchor cable vibration test bench was independently designed and manufactured to apply multi-frequency disturbances to anchorage structures. The system comprises a vibration table, an electromagnetic exciter, a power amplifier, control sensors, a control unit, a computer, and dedicated operating software. The natural frequency of specimens is tested using white-noise excitation and equal-amplitude sinusoidal frequency sweep. Vibration signals are collected by piezoelectric acceleration sensors and converted from the time domain to the frequency domain via Fourier transform, to analyze the energy response of the free segment of anchorage bodies [24].The test bench has four detachable fixing holes to fasten the outer steel tube and prevent detachment during tests. The exciter is laterally restrained, adapting to specimens of different lengths and reducing damage from transverse vibration. It provides a maximum excitation load of 500 N and operates at 5–3000 Hz, covering the 10–200 Hz test range. The external load excitation test bench is presented in Fig 3.

**Fig 3:**
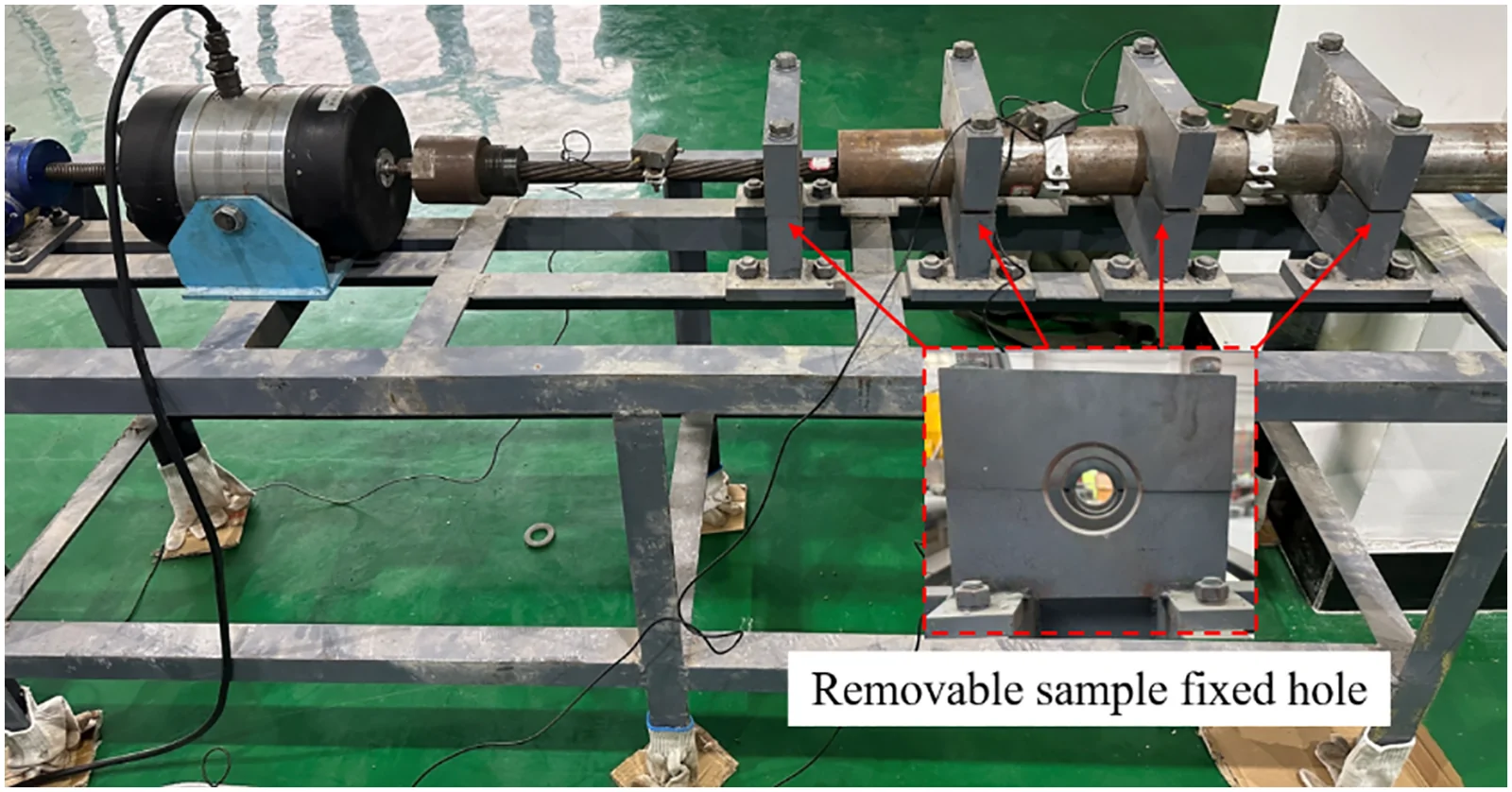
Anchor cable anchorage and external excitation test bench.

During excitation, an axial sinusoidal load with a constant force amplitude was applied to the anchor cable’s free end to standardize boundary conditions. Since the exciter’s output force is linearly proportional to the input current (*F* ∝ *I*), this constant force was achieved by maintaining a fixed current. Importantly, while the force remains constant, the mechanical energy transferred into the anchorage system is non-linear and frequency-dependent. This is because the system’s varying mechanical impedance (e.g., inertia and dynamic stiffness) generates different displacement amplitudes under a constant force. This design enables the precise investigation of the system’s energy absorption, vibration transmission, and fatigue damage under a uniform dynamic force across the 10–200 Hz spectrum.

### 2.3. Anchorage specimen preparation

Given that the roof of the working face advance section is dominated by fine-grained sandstone with a UCS of 31.64 MPa, it is classified as a medium-hard stratum. M30 cement mortar was selected as the matrix material, and a water-cement-sand ratio of 0.5:1:3 was adopted to simulate in-situ surrounding rock. Uniaxial tests confirmed that the strength of the mixed material matched that of underground rock core samples, which determined the optimal proportion of the anchorage matrix similar material. The cement mortar pouring length was set to 800 mm. A hollow steel pipe with an outer diameter of 77 mm, inner diameter of 68 mm, wall thickness of 9.0 mm and length of 800.0 mm was used as the container for similar materials, and the designed anchorage length was 300 mm. A medium-speed resin anchoring agent with a cartridge diameter of 23 mm was adopted, and the anchor cable length was 1200 mm. To reserve the anchoring hole, a 30.0 mm thin-walled steel pipe was coated and centrally fixed inside the container through a bottom centering ring. After being weighed and evenly blended according to the mix ratio, the similar materials were layered and vibrated for compaction, with secondary compaction conducted after pouring. Following initial setting, the thin-walled steel pipe was extracted to form the anchoring hole. All specimens were water-cured for 28 days before anchorage tests. The specimen preparation process is presented in Fig 4.

**Fig 4:**
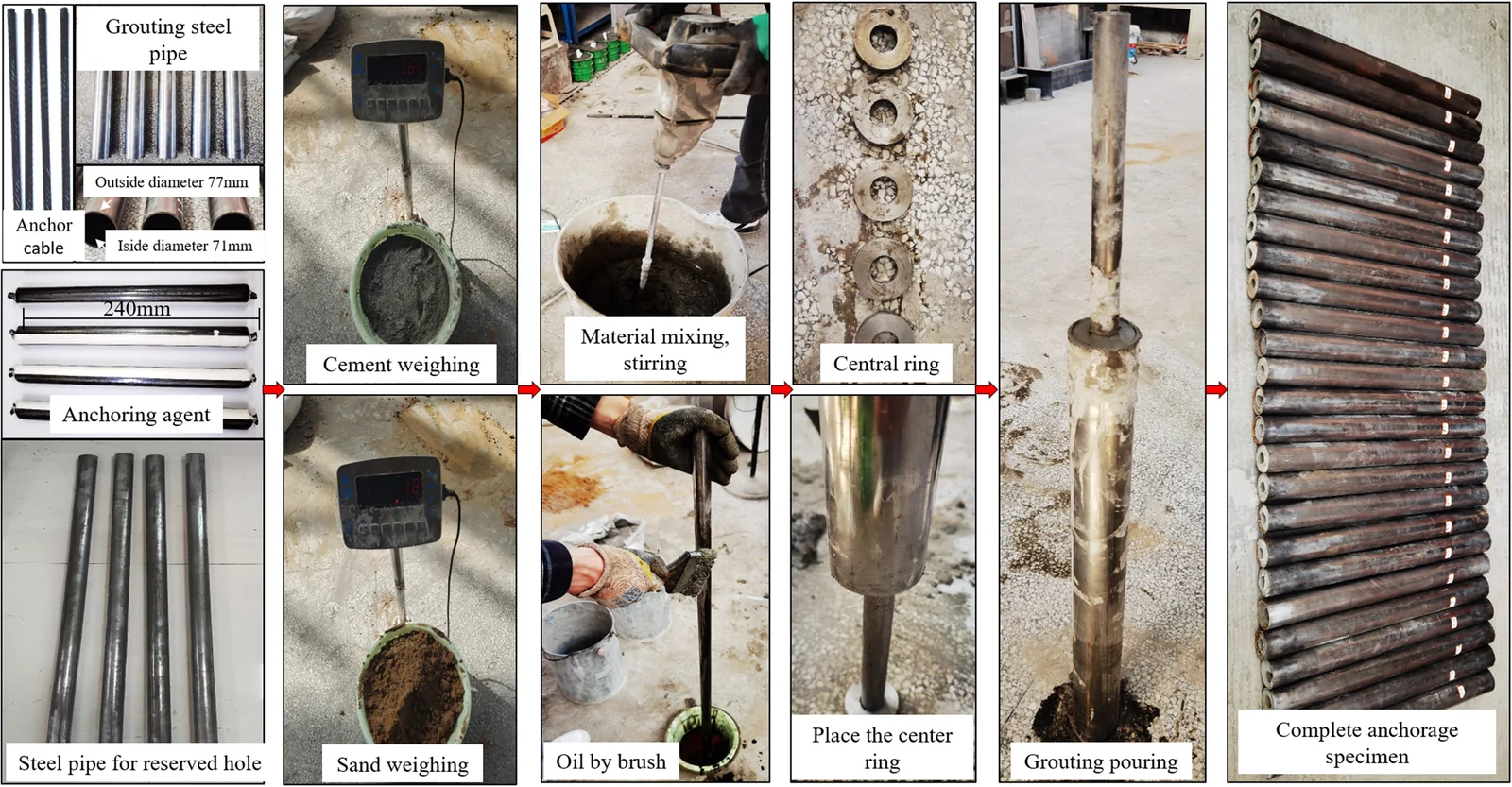
Preparation of test materials and anchorage matrix.

The steel pipe anchorage matrix, cast with cement mortar similar surrounding rock materials, was moist-cured for 28 days before specimen anchorage. The anchorage length and free section length were designed in proportion to the in-situ dimensions. The total length of the anchor cable used in vibration tests was 1200 mm. To determine the appropriate dimension of the anchoring agent, the three-diameter matching principle detailed in formula (1) was utilized.


$$\begin{array}{c} l_{\text{r}}=\frac{{D_{\text{m}}}^{\text{2}}-{d_{\text{s}}}^{\text{2}}}{{d_{\text{r}}}^{\text{2}}}l_{\text{a}} \end{array}$$ (1)

Where lr is the required anchorage agent length, mm; la is the design anchorage length of the anchorage sample, mm; dr is the diameter of resin anchoring agent, mm; Dm is the diameter of anchorage hole, mm; ds is the nominal diameter of anchor cable, mm.

In the experimental setup, 1200-mm-long 1 × 19S anchor cables with a nominal diameter of 21.8 mm were utilized. Grouting was performed using an MSZ2335 medium-speed mining resin. The fundamental mechanical properties of this resin featured an elastic modulus of 16 GPa and a bonding strength of 17 MPa. Its curing kinetics dictated a gelation window of 90–180 s, a mechanical mixing phase of 20–35 s, a setting interval of 480 s, and the attainment of full load-bearing capacity at 30 minutes. Given a borehole diameter of 30 mm and the specified cable diameter, the necessary resin quantity was determined via the established three-diameter matching principle. Accordingly, to achieve the designated embedment depths of 300 mm and 600 mm, the required lengths of the resin cartridges were calculated to be 240 mm and 480 mm, respectively.

When preparing the anchorage specimens, the steel pipe specimens with pre-reserved boreholes were first placed horizontally on a self-made test frame and secured with bolts via fixed fixtures. To prevent insufficient effective anchorage length caused by the overflow of resin anchoring agent during the anchoring process, wooden plugs were used to seal the anchoring section of the hollow boreholes in the steel pipes. According to the test-designed anchorage length and borehole diameter, the required amount of anchoring agent was calculated using Formula (1). Specifically, anchoring lengths of 300 mm and 600 mm required anchoring agent lengths of 240 mm and 480 mm, respectively (corresponding to 2 and 3 pieces of MSZ2335 anchoring agent, each with a standard length of 230 mm). The anchoring agent was cut as per the calculation results. In this test, MSZ2335 medium-speed mine resin anchoring agent was adopted, and the cut anchoring agent was calibrated before being loaded into the boreholes. The end of the anchor cable was connected to a pneumatic anchor cable drilling rig via an anchor cable agitator. Prior to anchoring, the anchor cable was pre-inserted into the borehole, and label paper was used to push it to the specified depth, ensuring the anchor cable reached the bottom of the borehole. The air inlet valve of the air compressor was opened, and the high-pressure airflow was adjusted through the joystick switch to control the operation of the drilling rig. The high-pressure gas entered the rotary head of the drilling rig through the pipeline, driving the anchor cable to rotate and advance. After the anchor cable reached the bottom of the borehole, continuous stirring was maintained for 1 minute before stopping. After the anchorage was completed, the specimens were grouped and numbered, as shown in Fig 5.They were cured at room temperature for 1 week, after which the vibration tests of the anchor cable anchorage bodies were conducted.

**Fig 5:**
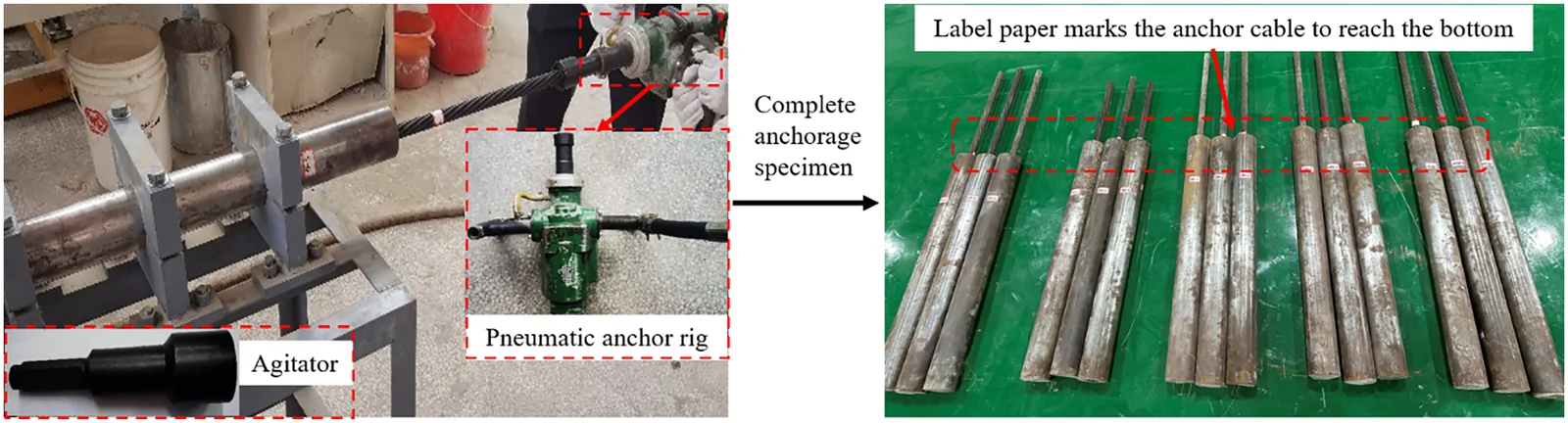
Anchoring specimen and complete specimen.

### 2.4. Experimental design

To explore the effects of different disturbance frequencies on the damage characteristics and bearing capacity of end-anchored structures, static pull-out tests were firstly conducted with displacement-controlled loading [25] at a loading rate of 6 mm/min (Fig 6). Three parallel specimens, numbered J-1–J-3, were tested, and the average peak pull-out strength was recorded. The test scheme is summarized in Table 1. For an anchorage length of 300 mm, disturbance frequencies were set as 10 Hz, 50 Hz, 100 Hz, 150 Hz and 200 Hz, while a frequency of 50 Hz was adopted for a 600 mm anchorage length. In this research, 10–50 Hz was defined as medium-low frequency, and 100–200 Hz as high frequency. Sinusoidal waveforms were applied for dynamic disturbance loading (Fig 7), and tests were implemented by adjusting the disturbance frequency. The disturbance tests contained seven groups, with three replicated anchorage specimens in each group, and a total of 21 tests were completed.

**Table 1:** Vibration test scheme.

Group	Specimen number	Configuration loading	Excitation frequency *f/*Hz	Remark
**1**	J-1, J-2, J-3	static load drawing	–	the peak pull-out strength of the test specimen was tested.
**2**	D10-1, D10-2, D10-3	external excitation of free end of anchor cable	10	the waveform is a sine wave
**3**	D50-1, D50-2, D50-3	external excitation of free end of anchor cable	50	the waveform is a sine wave
**4**	D100-1, D100-2, D100-3	external excitation of free end of anchor cable	100	the waveform is a sine wave
**5**	D150-1, D150-2, D150-3	external excitation of free end of anchor cable	150	the waveform is a sine wave
**6**	D200-1, D200-2, D200-3	external excitation of free end of anchor cable	200	the waveform is a sine wave
**7**	M50-1, M50-2, M50-3	external excitation of free end of anchor cable	50	the waveform is a sine wave, anchorage length 600 mm

**Fig 6:**
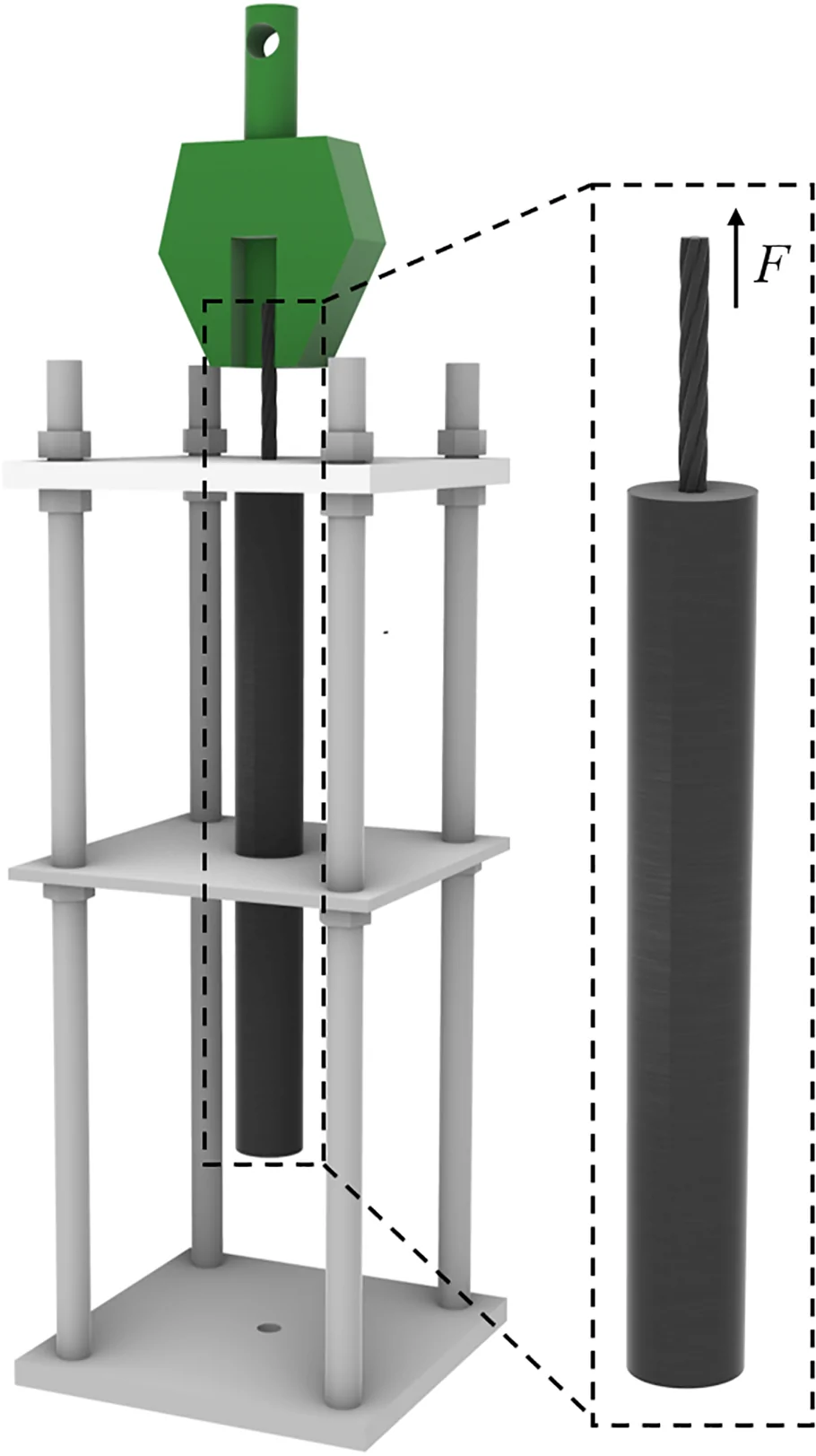
Static load anchorage pull-out.

**Fig 7:**
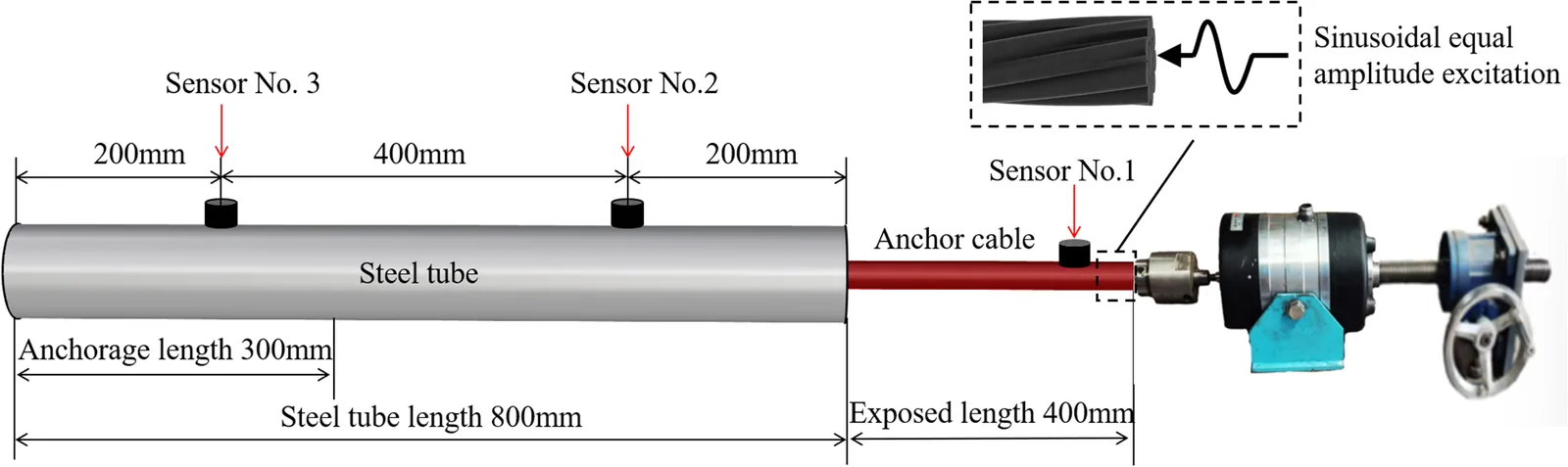
Schematic diagram of acceleration sensors and load application setup.

As illustrated in Fig 8, dynamic monitoring utilized YD21 piezoelectric accelerometers (0.2–4000 Hz bandwidth, 10 pC/(m·s⁻²) charge sensitivity, and ≤5% transverse sensitivity ratio). To capture vibration responses, individual accelerometers were respectively deployed on the anchor cable’s free segment, and on the steel pipe at locations 200 mm from its upper and lower ends. Fig 7 also details the complete configuration for applying external sinusoidal excitations. To establish a baseline, the initial average peak pull-out capacity was evaluated prior to dynamic loading. After a 600-s continuous excitation, direct extraction tests were executed to ascertain the residual pull-out resistance. This specific excitation duration was quantitatively aligned with the equivalent cumulative fatigue induced by transient in-situ microseisms. By maintaining a constant loading time, the experimental protocol rigorously decouples the frequency effect, fundamentally preventing multi-variable confounding. This structured protocol enabled a systematic comparative evaluation of anchoring strength degradation and energy dissipation behaviors across varying disturbance frequencies [26].

**Fig 8:**
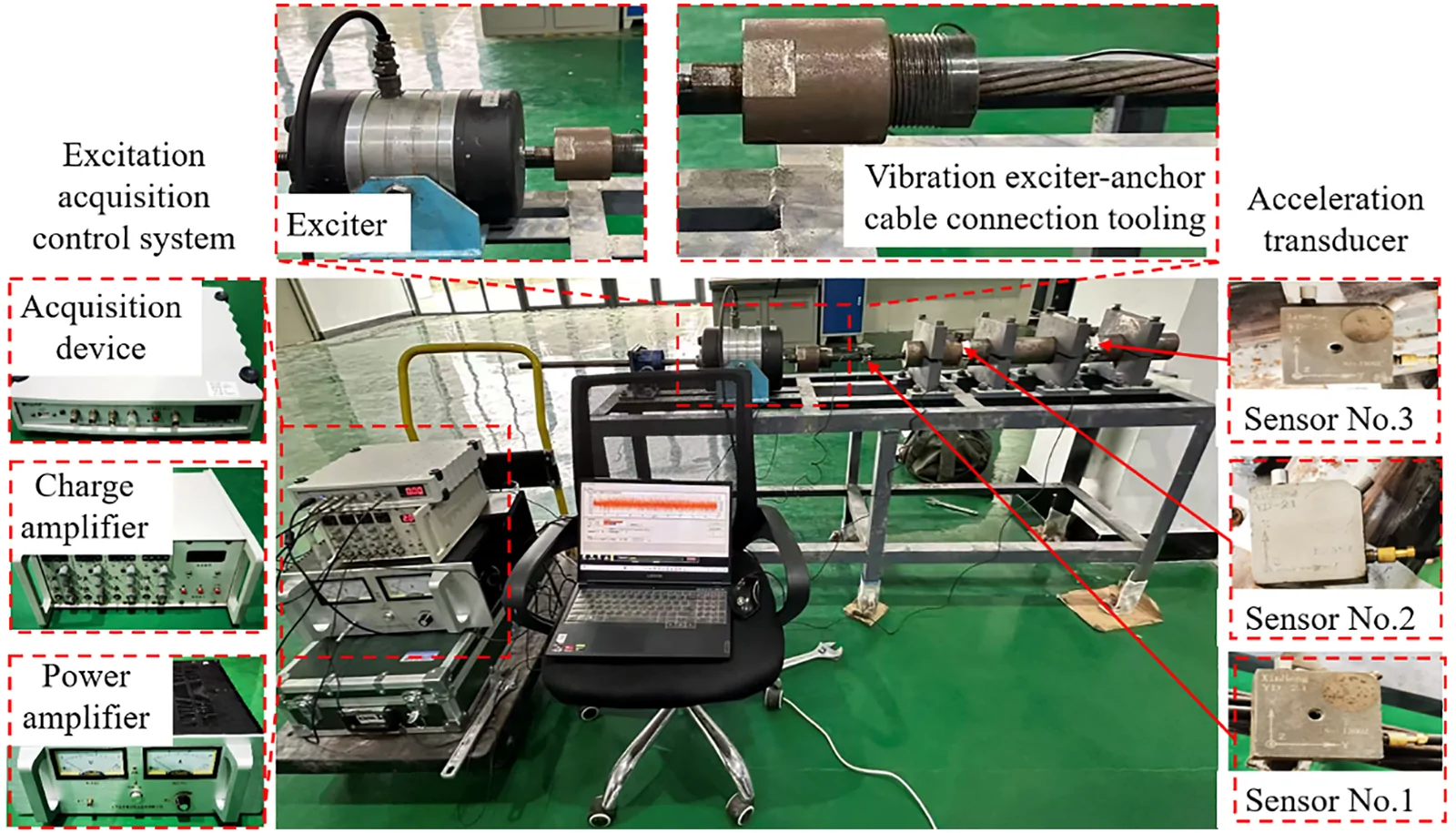
External sinusoidal load excitation test system of anchor cable anchorage specimen.

## 3. Bearing capacity and energy response characteristics of anchorage bodies

### 3.1. The peak pull-out strength of the specimen under static load pull-out condition

Following the mechanical trials, the static load-displacement profiles of the anchor cables were generated from the acquired data. To provide a rigorous basis for cross-configuration comparison, the absolute pull-out displacement (*S*) was normalized against the corresponding initial anchorage length (*L*_a_) of each specimen to define a dimensionless parameter: normalized displacement (*S*/*L*_a_). Specifically, the absolute displacement values were divided by 300 mm and 600 mm for specimens with 300 mm and 600 mm anchorage lengths, respectively. This conversion allows for a unified evaluation of the relative interfacial slip. Consequently, the baseline mean peak pull-out capacity of the end-anchored assemblies was established at 140.18 kN (Fig 9). From the curve, only the J-2 anchorage specimen’s pull-out curve exhibits a “bimodal” feature, all of which belong to the bond failure-friction reduction type [27]. Within a normalized displacement (*S*/*L*_a_) of 0.018, the anchoring agent undergoes elastic deformation; between 0.018 and 0.071, it transitions from slip deformation to debonding-slip. In the slip deformation stage, the load of the anchorage specimen rises rapidly, and complete debonding occurs at a normalized displacement of approximately 0.184. Under static loads, the maximum peak pull-out loads of the three specimens are 138.96 kN, 145.26 kN, and 136.32 kN, with an average of 140.18 kN, which serves as the standard for evaluating the anchorage force attenuation under different external excitation frequencies.

**Fig 9:**
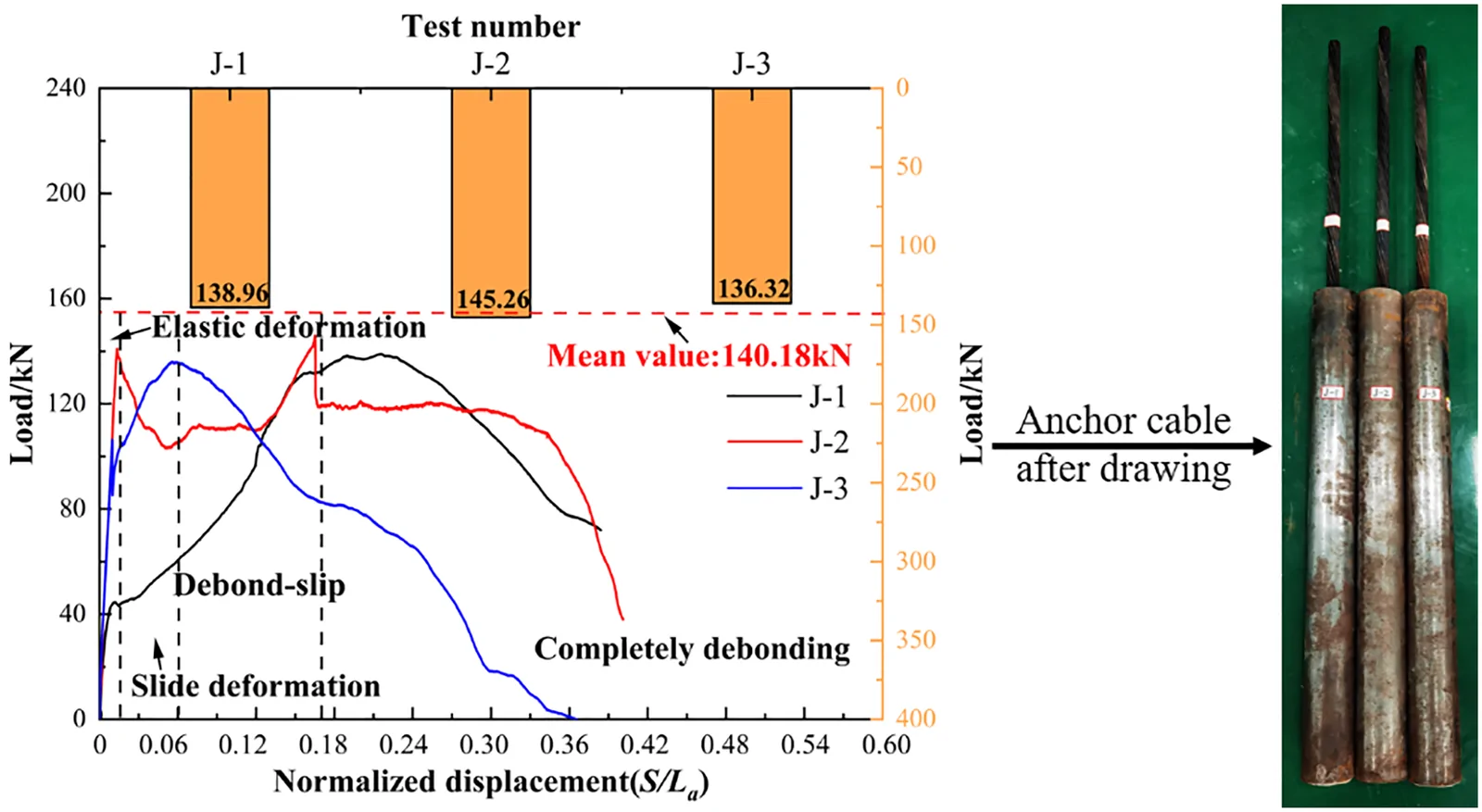
Normalized load-displacement curves under static load and post-test displacement of end-anchored cables.

Post-test anchorage specimens are shown in Fig 9. No obvious surface changes were observed on the steel pipes after pull-out tests. The anchoring agent fully solidified after loading, making the steel pipes unreusable for subsequent tests.

### 3.2. Attenuation law of anchorage strength under different disturbance frequencies

To explore the effects of different disturbance frequencies on the bearing performance of end-anchored bodies, static pull-out tests were performed on specimens subjected to sinusoidal excitation at 10 Hz, 50 Hz, 100 Hz, 150 Hz and 200 Hz. The anchorage force attenuated slightly under 150 Hz and 200 Hz excitation, with damage parameters of only 0.064 and 0.051. The load-displacement curves and key test characteristics are shown in Fig 10. Curves differed greatly after slip deformation initiated during pull-out, yet all specimens presented the bond failure and friction reduction mode. Elastic deformation occurred within a normalized displacement of 0.018, followed by a transition from elastic deformation to slip deformation at 0.018–0.067. In the slip stage, the load rose rapidly. Complete debonding appeared at a normalized displacement of around 0.184, and the anchor cables finally failed by overall debonding.

**Fig 10:**
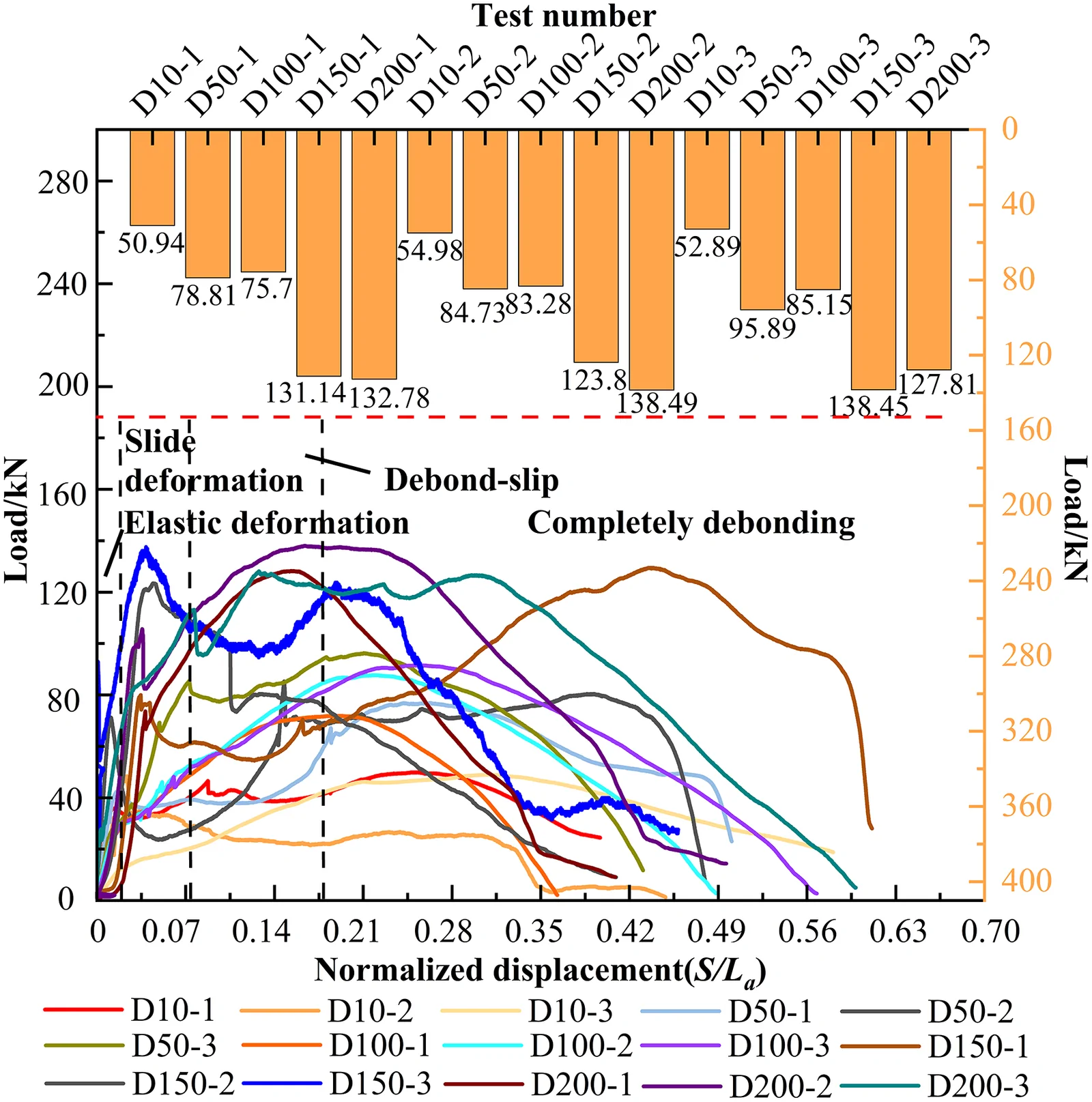
Normalized load-displacement curves of anchorage pull-out after sinusoidal load excitation at different disturbance frequencies.

Pull-out test results reveal that the peak pull-out strength of anchored bodies is significantly positively correlated with disturbance frequency, and bearing capacity attenuation decreases obviously with increasing frequency. Under low-frequency excitation at 10 Hz, specimen curves display mixed unimodal and bimodal features. The average peak pull-out strength is only 52.94 kN, 62.23% lower than that under static loading, leading to the most serious damage. For medium-low frequencies of 50 Hz and 100 Hz, the average peak strength is 86.48 kN and 81.28 kN, with bearing capacity attenuation of 38.74% and 42.02%, respectively. Specimens subjected to high-frequency excitation (150 Hz and 200 Hz) present typical bimodal curves. Their average peak strength reaches 131.13 kN and 133.03 kN, with attenuation rates of merely 6.46% and 5.10%, which exert little adverse effect on the overall bearing performance of the anchorage system. Low-frequency disturbance greatly increases slip and debonding displacement and triggers extensive interfacial damage. By contrast, high-frequency disturbance accelerates energy dissipation, confines interfacial damage to local regions, and thus has a limited influence on comprehensive anchorage performance.

Pull-out tests were conducted on end-anchored bodies after excitation at different disturbance frequencies. The post-test specimens are shown in Fig 11. Obvious cracks appeared at the bottom of the steel pipes, whereas no notable overall deformation occurred. The anchor cable pull-out displacement is defined as the distance from the label to the steel pipe top.

**Fig 11:**
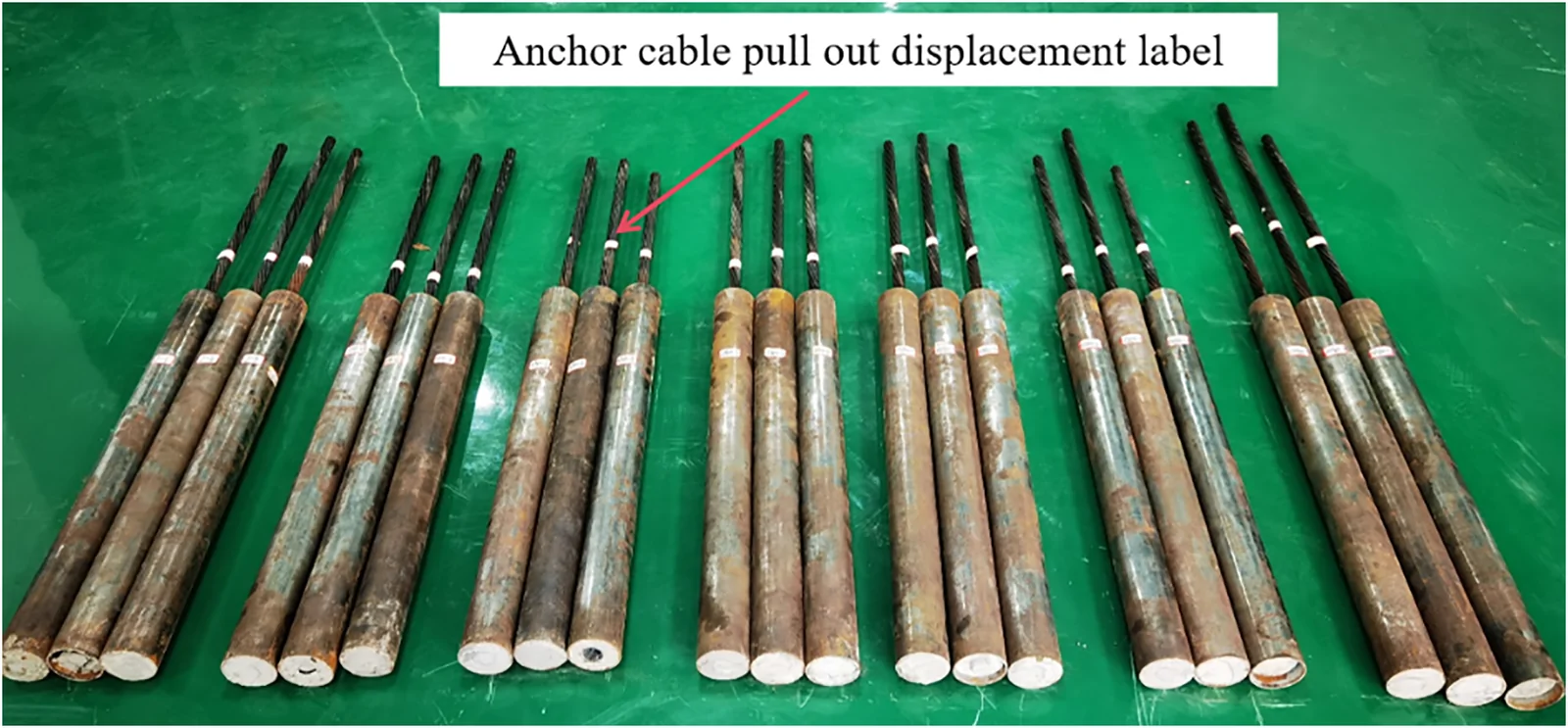
Displacement of anchor cables and steel pipe deformation after external sinusoidal excitation.

### 3.3. Damage parameter analysis

To systematically evaluate the performance degradation of the anchored system induced by repetitive dynamic loading, a quantitative damage variable, *D*, is formulated. An elevated *D* value directly correlates with a more pronounced loss in load-carrying capability, indicating an exacerbated state of structural deterioration [28]. This metric is mathematically expressed as:


$$\begin{array}{c} D=\frac{F-F_{\text{d}}}{F} \end{array}$$ (2)

Where, *F* represents the baseline mean ultimate pull-out capacity of the intact specimens (established at 140.18 kN via quasi-static reference testing); while *F*_d_ designates the residual mean peak extraction resistance (in kN) measured following the application of cyclic excitations.

The statistical results of peak pull-out strength of anchorage bodies under different external excitation frequencies are presented in Fig 12. Obvious differences exist in the peak strength of specimens subjected to the five excitation frequencies. With the rise of excitation frequency, the peak pull-out strength increases gradually, and the damage degree of anchorage bodies is reduced accordingly.

**Fig 12:**
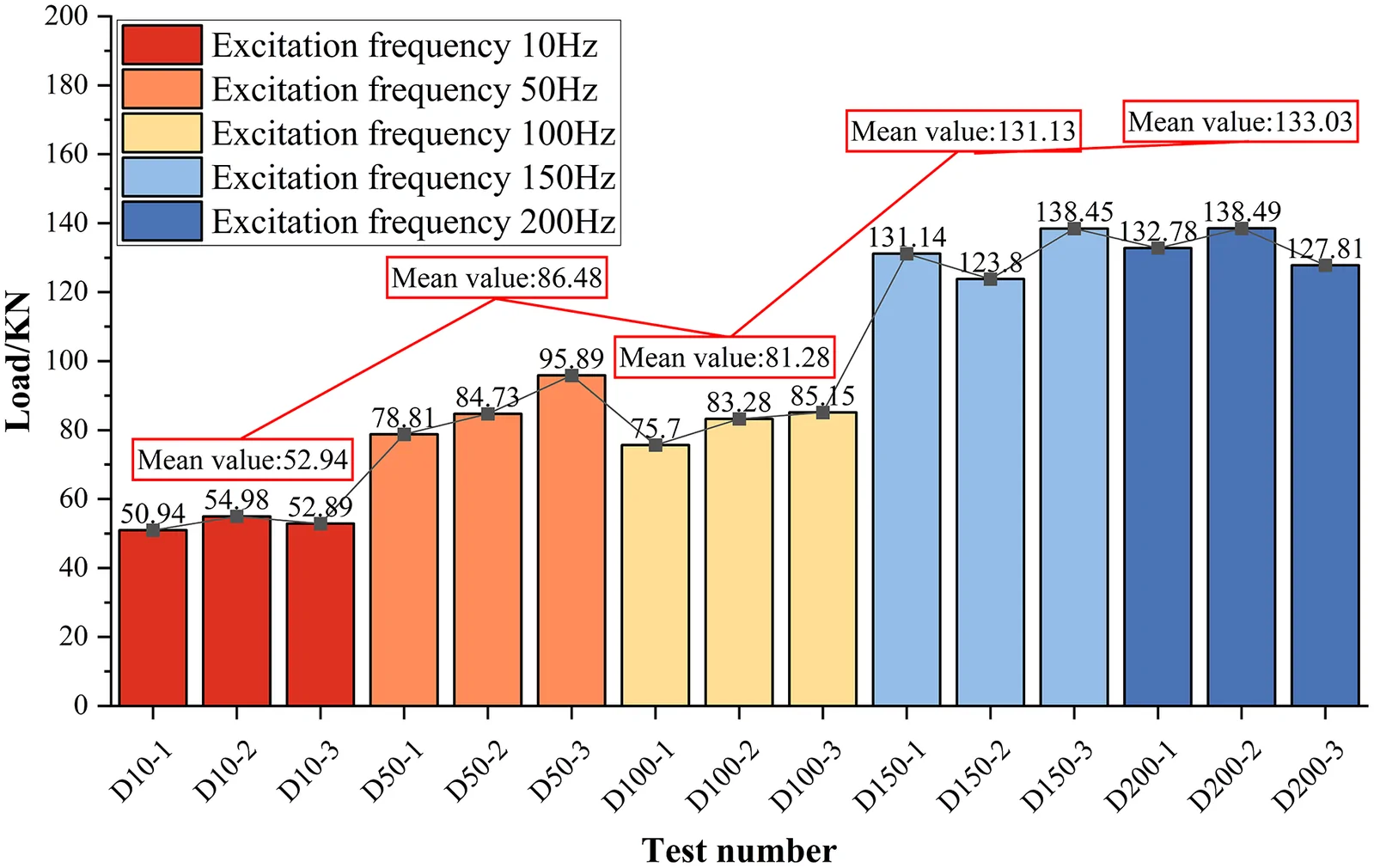
Peak pull-out strength under different disturbance frequency excitation.

The fitting curve of excitation frequency and damage parameter is presented in Fig 13. The damage parameter *D* decreases linearly with increasing frequency. To statistically evaluate this linear trend (y = −0.00303x + 0.61723), an error analysis was conducted, yielding a coefficient of determination (*R²*) of 0.832 and a Root Mean Square Error (RMSE) of 0.078, indicating reliable predictive accuracy. It must be strictly noted that this formula holds valid specifically within the 10–200 Hz range under the current baseline energy input; extrapolating beyond 200 Hz is invalid due to the transition towards non-linear, localized failure mechanisms. Quantitatively, the values of *D* at 10, 50, and 100 Hz are 0.622, 0.383, and 0.421, respectively, whereas high-frequency excitations (150 and 200 Hz) yield *D* values of merely 0.064 and 0.051. Consequently, low-frequency disturbances (10–100 Hz) induce severe anchorage damage, while high-frequency excitations exert a minor impact on pull-out strength.

**Fig 13:**
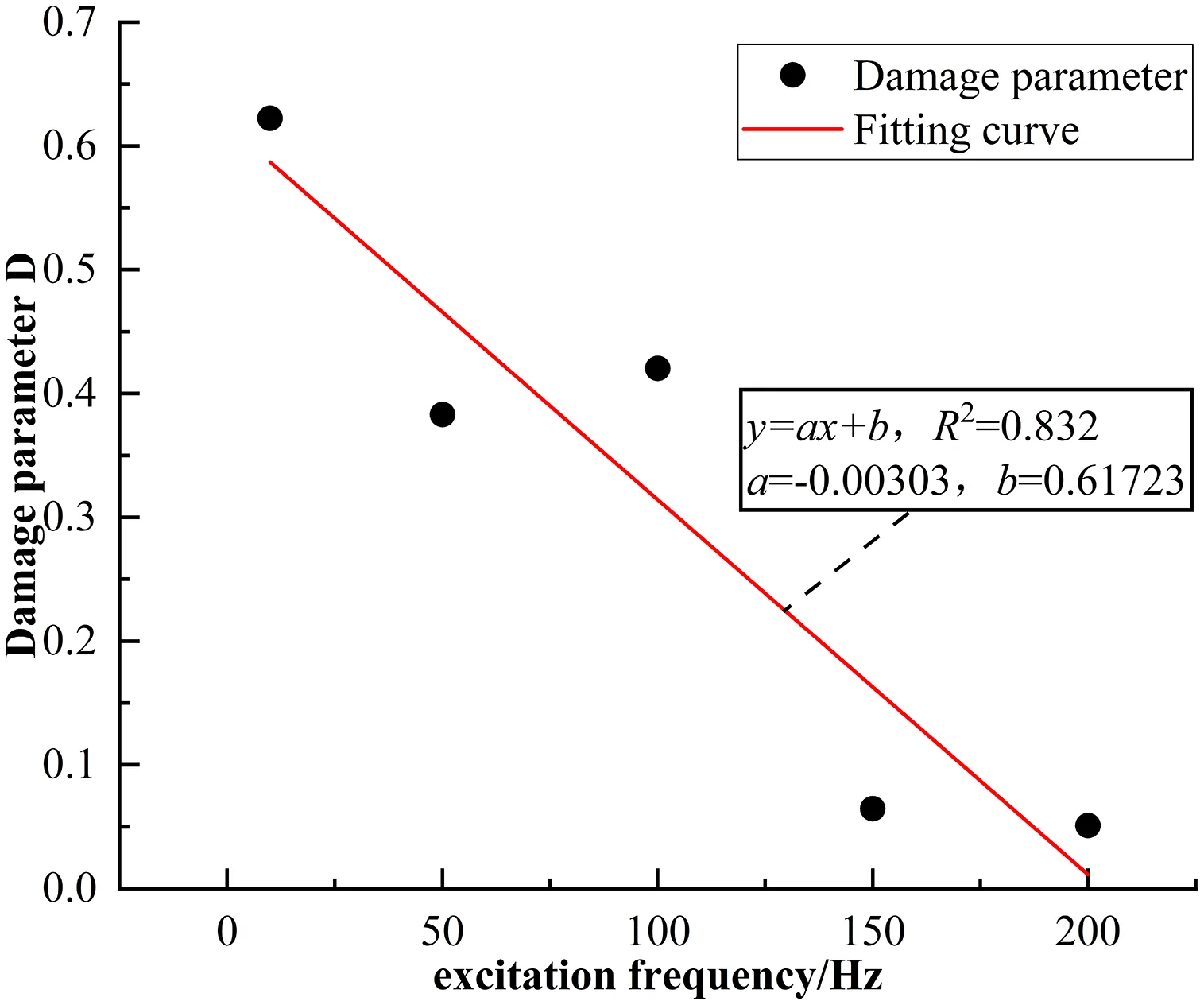
Variation of damage parameters with excitation frequency.

Mechanistically, under low-frequency excitation (e.g., 10 Hz), the longer action duration promotes stress accumulation, driving internal microcracks to fully expand and coalesce, which significantly accelerates rock damage and results in a higher *D* value [29]. Conversely, under high-frequency excitation, the rock mass exhibits an obvious strain hardening effect. The inertial effect under short-period dynamic loading restricts rapid crack penetration, confining damage to local areas rather than causing large-scale structural failure, thus preserving overall anchorage performance [30,31]. In terms of roadway support and blasting vibration control, these findings dictate that long-term low-frequency vibrations on anchorage structures must be strictly avoided, while localized damage induced by high-frequency vibrations still requires attention to optimize field support schemes [32].

### 3.4. Energy response analysis of free section of anchor cable under different disturbance frequencies

Given the non-stationary, nonlinear and time-varying characteristics of energy response signals in the free section of anchor cables under different vibration frequencies, time-frequency analysis was adopted in this study [33,34]. Building upon the standard Fourier framework, the Short-Time Fourier Transform (STFT) effectively extracts localized features within the joint temporal-spectral domain. Conventional Fourier transform, a linear integral transformation, decomposes complex signals into sinusoidal components for spectral analysis, and the discrete Fourier transform is the most widely applied type. Let the original signal be *x*(*t*); its Fourier transform formula is defined as follows:


$$\begin{array}{c} X\left(\omega \right)=\int x\left(t\right)e^{-i\omega t}dt \end{array}$$ (3)

Where: *X*(*ω*) is the frequency of the original signal *x*(*t*), *t* is the time, *ω* is the angular frequency. The inverse transformation is:


$$\begin{array}{c} x\left(t\right)=\int X\left(\omega \right)e^{i\omega t}d\omega \end{array}$$ (4)

The Fourier transform enables mutual mapping and conversion between time-domain and frequency-domain signals, providing theoretical support for signal processing. To address the time-varying characteristics of the signal, the STFT introduces a time window function, which segments the time-domain signal and assumes the signal within each window is stable before performing the Fourier transform on each segment separately [35]. The STFT expression for signal *x*(*t*) is as follows:


$$\begin{array}{c} F\left(t,\omega \right)=\int x\left(\tau \right)g(\tau -t)e^{-i\omega t}d\tau \end{array}$$ (5)

Where: the window function *g*(*t*) performs windowed translation processing on the signal at time t, to establish the function of the relationship between time and frequency. Through *F*(*t, ω*), the time-frequency characteristics of the vibration signal at time t are explained.

To reveal the vibration response and energy transfer law of anchored bodies under varying disturbance frequencies, acceleration signals of the anchor cable free segment subjected to sinusoidal excitations at 10, 50, 100, 150, and 200 Hz were monitored and analyzed. To facilitate a fair and direct comparison across test configurations, the absolute measurement time (*t*) was normalized by the total constant excitation duration (*T* = 600 s) to define a dimensionless parameter: normalized time (*t/T*). As reflected in the normalized time-domain (displaying a stable vibration window of *t*/*T* = 0.080 ~ 0.100) and frequency-domain waveforms in Fig 14, 15, the vibration amplitude of the free segment decreases markedly with increasing excitation frequency. The peak accelerations of the frequency response curves are 15.2 g, 5.31 g, 3.43 g, 2.18 g, and 3.15 g under the five respective frequencies, yielding a 79.28% overall amplitude attenuation from 10 Hz to 200 Hz. The dominant vibration frequency under all working conditions remains stable within 700–1000 Hz, without obvious deviation induced by external excitation. Furthermore, spatial monitoring indicates that the steel pipe near the anchorage segment presents a higher vibration amplitude, whereas the amplitude drops significantly near the free segment. Time-domain signals were further processed via the Fourier transform to acquire spectral characteristics. The results demonstrate that high-frequency vibrations attenuate rapidly during propagation from the exciter to the anchorage segment. This rapid decay restricts the transmission distance and limits large-scale energy accumulation, thereby causing only minor internal damage to the anchorage system [36]. By comparison, low-frequency excitations exhibit superior energy transfer efficiency, which readily induces continuous and progressive deterioration of the anchorage interface.

**Fig 14:**
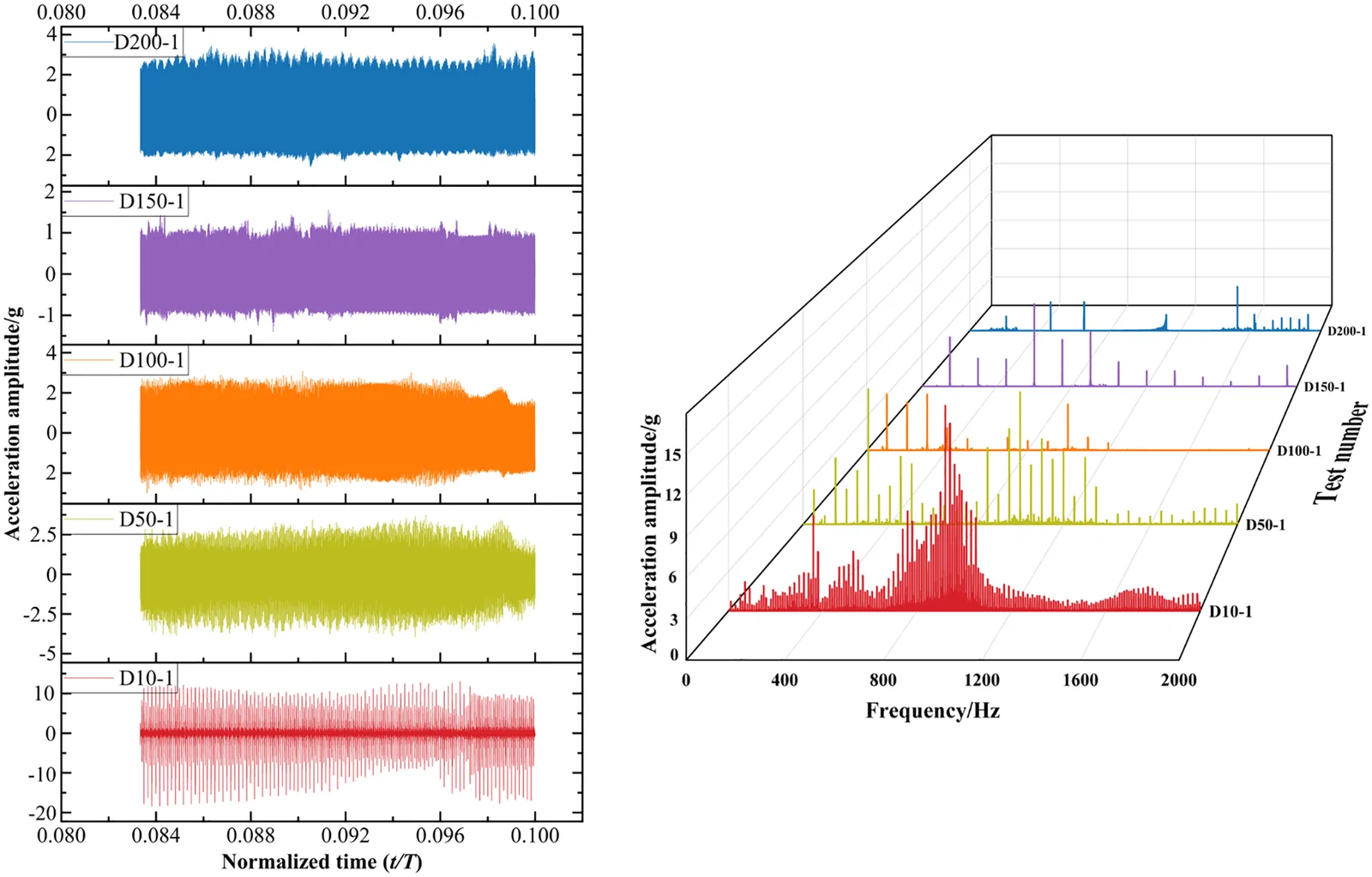
Normalized time-domain acceleration waveform.

**Fig 15:**
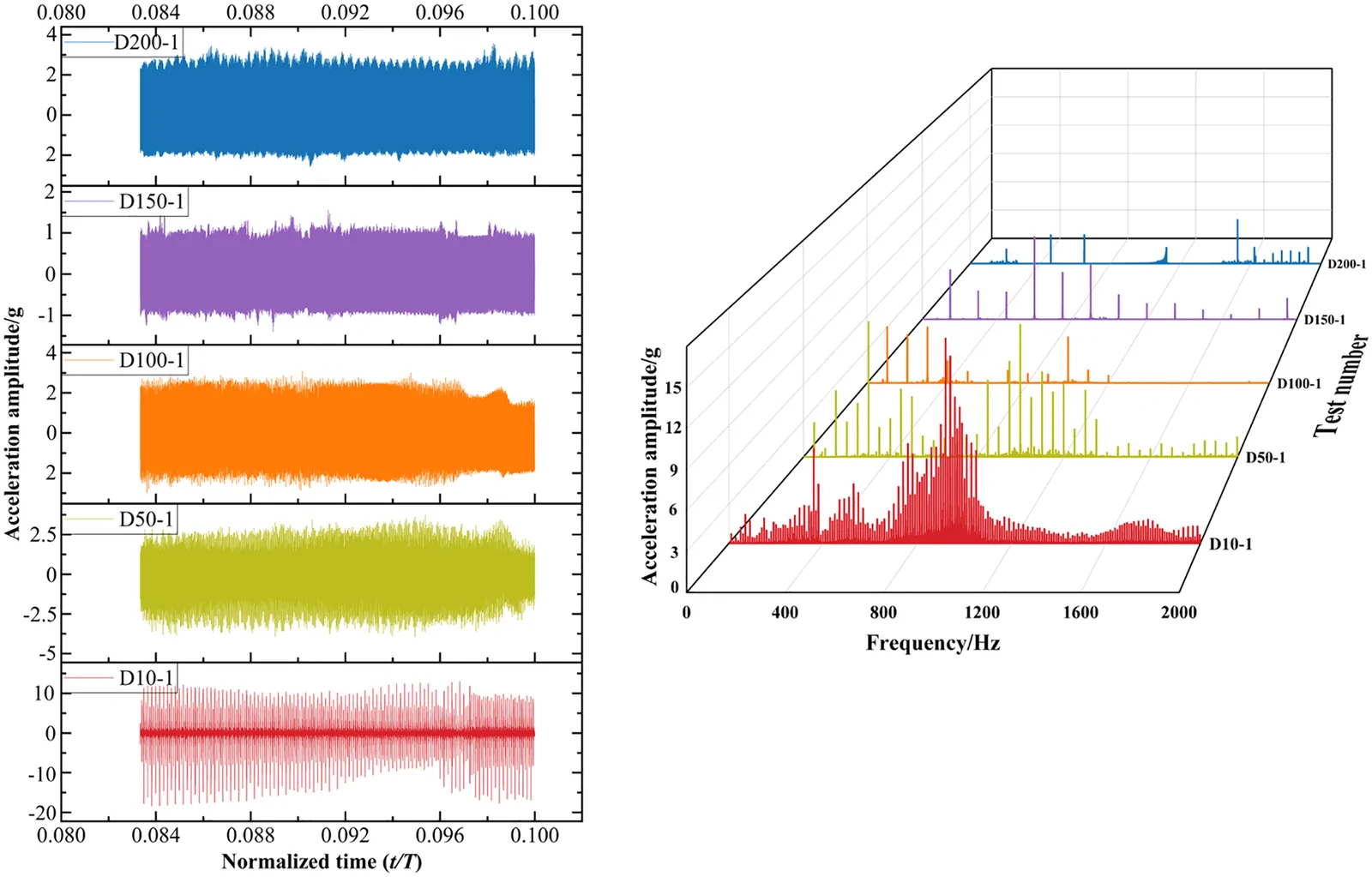
Frequency-domain acceleration waveform.

Statistical analysis was performed on the Root Mean Square (RMS) values of time-domain signals under each working condition (Fig 16). The results show that the overall vibration intensity decays with increasing excitation frequency. The quadratic polynomial fitting delivers a goodness-of-fit of *R*^2^ = 0.961, with the fitting equation expressed as *y* = (4.04*E* − 5)*x*^2^ − 0.0173*x* + 1.9127. Higher RMS values under low-frequency excitation indicate a stronger vibration response of the anchorage body. By contrast, the RMS value decreases significantly under high-frequency excitation. This is closely related to the inertia and damping effect of the anchorage structure, as well as the reduced transmission efficiency of high-frequency vibration on the anchor cable surface [37].

**Fig 16:**
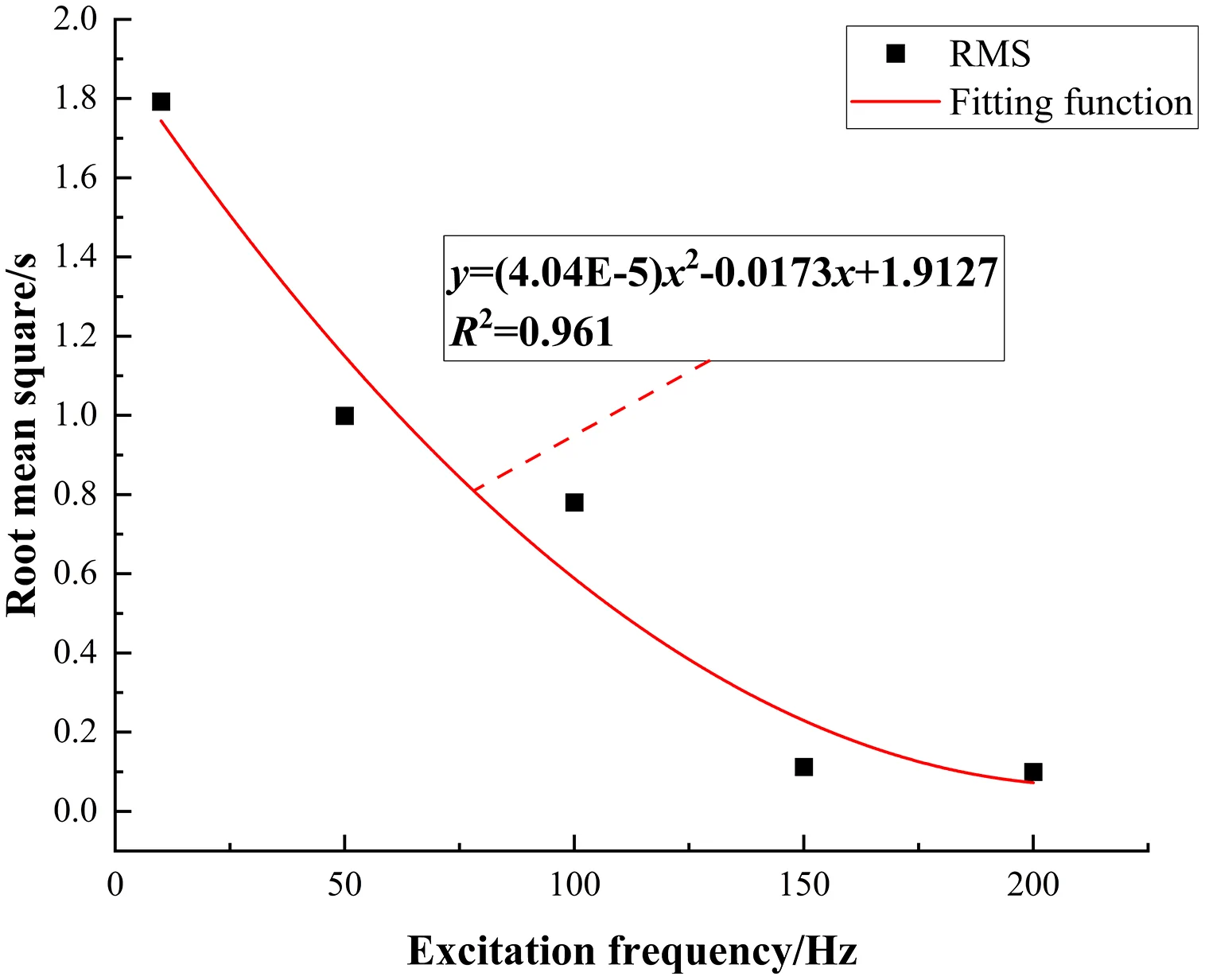
Fitting result of RMS and excitation frequency.

The energy response of the anchor cable free segment is a core characterization of dynamic damage and interfacial debonding in anchorage systems [38]. Unlike field passive vibration, in which energy transfers from surrounding rock to anchor cables, this study adopts an active sinusoidal excitation mode. In this mode, vibration energy propagates forward from the free segment to the anchorage segment, enabling precise regulation of energy input and further clarifying the interfacial debonding mechanism. Under dynamic disturbance, the interface between the anchoring agent and surrounding rock serves as the weak link of the anchorage system due to lower bonding strength and undergoes debonding failure preferentially. In contrast, the anchor cable–anchoring agent interface has higher bonding strength and suffers less severe damage. Under low-frequency disturbance (10 Hz), vibration waves travel a long distance with sufficient energy dissipation, triggering progressive debonding and large-scale failure at the anchoring agent–rock interface. For high-frequency disturbance (150–200 Hz), vibration waves decay rapidly, only inducing local microcracks and limited debonding, and cannot form penetrating structural damage [39]. On this basis, the active excitation mode proposed in this study realizes controllable interfacial debonding by adjusting disturbance frequency: low frequency induces extensive interfacial softening, while high frequency achieves accurate local debonding. This provides a novel technical method for the recycling of abandoned anchor cables.

### 3.5. Analysis of pull-out load and energy response under different anchorage lengths

A 600-s vibration load was applied to specimens with anchorage lengths of 300 mm and 600 mm, using a 50 Hz sinusoidal excitation frequency. The energy response law of the anchor cable free section under different anchorage lengths was analyzed by comparing the post-vibration pull-out load attenuation degree of the specimens and the time-domain waveform characteristics monitored by acceleration sensors on the anchor cable free section and steel tube surface [40].

After excitation with 50 Hz sinusoidal load, the pull-out load-displacement curves of specimens with anchorage lengths of 300 mm (Group D50) and 600 mm (Group M50) are illustrated in Fig 17. As Group D50 has been elaborated above, only the deformation stages of M50 specimens are marked in the figure. The pull-out curves of Group M50 present distinct morphological differences: M50-1 shows a unimodal feature, while M50-2 and M50-3 exhibit bimodal characteristics. After the slip deformation stage, the curves of all specimens tend to be consistent, and all samples fail in the bonding failure and friction resistance reduction mode. The loading process can be divided into four stages: the elastic deformation stage within a normalized displacement (*S/La*) of 0–0.022, the elastic-slip transition stage at 0.022–0.121, the slip deformation stage at 0.121–0.331 with a rapid rise in pull-out load, and the complete interfacial debonding stage when the normalized displacement exceeds 0.331, eventually resulting in overall debonding failure of the anchor cable. The peak pull-out loads of M50-1, M50-2 and M50-3 are 226.94 kN, 213.87 kN and 224.96 kN, with an average peak strength of 221.91 kN, which is 156.60% higher than the average value of 86.48 kN for Group D50 (300 mm anchorage length). In contrast, Group D50 presents typical bimodal curves, with obvious bearing capacity attenuation and severe damage after vibration. Comparative analysis verifies that increasing anchorage length can effectively extend the elastic deformation stage, enhance peak pull-out strength and reduce the attenuation of bearing capacity. The internal mechanism is that a longer anchorage length increases the bonding area between anchor cables and surrounding rock, improves the overall stiffness of the anchorage system, and strengthens the energy dissipation capacity at the cable-anchoring agent-rock interface. Accordingly, the anchorage structure gains better resistance to dynamic disturbance [41].

**Fig 17:**
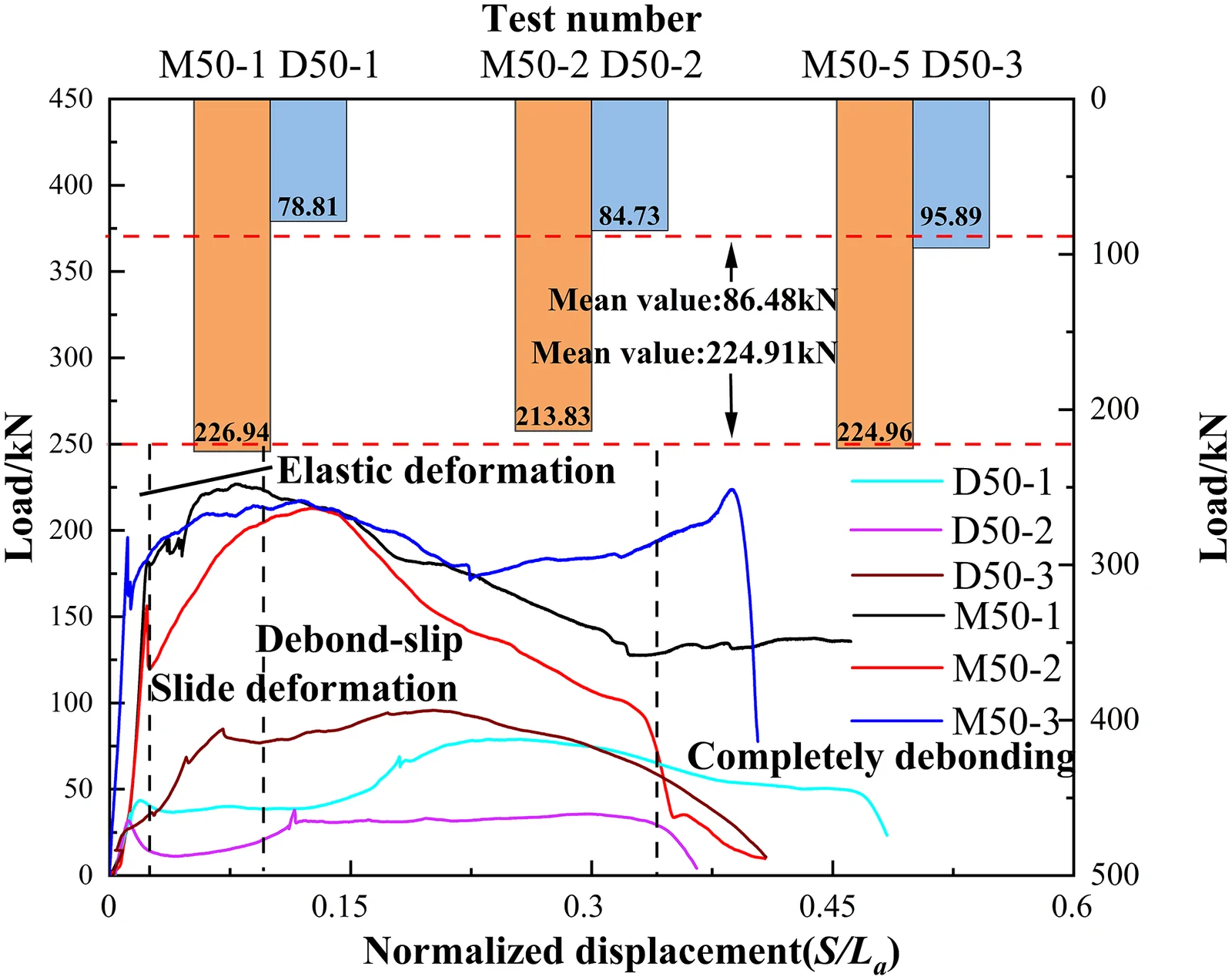
Comparison of normalized pull-out load-displacement curves of specimens with anchorage lengths of 300 mm and 600 mm.

Adopting the previous analytical framework, the full-duration time-domain signals were first converted to a dimensionless scale by normalizing the absolute measurement time against the total 600-s excitation duration (yielding a normalized time *t*/*T* ranging from 0 to 1.0). Subsequently, the signal segments collected during the stable vibration stage were processed for frequency-domain analysis via the Fourier transform. Comparative results are presented in Fig 18. The acceleration amplitude at the middle of the steel pipe (Channel 2) reaches a minimum. This phenomenon is primarily attributed to stress wave damping attenuation and the superposition of boundary reflections from both ends during longitudinal wave propagation, potentially causing the middle section to act as a vibration node. As the anchorage length increased from 300 mm to 600 mm, the frequency-domain peak acceleration of the anchor cable dropped from 5.572 g to 0.0381 g, reflecting an amplitude attenuation rate of 99.32%. Regarding energy transfer and dissipation mechanisms, the energy accumulation effect in the free segment is significantly weakened with the growth of anchorage length. Increasing the anchorage length effectively enhances the overall constraint stiffness of the surrounding rock–anchor cable system, restrains axial and transverse cable deformation, mitigates structural resonance under dynamic loading, and fundamentally reduces the dynamic response intensity of the anchorage body. Furthermore, a longer anchorage length expands the bonding contact interface between the anchor cable and surrounding rock. Vibration waves generated by dynamic disturbance produce continuous refraction and scattering at multi-layer medium interfaces. Substantial wave energy is dissipated at these anchorage interfaces, which greatly weakens the longitudinal transmission efficiency of vibration energy along the anchor cable.

**Fig 18:**
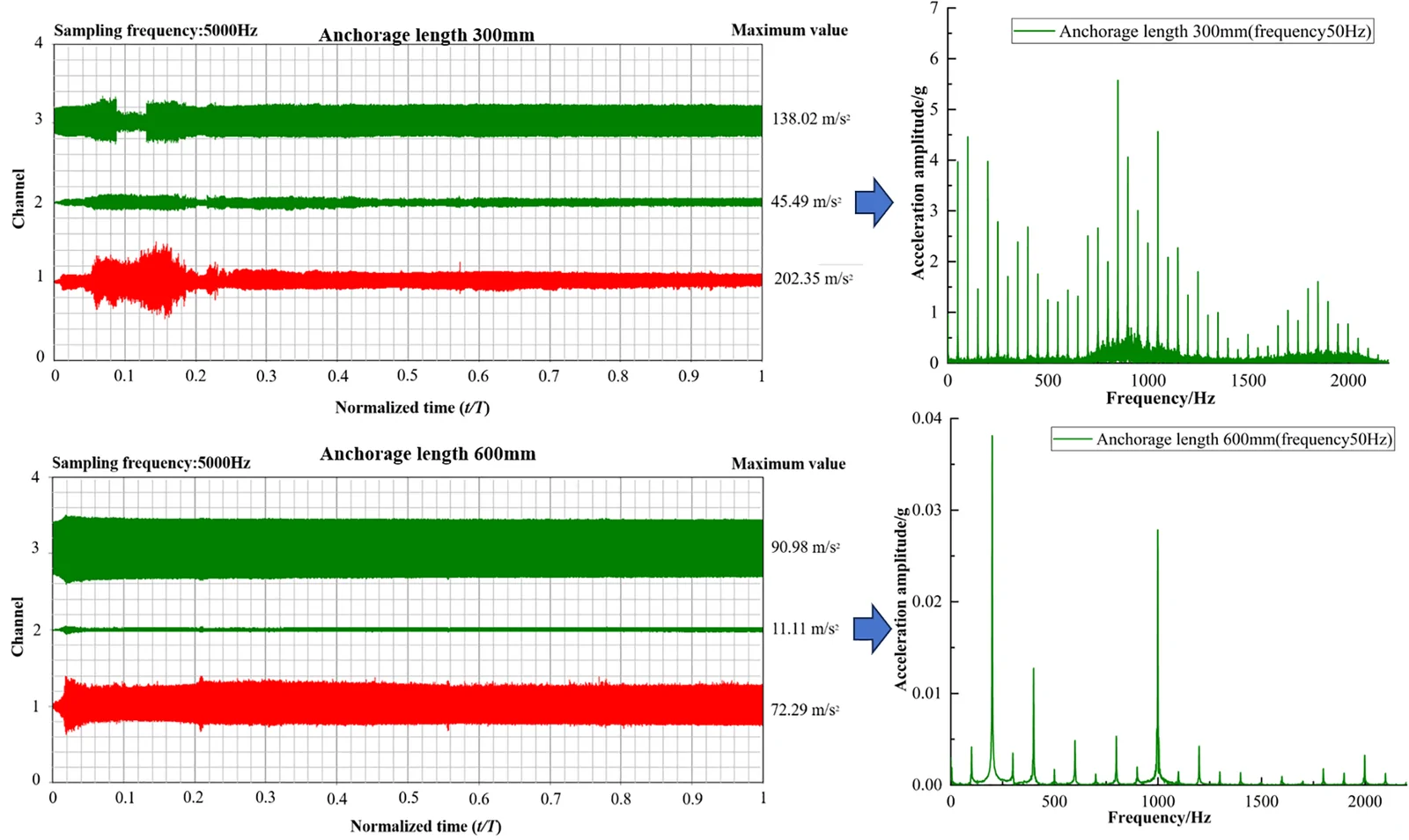
Comparison of normalized time-domain and frequency-domain waveforms of specimens with 300 mm and 600 mm anchorage lengths.

In essence, increasing the anchorage length can simultaneously optimize the stiffness characteristics of the anchorage system, narrow the disturbance response range of the anchor cable free segment, and strengthen the interfacial wave energy dissipation effect. Under the coupling of multiple factors, the dynamic vibration of the free segment is significantly restrained. Combined with the pull-out test data after 50 Hz sinusoidal dynamic excitation, the damage degree of the anchorage structure induced by dynamic load decreases from 38.74% to 7.53% with the increase of anchorage length, which effectively alleviates interfacial deterioration and structural damage. The frequency-domain analysis further verifies that a longer anchorage length leads to a substantial reduction in the acceleration amplitude of the anchor cable free segment. External disturbance energy is difficult to accumulate in the free segment, thereby greatly reducing the progressive failure risk of mine anchorage support structures under dynamic disturbance.

## 4. Simulation on internal damage evolution of anchorage structure

### 4.1. Model parameters

To clarify the evolution law of the internal fracture field during disturbance, numerical simulation was adopted for supplementary analysis. Given the unique advantages of the discrete element method in investigating anchorage body failure and crack propagation, the PFC^2D^ software was employed to explore the internal damage evolution of the anchorage body [42], and the corresponding numerical model was established as presented in Fig 19. The size of the anchorage body model is 77 mm × 800 mm. The anchor cable has a diameter of 21.8 mm and a total length of 1200 mm, with a particle diameter of 4.36 mm and a borehole diameter of 30 mm. The thickness of the anchoring agent on both sides of the anchor cable is 4.1 mm. For both the anchoring agent and the rock matrix, the particle diameter is controlled within the range of 0.4–0.8 mm, and the model porosity is set to 0.15.

**Fig 19:**
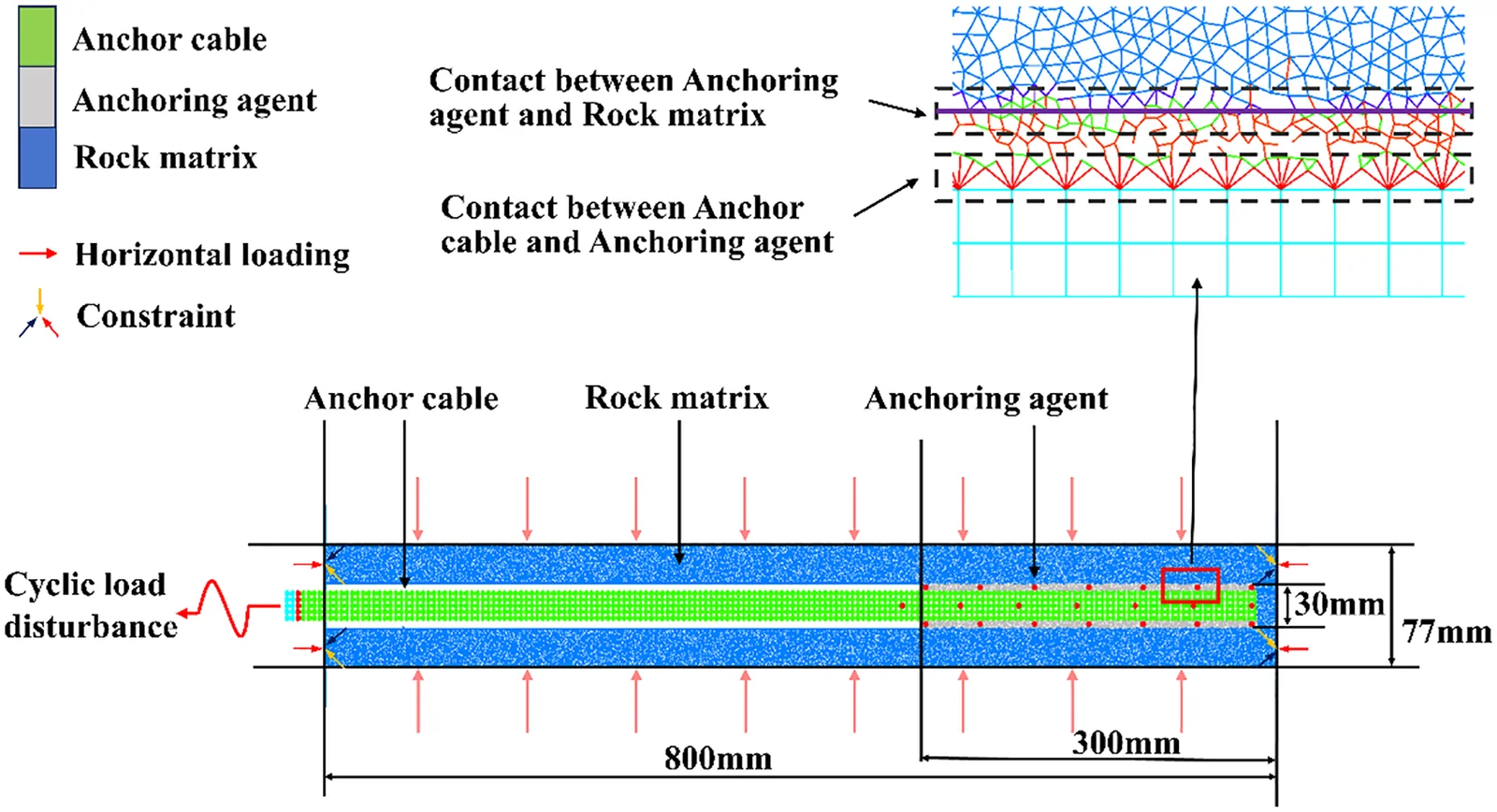
The establishment of a numerical model.

Utilizing the fundamental mechanical properties of the anchoring agent and host matrix derived from physical experiments [43], an iterative calibration procedure was executed to ascertain the corresponding micro-mechanical inputs for the computational model [44]. Through rigorous comparative analysis between empirical data and simulated outputs, these mesoscopic variables were finely tuned until the macroscopic mechanical response of the synthetic model accurately mirrored that of the physical specimens. Concurrently, the specific variables governing the simulated cyclic excitations were firmly validated. The finalized micro-parameters defining the anchorage medium are comprehensively detailed in Table 2.

**Table 2:** Mesoscopic parameters of the numerical model.

Mesoscopic parameters	Rock matrix	Anchoring agent	Anchor cable
**Particle density /kg⋅m** ^**-3**^	1900	2700	7950
**Linear contact modulus /GPa**	2	2	200
**Linear contact stiffness ratio**	1.2	1.2	1.5
**Parallel bond modulus /GPa**	1	1	200
**Parallel bond tensile strength /MPa**	2	1.8	1860
**Binding strength /MPa**	16.5	17	1860
**Coefficient of friction**	0.5	0.5	0.5

To probe the progressive damage mechanisms within the anchored mass, a sinusoidal tensile excitation was introduced at the upper terminal of the cable. Customized subroutines, scripted via the native FISH interface in PFC^2D^, were deployed to computationally track both the spatial propagation and the quantitative accumulation of internal fractures throughout the loading sequence. Fig 16 illustrates the designated oscillatory loading trajectory. To replicate the constant-amplitude sinusoidal disturbance utilized in the physical experiments, this specific waveform was integrated directly into the numerical servo-control mechanism. The target excitation is mathematically governed by the following expression:


$$\begin{array}{c} F=Asin\left(2\pi fT\right)+B \end{array}$$ (6)

Where: *F* is the applied cyclic load, *A* is the loading amplitude, *B* is the vibration load, *f* is the disturbance frequency, and *T* is time.

### 4.2. Evolution law of fracture field

To reveal the differences in anchorage failure modes under different disturbance frequencies, fracture evolution characteristics at key time nodes of *T*/5, 2*T*/5, 3*T*/5, 4*T*/5 and T were analyzed, where *T* is the total duration of cyclic loading disturbance. The internal fracture evolution of anchorage bodies was investigated at disturbance frequencies of 10 Hz, 50 Hz, 100 Hz, 150 Hz and 200 Hz. As illustrated in Figs 20–24, at *T*/5, internal cracks initiate from the top of the anchorage body and form a small number of discontinuous fractures. Under the disturbance of 10 Hz, cracks propagate toward the bottom of the anchorage hole and extend radially outward. At 2*T*/5, cracks develop downward along the anchoring agent-matrix interface with intensified radial expansion. Tensile failure emerges at the anchorage end near the hole bottom, and radial cracks appear inside the matrix. At 3*T*/5, the quantity of cracks in the anchorage layer and surrounding matrix increases greatly, and microcracks germinate and expand outward at the top of the interface. The bottom interfacial debonding is significantly advanced under 10 Hz disturbance. At 4*T*/5, the cracking area of the anchorage matrix expands continuously, radial cracks extend deep into the matrix, and bond slip occurs between anchor cables and anchoring agents. At *T*, the anchoring agent-matrix interface at the anchorage hole bottom is completely debonded. The anchoring agent in the bonding section undergoes tensile failure and connects with the bottom debonded zone, ultimately resulting in the overall pull-out failure of anchoring agents and anchor cables.

**Fig 20:**
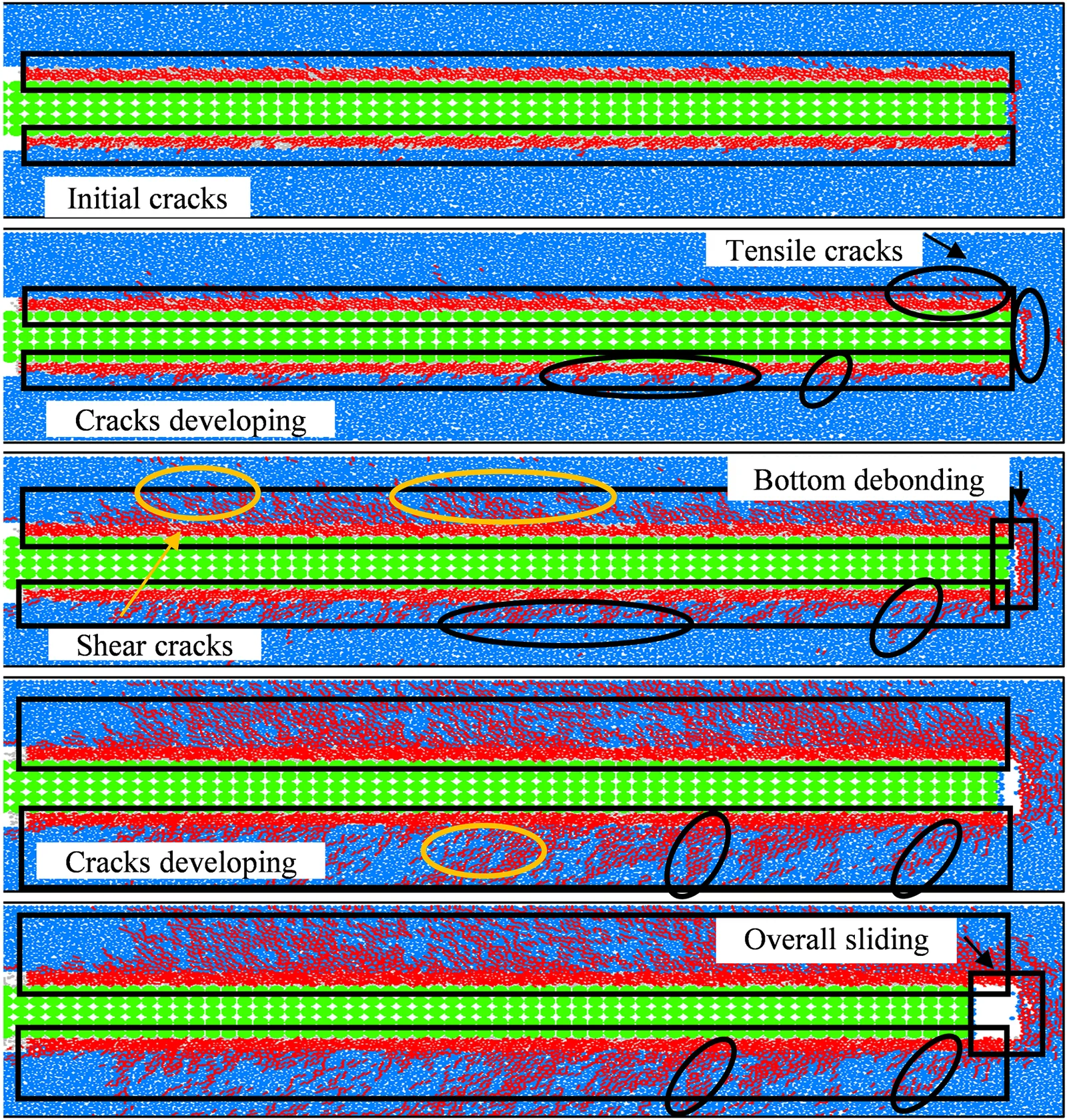
Crack field evolution of anchorage body at 10 Hz.

**Fig 21:**
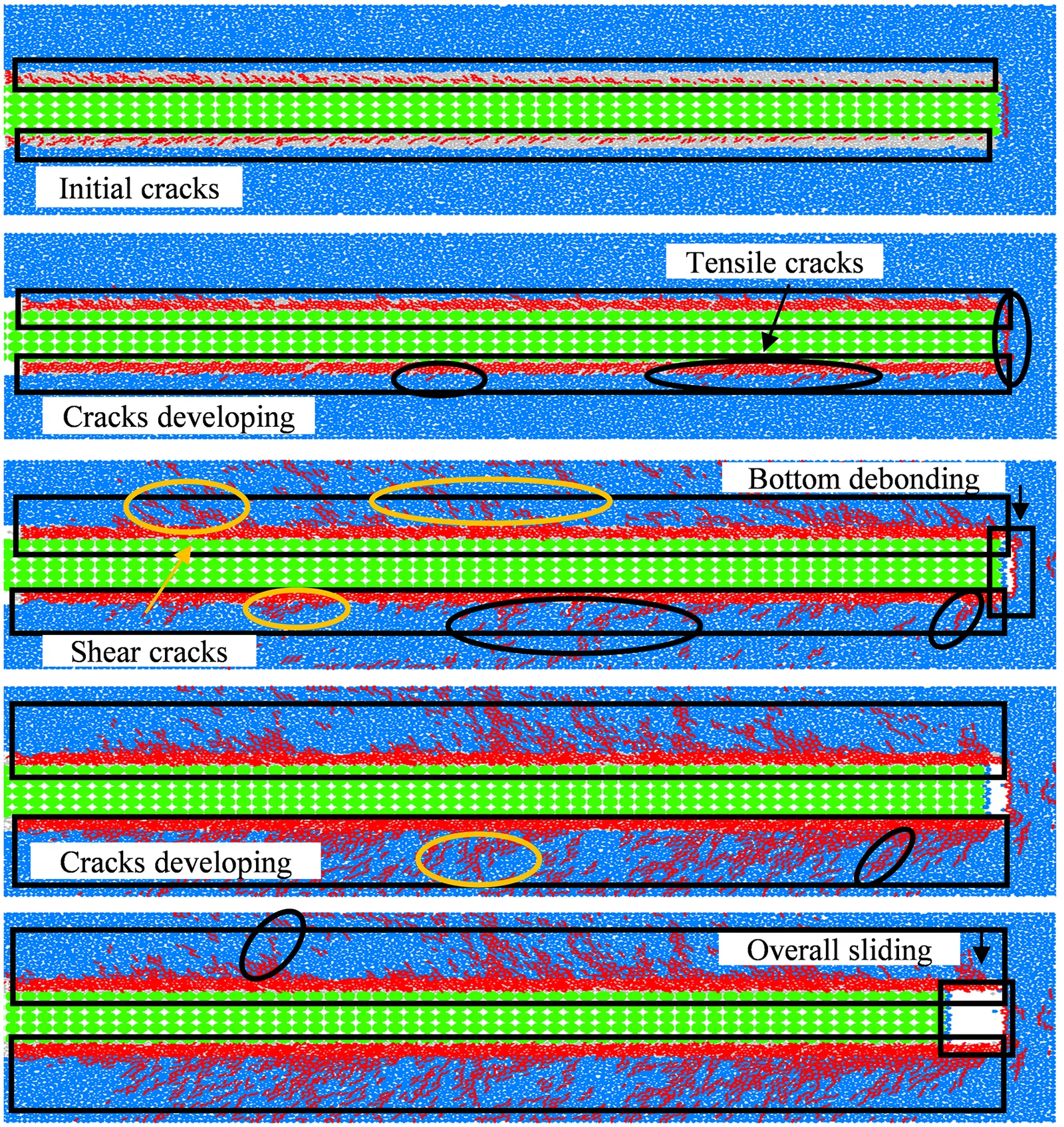
Crack field evolution of anchorage body at 50 Hz.

**Fig 22:**
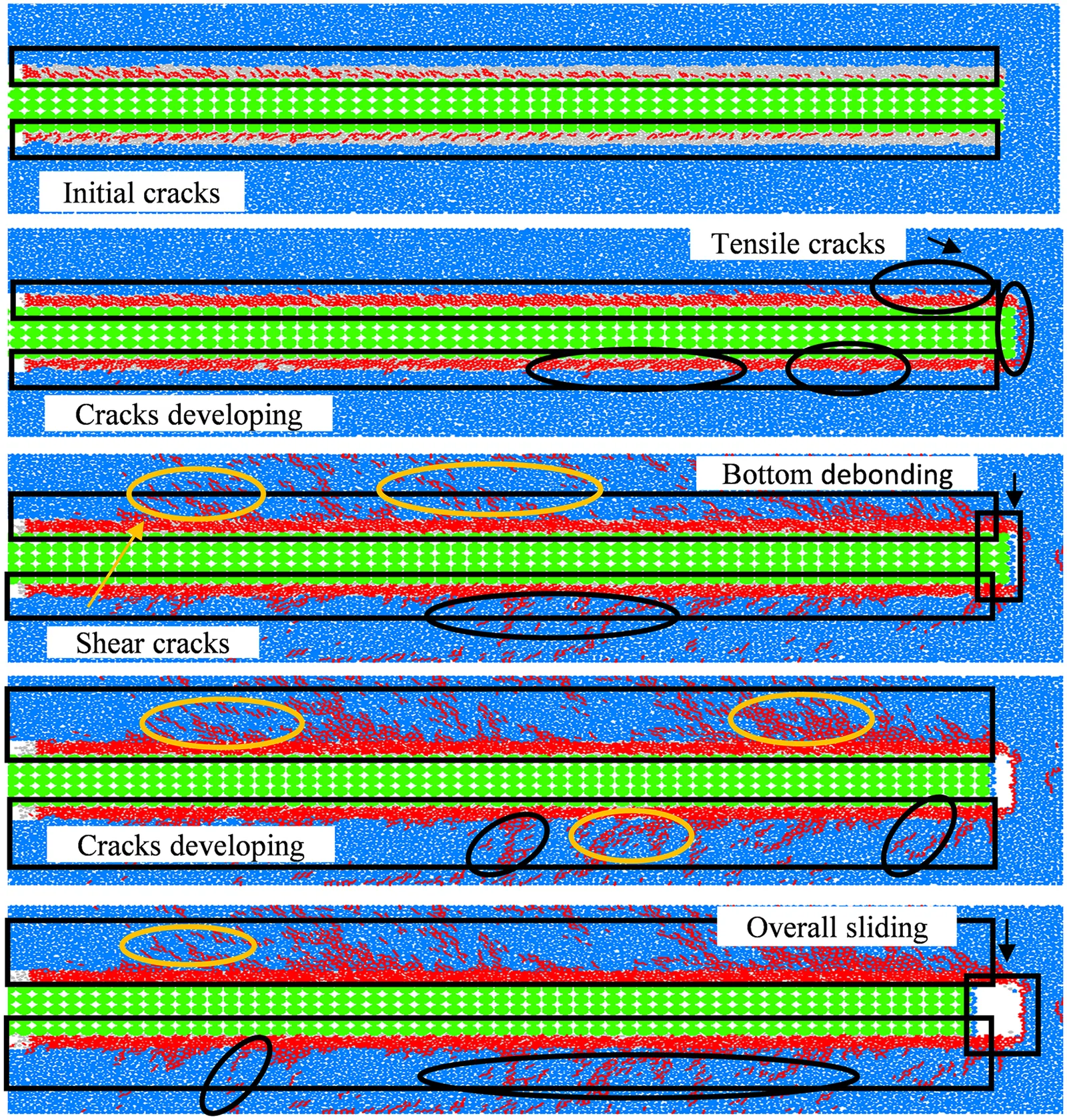
Crack field evolution of anchorage body at 100 Hz.

**Fig 23:**
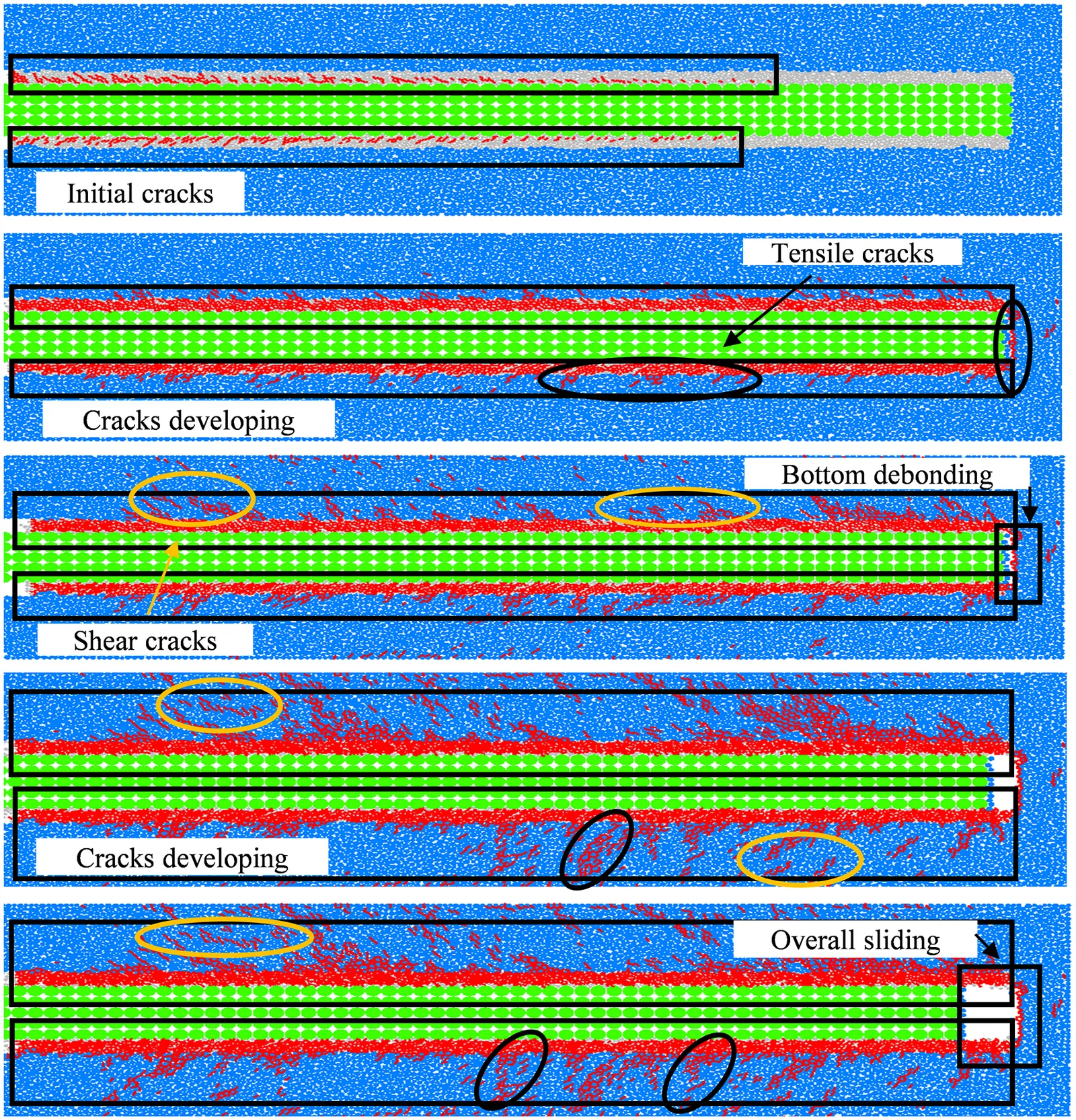
Crack field evolution of anchorage body at 150 Hz.

**Fig 24:**
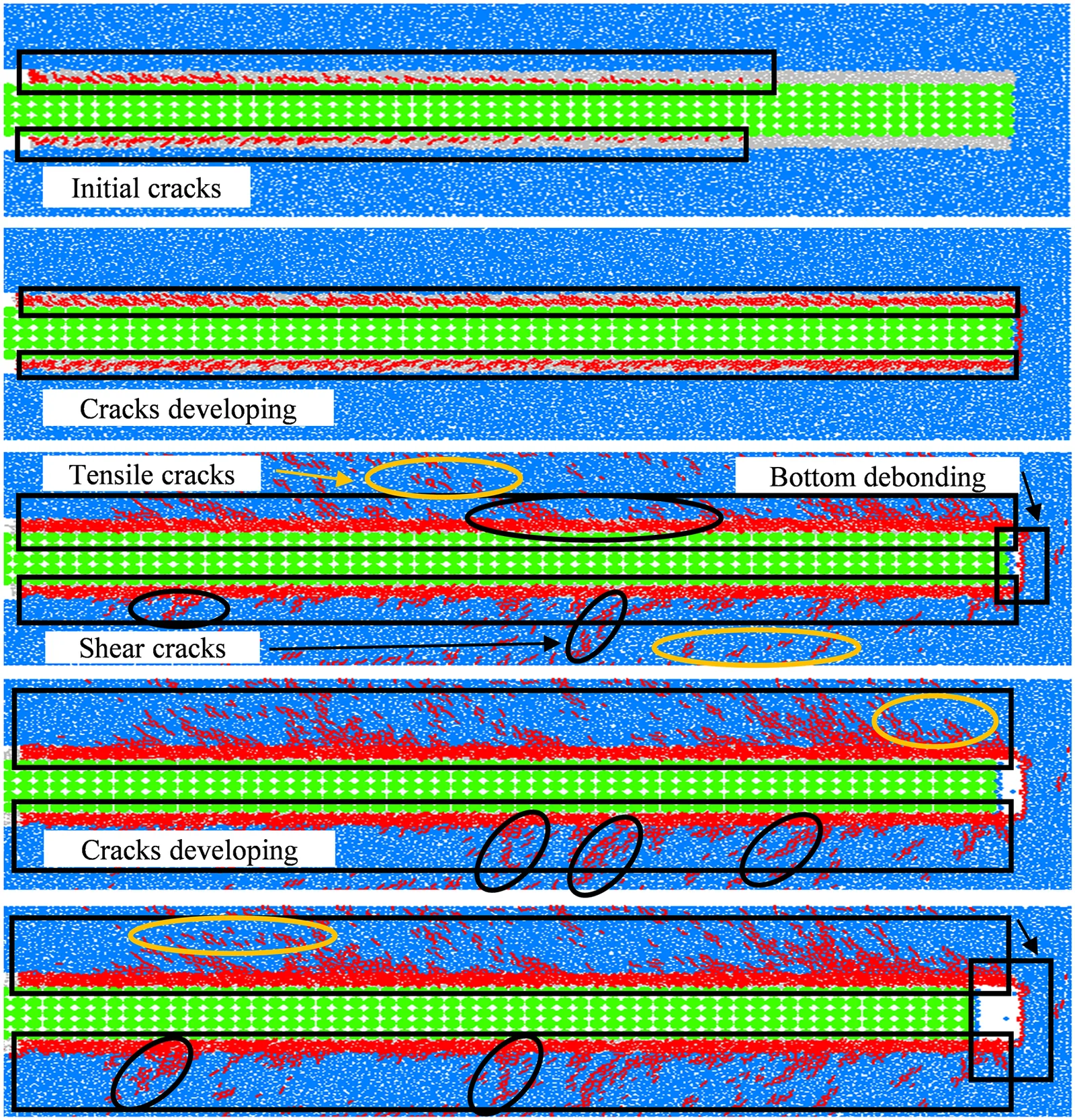
Crack field evolution of anchorage body at 200 Hz.

Subjected to varying disturbance frequencies and vibration loads, axial debonding and subsequent slip failure of the anchor cable occur at moment *T*. During this slip stage at 10 Hz, the end anchoring agent experiences initial damage and spalling. Radial and oblique cracks outside the anchoring agent propagate to form a continuous damage zone of measurable thickness. Meanwhile, secondary radial and oblique fissures develop within the adjacent matrix, with broader cracks concentrating at the cable base. This progressive crack expansion ultimately causes tensile failure across the anchoring agent ring. Laboratory tests reveal that during post-disturbance static pull-out, critical interfacial damage leads to the simultaneous extraction of the cable and the anchoring agent. Concurrently, basal delamination and tensile failure manifest at the anchoring agent-matrix boundary, allowing cracks to fully penetrate the matrix.

Compared to the 10 Hz regime, disturbance frequencies ranging from 50 Hz to 200 Hz significantly suppress both the radial and oblique fracture propagation within the anchoring agent ring, while also mitigating interfacial detachment at the base of the anchoring agent-matrix boundary. Despite these higher frequency effects, tensile rupture persists as the governing failure mechanism for the anchoring agent. It is observed that the total damaged area exhibits a monotonic decline as the excitation frequency increases. Under these medium-to-high frequency scenarios, a finite fractured zone remains detectable within the anchoring agent. Furthermore, both the radial and oblique cracking extents are markedly reduced relative to the 10 Hz condition. However, the rapid attenuation of these medium-to-high frequency stress waves triggers intense localized energy dissipation. Consequently, this phenomenon distinctly leads to increased fracturing within the surrounding rock adjacent to the anchoring agent, forming a complex secondary micro-crack network. Distinct from 10 Hz and 200 Hz, cracks are mainly concentrated at the anchoring agent–matrix interface, and radial cracks intermittently propagate from the anchoring agent to the matrix. As the tensile load increases, the internal damage area of the anchorage body expands, and the radial cracks outside the anchoring agent extend steadily to form a continuous damage zone with a certain thickness. Afterwards, penetrating cracks develop in the upper and middle regions of the anchorage matrix and extend outward. The through-cracks in the upper matrix ultimately result in splitting failure. The fracture evolution of the anchorage body under dynamic disturbance consists of four stages: initial damage, crack propagation, damage accumulation and instability failure. Internal fractures originate from the anchor cable–anchoring agent interface and gradually expand toward the outer anchorage matrix.

### 4.3. Evolution characteristics of internal crack quantity of anchorage body

By analyzing the internal fracture evolution of the anchorage body from the initiation of cyclic load disturbance to interfacial debonding failure, the variation trend of damage degree was inversely deduced [45]. Taking the total crack number, tensile crack number, and shear crack number as the vertical coordinates, and the computational loading steps as the horizontal coordinate, the variation curves were plotted (Figs 25–27). It is crucial to note that the computational loading steps essentially serve as a theoretical proxy for the actual vibration duration. Therefore, these curves explicitly illustrate how cumulative anchorage damage non-linearly couples with loading time.

**Fig 25:**
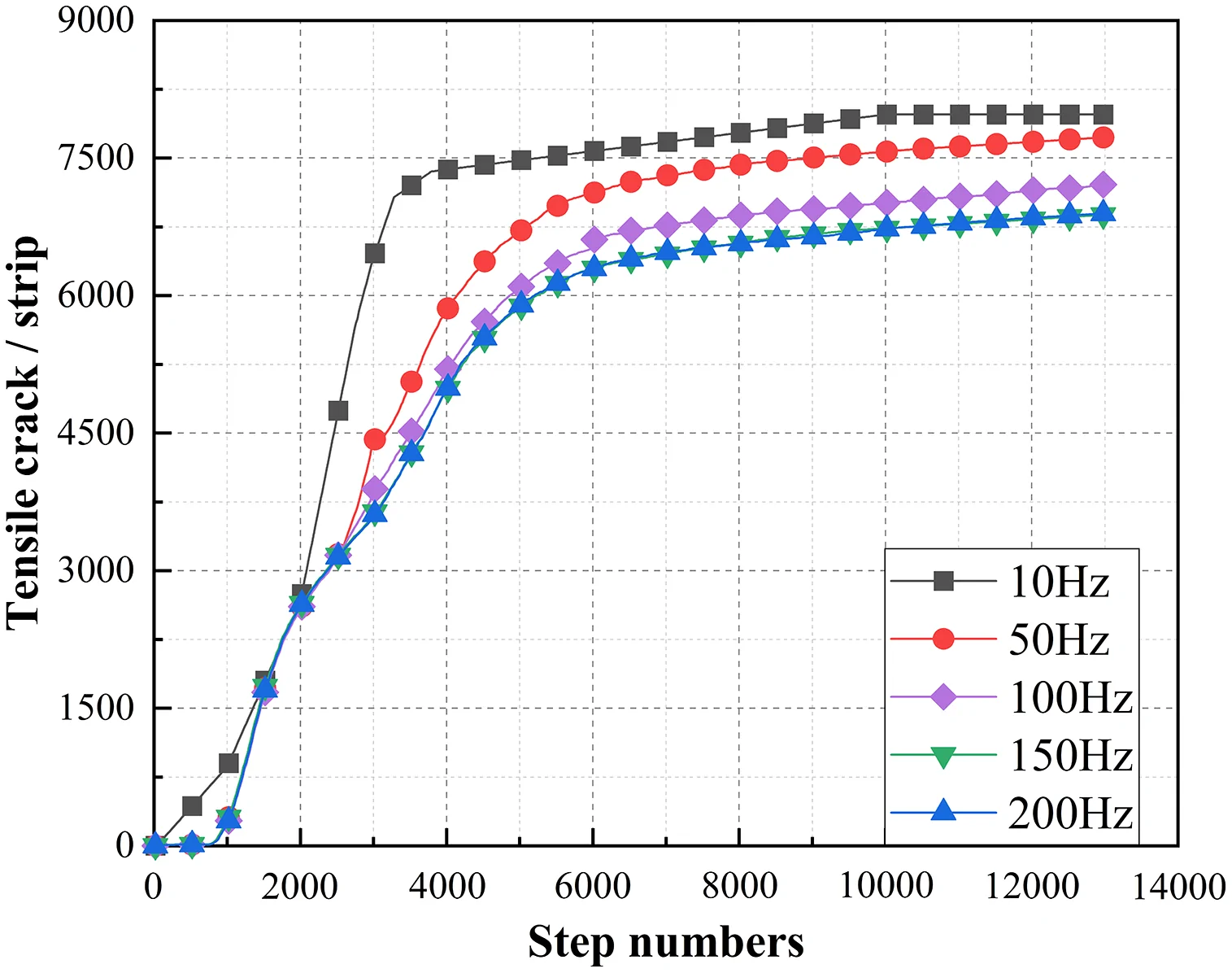
Tension crack.

**Fig 26:**
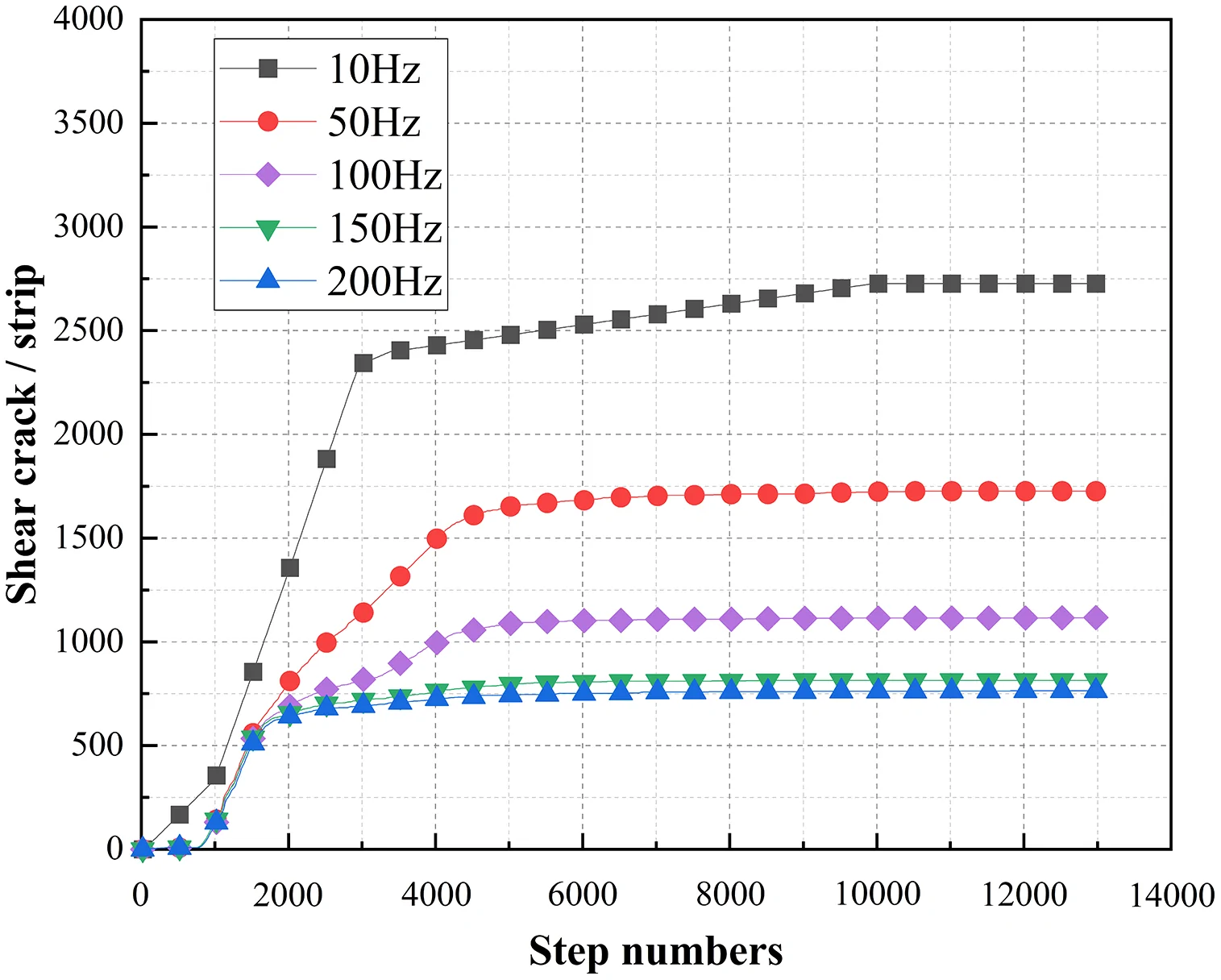
Shear crack.

**Fig 27:**
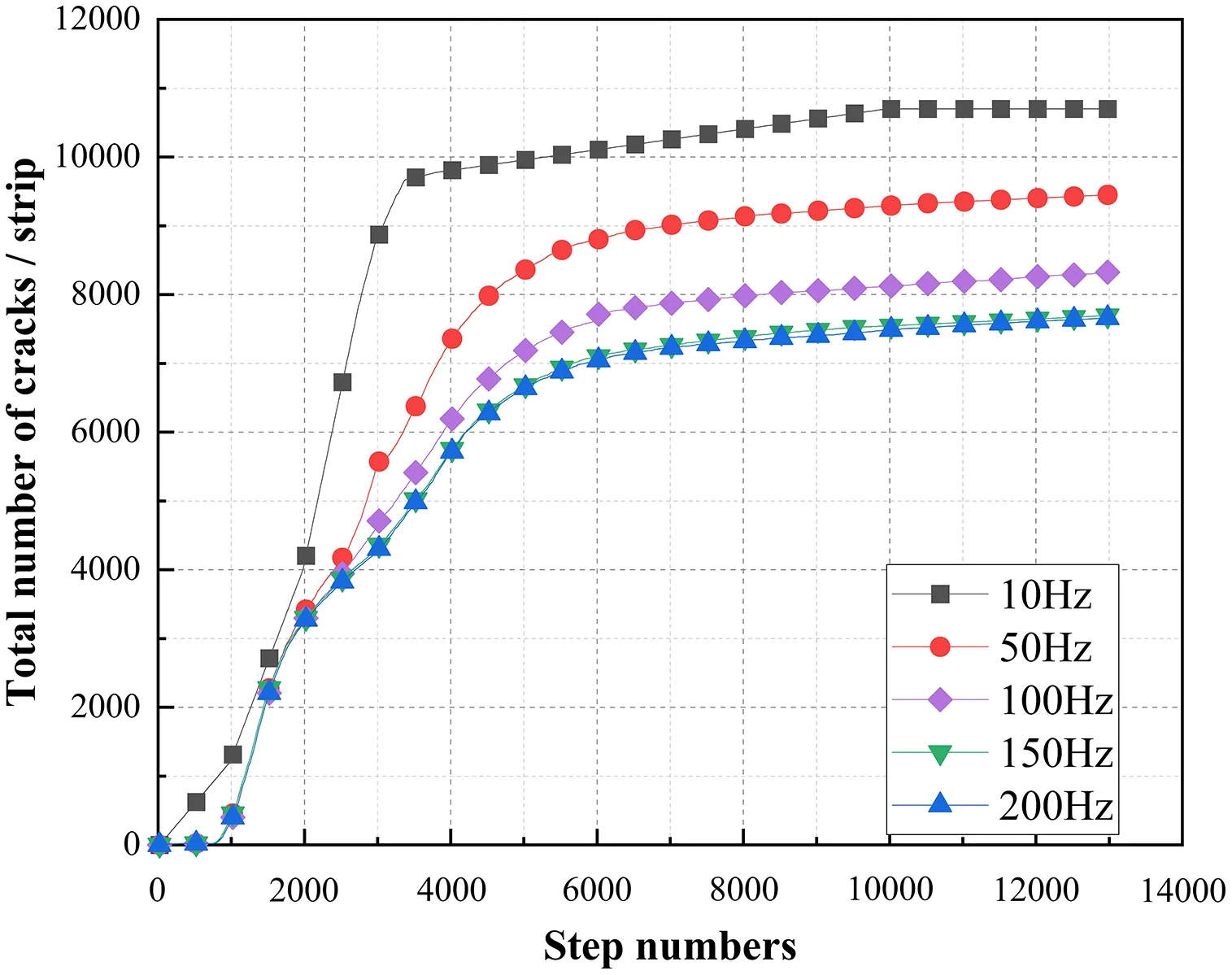
Total number of cracks.

Across the experimental frequency range of 10–200 Hz, the tensile crack ratios are 74.51%, 81.74%, 87.00%, 89.00%, and 90.00%, respectively. The clear prevalence of tensile fractures at every tested frequency suggests that tensile-induced degradation governs the internal failure mechanisms of the anchorage body. Furthermore, the population trends for all three fracture categories follow a characteristic convex-upward trajectory. As dynamic excitation continues, the fracture accumulation rate exhibits a marked deceleration, eventually approaching a state of quasi-equilibrium. Synthesizing the observed bearing capacity degradation and fracture evolution behaviors under cyclic loading, lower frequency disturbances are shown to significantly exacerbate fracture propagation, thereby substantially compromising the load-carrying capacity of the anchorage structure.

## 5. Discussion

### 5.1. Damage and failure mechanism of anchorage body

Combining laboratory tests with numerical simulation, this study systematically investigates the bearing characteristics of anchorage bodies under dynamic load disturbance. In pull-out tests with different disturbance frequencies, the anchorage body presents obvious load amplitude dependence and progressive damage propagation characteristics. The mechanical mechanism can be summarized as follows: when stress waves propagate inside the anchorage system, they dynamically couple with the interfacial bonding strength and continuously degrade the interfacial bonding performance. This forms a chained deterioration process of energy input–damage accumulation–strength attenuation.

The anchored roadway is simplified as shown in Fig 28. Under a low pull-out load, limited energy is input into the anchorage system. The shear stress in the anchoring agent is below its yield threshold, causing only elastic or negligible micro-plastic deformation. The interfacial bonding remains stable, and the anchorage body is in the elastic deformation stage [46]. When the pull-out load exceeds the critical value, abrupt changes of wave impedance at the anchor cable–anchoring agent interface induce severe stress concentration. A large amount of kinetic energy is converted into shear deformation energy, enabling the anchoring agent near the interface to enter a plastic flow state and produce irreversible shear deformation [47]. With the continuous development of plastic deformation, macroscopic shear slip failure eventually occurs at the anchor cable–anchoring agent interface. The failure initiates at the surface layer of the anchorage section with the most significant stress concentration and gradually extends inward, showing a progressive deterioration characteristic from the surface to the interior. The dynamic competitive relationship between pull-out strength and interfacial bonding strength essentially reflects the staged evolution of the energy dissipation mechanism. At the initial loading stage, energy dissipation is dominated by the viscoplastic deformation of the anchoring agent [48]. As the pull-out load increases, interfacial microcracks propagate continuously and the equivalent bonding strength decreases steadily. Accordingly, the dominant energy dissipation mode shifts from bonding slip to contact friction. The static load pull-out load-displacement curves in Fig 9 and the simulated crack propagation results in Fig 20–24 directly verify the above mechanism.

**Fig 28:**
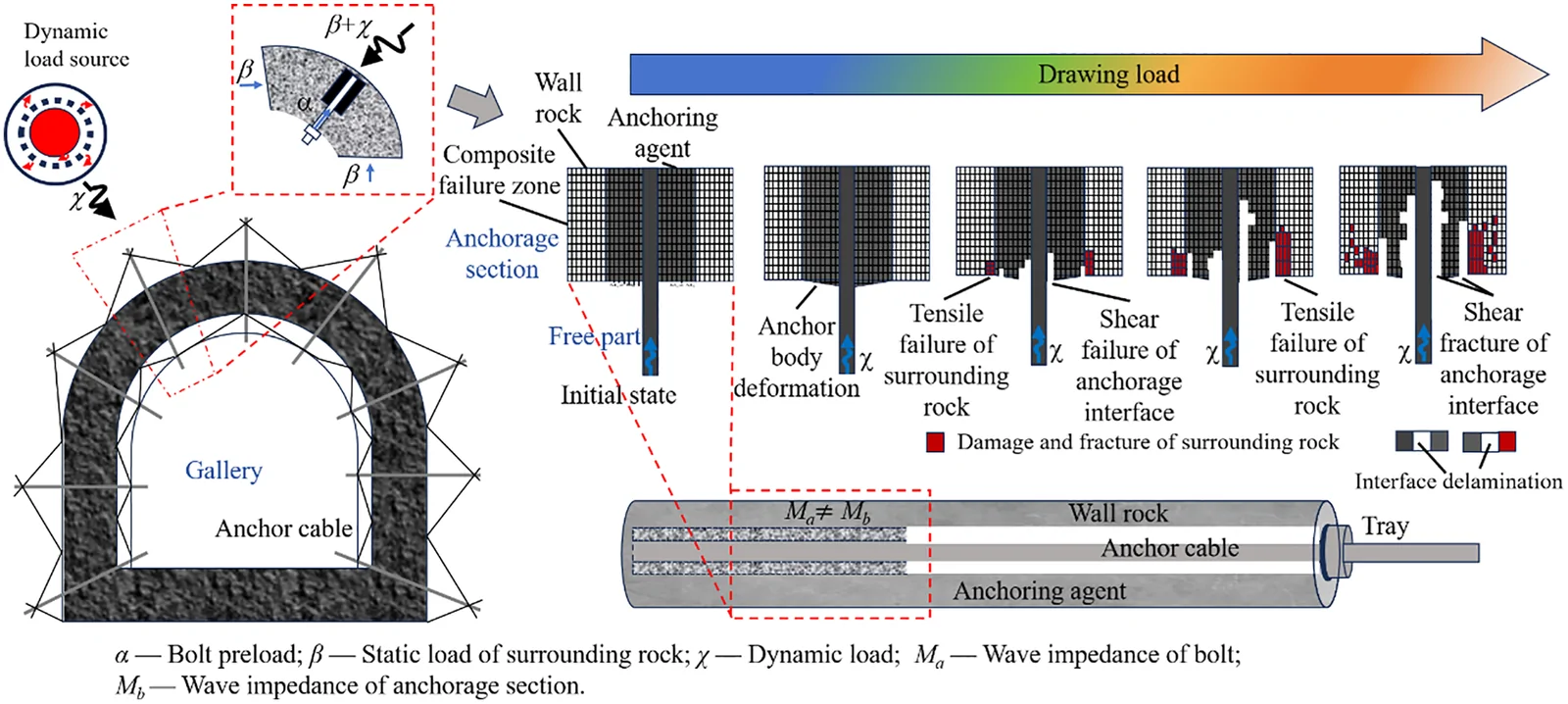
Progressive failure process of anchorage body under pull-out load.

Physically, the wave impedance mutation during stress wave propagation concentrates energy on the weak interfacial layer of the anchoring agent. The surrounding rock absorbs and reflects most stress wave energy, with no obvious overall deformation. Circumferential debonding and shear failure in the composite failure zone are directly caused by shear stress concentration induced by pull-out action. Significant differences in dynamic mechanical parameters such as elastic modulus and wave velocity between anchor cables and anchoring agents lead to incompatible deformation under stress waves, generating high-amplitude interfacial shear stress and driving the interface failure from micro-slip to macroscopic shear damage [49].

To explicitly elucidate the fundamental mechanics governing the tensile-shear composite failure under varying frequencies, a direct comparative analysis grounded in stress wave propagation and dynamic material responses is essential. Under low-frequency disturbances (e.g., 10 Hz): The dynamic loads generate long-wavelength stress waves that significantly exceed the effective anchorage length. This condition places the entire anchorage interface within a quasi-static regime characterized by continuous, reciprocating cyclic shear. Supported by the findings of Wu et al. [31] and Wang et al. [38], such low-frequency, long-duration cyclic loading facilitates continuous fatigue damage accumulation, effectively overcoming the interfacial shear resistance. Consequently, energy dissipation is globally distributed along the interface and is predominantly governed by macroscopic sliding friction. This mechanism drives a “progressive unzipping” debonding mode, manifesting as extensive, uniform shear slip.

Conversely, medium-high frequency excitations (50–200 Hz): These disturbances inherently generate short-wavelength stress waves coupled with elevated strain rates. As demonstrated by Wang et al. [30], the anchoring matrix exhibits a pronounced strain-rate hardening effect under high-frequency dynamic loading, prompting a transition from ductile shear yielding to brittle tensile fracturing. Mechanistically, these short-wavelength waves undergo intense reflection and scattering at the impedance-mismatched boundaries, resulting in severe “localized energy trapping” near the excitation source. Because the elevated strain rate restricts large-scale ductile shear slip—thereby inducing “localized shear locking”—the trapped dynamic energy violently forces the embrittled anchoring matrix to expand radially. Therefore, the dominant energy dissipation mechanism shifts from global friction to localized dynamic fracture energy, initiating a high density of tensile micro-cracks (the shattering effect). This directly explains why high-frequency disturbances inherently trigger localized tensile-splitting, whereas low-frequency loads favor global shear debonding.

### 5.2. Controllable debonding and full recovery mechanism of anchor cable

Experimental results demonstrate that disturbance frequency is the key parameter governing the degree, range, and pull-out resistance of interfacial debonding. Under 10 Hz low-frequency sinusoidal cyclic disturbance, the vibration features a longer wavelength, deeper energy transmission, and slower attenuation. Continuous reciprocating shear acts on the entire anchorage interface, progressively weakening the interfacial bonding strength and driving microcracks to fully propagate and coalesce. This forms progressive, large-scale, and axial debonding. Borne only by residual friction, the anchor cable can be completely extracted under a low pull-out force. This effectively prevents cable fracture, residual damage, and secondary deterioration of the surrounding rock, as shown in Fig 29.

**Fig 29:**
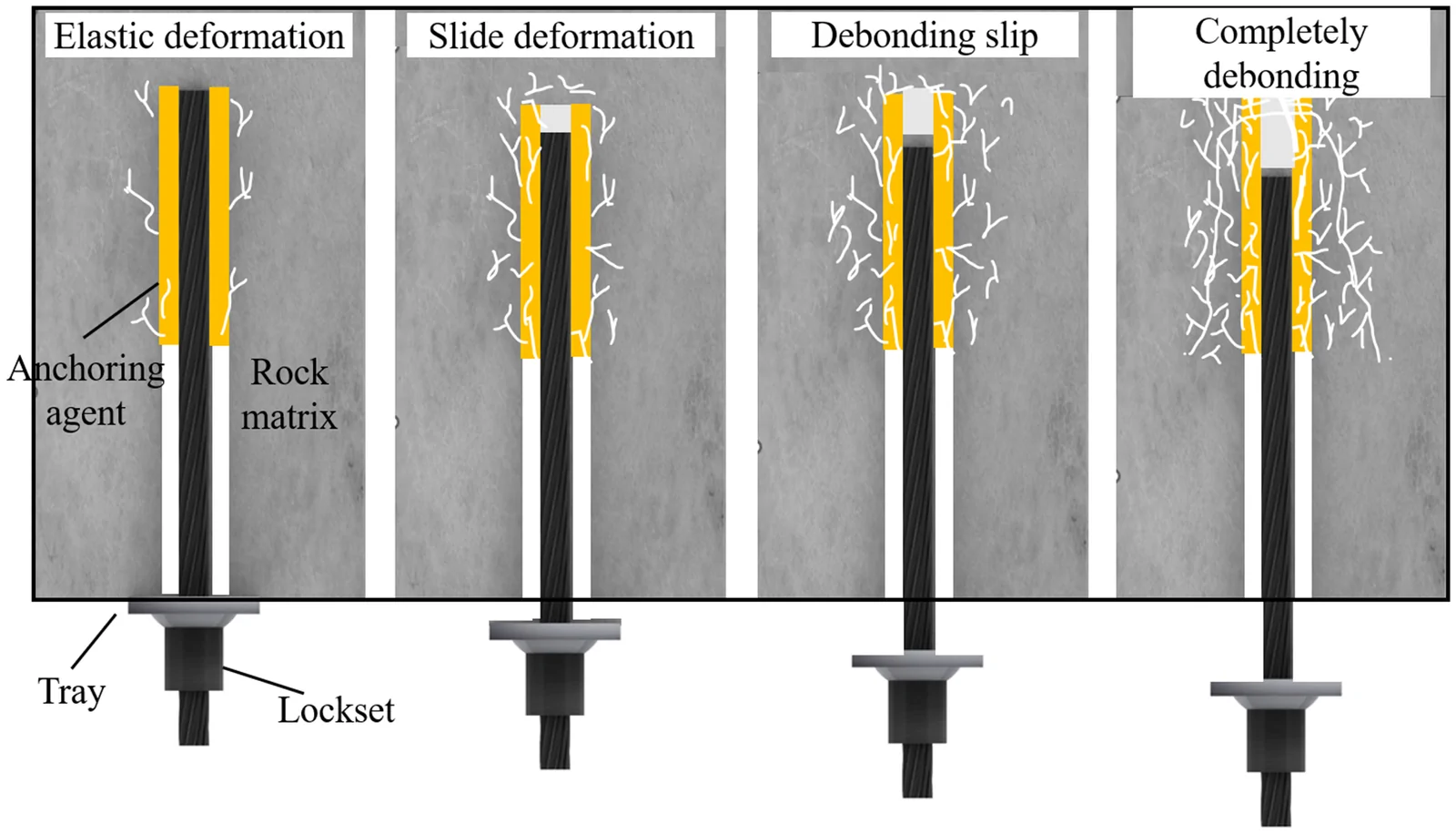
Crack evolution under low-frequency disturbance.

Medium- and high-frequency disturbances above 50 Hz feature short wavelengths and rapid attenuation. These dynamic characteristics heavily impede the large-scale longitudinal transmission of disturbance energy, causing severe energy concentration at localized interfaces. This localized energy dissipation inhibits long-distance axial debonding, producing only localized microcracking and short-distance debonding. Concurrently, substantial fatigue strain energy radiates into the adjacent surrounding rock, inducing the initiation and propagation of denser and more complex cracks within the anchorage body. These cracks mainly concentrate at the anchoring agent–surrounding rock interface, as shown in Fig 30, requiring a higher pull-out force for cable extraction.

**Fig 30:**
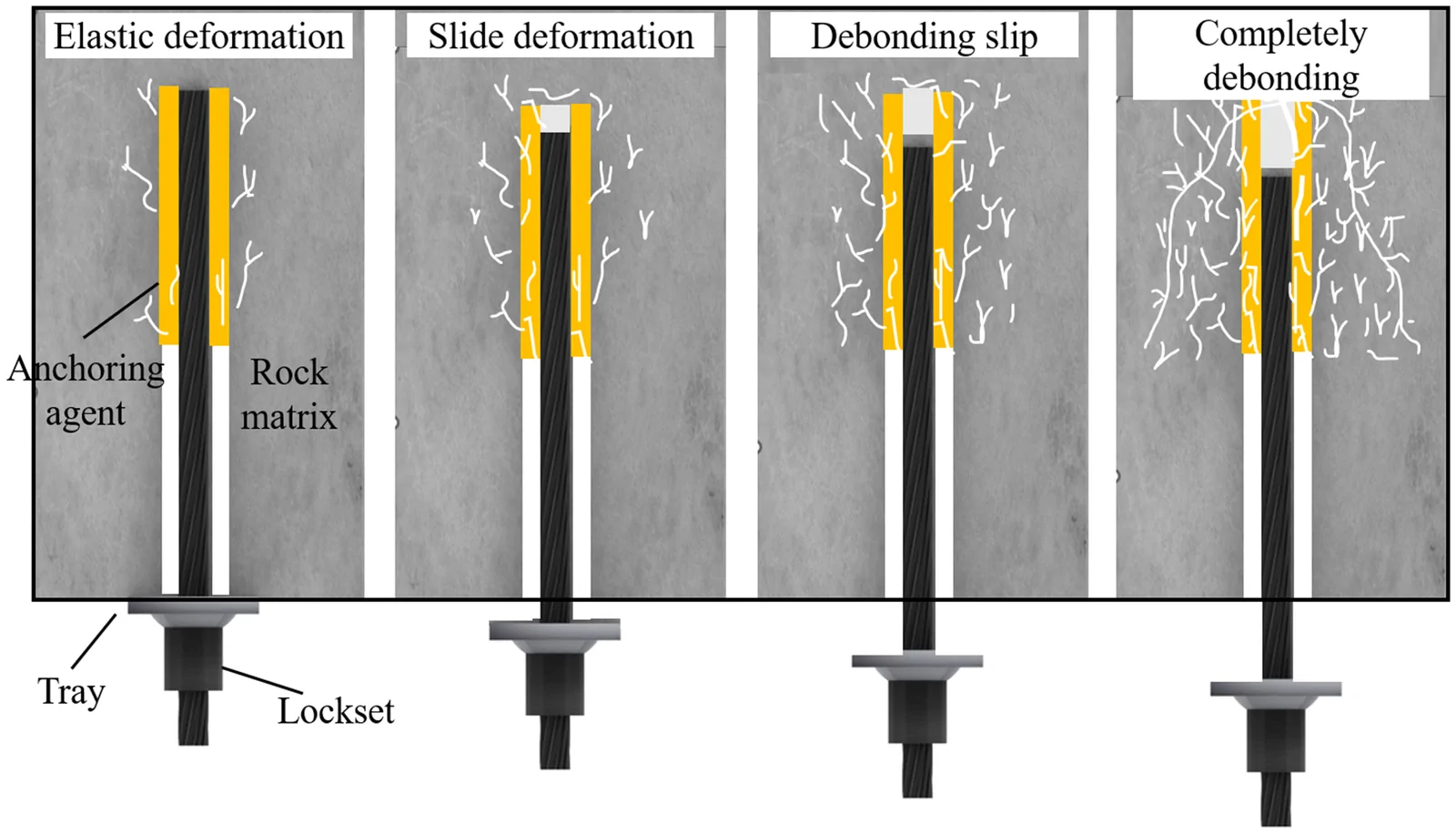
Crack evolution under medium-high frequency disturbance.

Low-frequency long-period disturbance provides stable energy input and produces cyclic shear at the anchor cable–anchoring agent interface, leading to large-scale degradation of bonding strength. The full-scale penetration of interfacial microcracks significantly reduces the interfacial friction coefficient and pull-out resistance. This guarantees desirable debonding performance without structural damage, ultimately realizing the low-resistance, high-efficiency, and controllable recovery of anchor cables.

Comparative tests of different anchorage lengths further verify that excessive anchorage length significantly enhances interfacial stability, restrains debonding behavior and hinders anchor cable recovery. A moderate anchorage length facilitates the transmission of low-frequency disturbance energy and extensive axial interfacial debonding, thereby improving the recovery efficiency and integrity of anchor cables. Combined with experimental results and mechanism analysis, the collaborative scheme of approximately 10 Hz low-frequency disturbance and reasonable anchorage length can realize large-scale axial debonding at the anchor cable–anchoring agent interface in a short time, which is the optimal working condition for safe, efficient and complete anchor cable recovery.

The proposed low-frequency disturbance method for controllable debonding and full recovery of anchor cables avoids destructive measures such as blasting and cutting. It presents prominent advantages including nondestructive performance, high efficiency, low cost and environmental friendliness. This research can provide theoretical support and technical reference for waste anchor cable recycling, support structure reuse, green mine construction and safe roadway support in underground engineering. However, it should be noted that the current controllable debonding mechanism is derived from laboratory-scale physical tests and numerical simulations under specific stress boundary conditions. In underground in-situ practices, the superimposed influences of high multidirectional in-situ stresses, lithological heterogeneity, and time-dependent rheological behaviors of the surrounding rock may complicate the axial debonding process. Future pilot-scale field investigations are essential to calibrate the optimal excitation parameters for varying geological conditions.

### 5.3. Experimental limitations and future perspectives

While this study systematically elucidates the frequency-dependent degradation mechanisms and proposes an active excitation method for anchor cable recovery, certain simplifications inherent in the physical models warrant acknowledgment.

First, regarding boundary conditions, the hollow steel pipes used in laboratory tests fundamentally differ from the semi-infinite medium of actual in-situ rock. The steel pipe provides a strong rigid radial constraint, which partially restricts the volumetric dilation and radial expansion of the anchorage matrix under dynamic loading. Furthermore, there is a significant wave impedance mismatch between the steel pipe boundary and the anchoring agent. In a true semi-infinite rock mass, dynamic stress waves would radiate outward and dissipate continuously. However, the high impedance of the rigid steel boundary causes the stress waves to reflect repeatedly within the container, which may artificially intensify localized energy accumulation and cause deviations in the measured absolute bearing capacity. Nevertheless, because macroscopic failure under pull-out loading is primarily dominated by axial interfacial shear debonding rather than radial matrix crushing, these radial boundary differences do not alter the fundamental failure mode. Consequently, the relative attenuation laws and frequency-dependent debonding mechanisms revealed in this study remain highly reliable. Future studies should utilize large-scale rock blocks or impedance-matched flexible boundaries to further validate the effects of multi-directional in-situ stresses on anchorage fatigue.

From a practical perspective, transitioning this recovery method to on-site applications introduces several practical challenges. First, underground anchorages endure long-term microseismic fatigue; the time-dependent rheological degradation of resin under years of cyclic loading remains to be quantified. Second, to achieve a decoupled mechanistic analysis, this study adopted constant-amplitude pure sinusoidal excitations. However, actual in-situ microseismic signals are considerably more complex, characterized by transient, variable-amplitude, and broad-spectrum features. The superposed damage effects of these complex multi-frequency disturbances remain to be thoroughly investigated. Third, while this study adopted symmetric axial excitation to decouple the frequency variable and establish a baseline mechanism, real-world underground dynamic disturbances (e.g., seismic waves or rock bursts) often impact at oblique angles, generating asymmetric eccentric excitations. Under such eccentric loading, the stress distribution along the anchorage interface becomes highly non-uniform. The induced bending moments and transverse shear forces will inevitably cause severe stress concentration on one side of the interface, potentially triggering premature localized crushing of the resin matrix and asymmetric debonding. This complex stress state would likely transition the idealized uniform axial debonding mechanism into a coupled bending-shear-tension failure, significantly accelerating the fatigue degradation compared to symmetric loading. Finally, implementing this technique requires specialized portable vibrators with safe, rigid clamping mechanisms to efficiently transmit low-frequency energy within confined roadway spaces. Addressing these long-term fatigue behaviors, complex waveform responses, asymmetric loading, and equipment designs is crucial for future engineering applications.

Furthermore, the current axial debonding mechanism is derived primarily from medium-hard sandstone. In deep practices, lithological heterogeneity significantly complicates this recovery process. First, in hard rock masses, significant impedance mismatch effectively traps disturbance energy at the interface, promoting highly efficient axial debonding. Second, soft rock may absorb vibration energy through global plastic deformation, requiring strictly minimized excitation energy and duration to prevent borehole collapse. Third, anisotropic layered rock masses scatter stress waves at bedding planes, inducing asymmetric shear slip and segmented interfacial debonding. Therefore, future pilot-scale field investigations under diverse geological conditions are essential to calibrate optimal excitation parameters for safe support structure reuse.

From a numerical perspective, while the PFC^2D^ simulation provides fundamental insights into interfacial dynamic responses, it inherently simplifies the cylindrical anchorage system into a planar model. This two-dimensional assumption restricts the accurate representation of three-dimensional spatial features, such as the spiral geometry of cable ribs, radial dilation-induced hoop stresses, and the spatial tortuosity of fracture networks. Consequently, the 2D model cannot fully capture the out-of-plane forces and complex spatial crack propagation. To overcome these inherent geometric limitations, subsequent research should employ 3D discrete element modeling (e.g., PFC^3D^) alongside full-scale physical vibration tests. These approaches will accurately replicate spatial stress distributions, capturing the true three-dimensional anchorage damage and interfacial debonding evolution under cyclic disturbances.

## 6. Conclusions

With the self-developed anchor cable anchorage and vibration test platform, sinusoidal dynamic excitation tests on end-anchored specimens were conducted. The pull-out strength attenuation and vibration energy response characteristics under different excitation frequencies and anchorage lengths were investigated, and the main conclusions are drawn as follows:

(1) This study confirms a negative correlation between disturbance frequency and bearing capacity attenuation of end-anchored specimens. Based on peak pull-out load data from static and post-excitation tests, low-frequency disturbance causes significant degradation: peak pull-out strength increases linearly with excitation frequency, while damage parameters decrease accordingly. Specifically, 10 Hz low-frequency disturbance induces the most severe damage (62.23% capacity attenuation), whereas 150–200 Hz high-frequency disturbance exerts minimal impact (5%–6% attenuation). STFT analysis further reveals that the free-segment vibration amplitude of anchor cables drops markedly with rising frequency. Low-frequency excitation enables long-distance longitudinal propagation and energy accumulation due to high transmission efficiency and slow attenuation. In contrast, high-frequency disturbance dissipates energy rapidly within a confined range, failing to form substantial energy accumulation.
(2) When subjected to uniform 50 Hz excitation under a constant force amplitude controlled by a constant input current, specimens with a 300 mm anchorage length exhibit a bearing capacity attenuation of 38.74%, compared to a mere 7.53% for those with a 600 mm anchorage length. Meanwhile, the latter delivers distinctly higher peak pull-out strength. It demonstrates that extending the anchorage length effectively mitigates vibration damage and improves the anti-disturbance performance of anchorage bodies. Moreover, longer anchorage improves structural stiffness and energy dissipation, greatly weakening the vibration response of anchor cable free segments.
(3) Numerical simulation results indicate a tensile-shear composite failure mode. In this process, interfacial debonding propagates axially along the anchor cable–anchoring agent interface, accompanied by tensile-dominated radial and oblique micro-cracks extending through the anchoring agent layer and the adjacent matrix. Lower disturbance frequencies generate an increased number of these cracks, thereby reducing the bearing strength of the anchorage structure.
(4) Clarified the dynamic damage mechanisms: wave impedance mismatch causes interfacial stress concentration, and energy dissipation shifts from viscoplasticity to contact friction. A 10 Hz disturbance with a moderate anchorage length induces full-interface axial debonding.

## Supporting information

S1 File This is·the Sl·raw images legend. (PDF)
