# Supplementary material for: Bearing capacity attenuation and energy response of end-anchored bodies under dynamic disturbance with different frequencies
Source: PLoS One. 2026 Oct 23;21(10):e0354419. doi: 10.1371/journal.pone.0354419 (PMC13693897; doi:10.1371/journal.pone.0354419)
Supplement: Supplementary file 1 [file pone.0354419.s001.pdf]

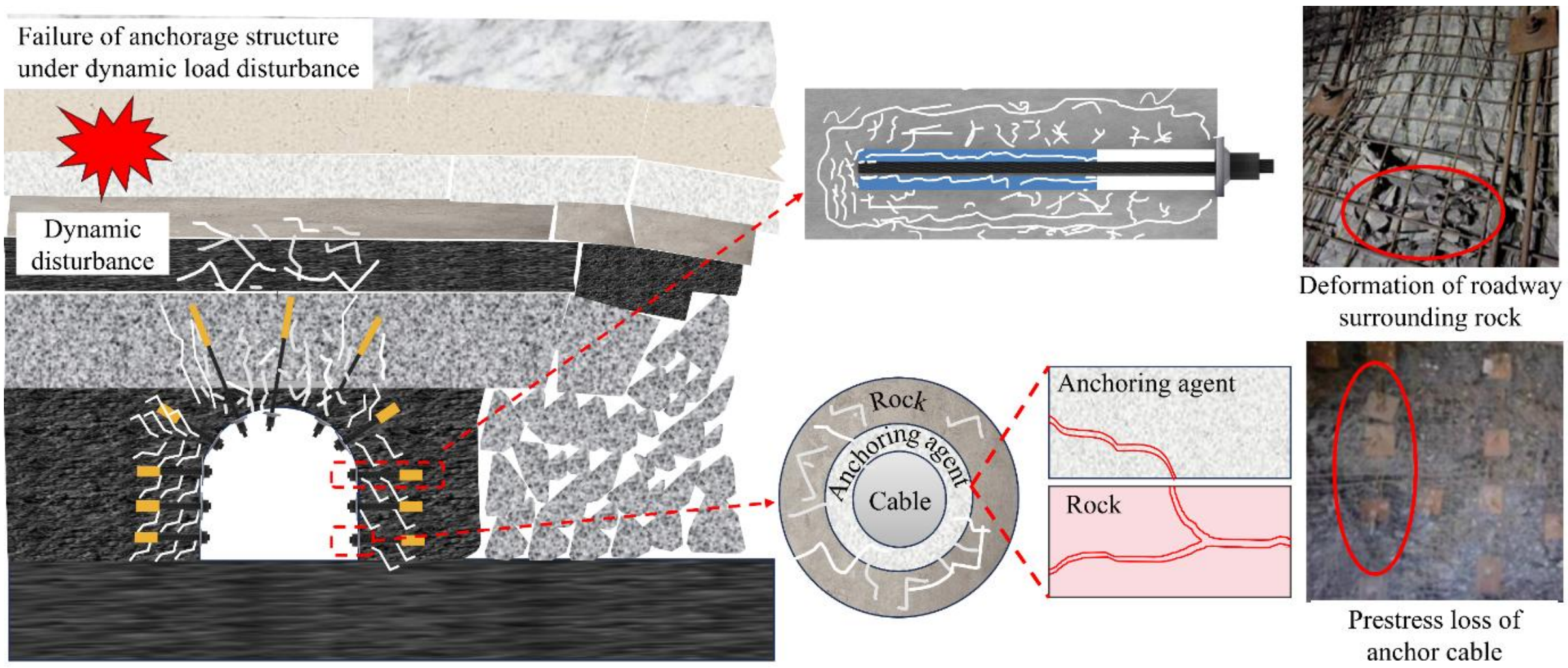

Fig 1. Schematic of anchor cable failure in the field

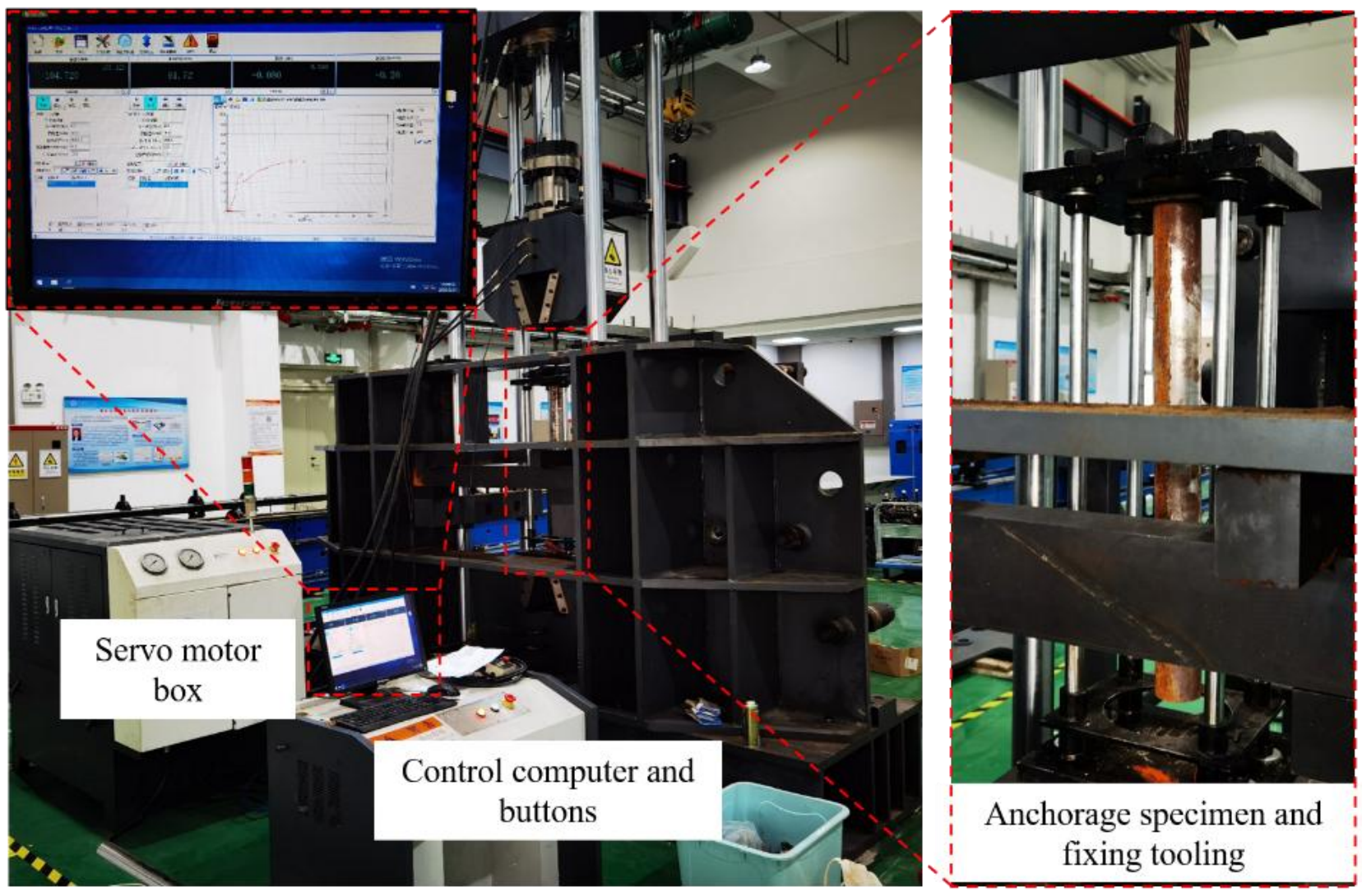

Fig 2. Comprehensive mechanical performance test apparatus for mine support materials

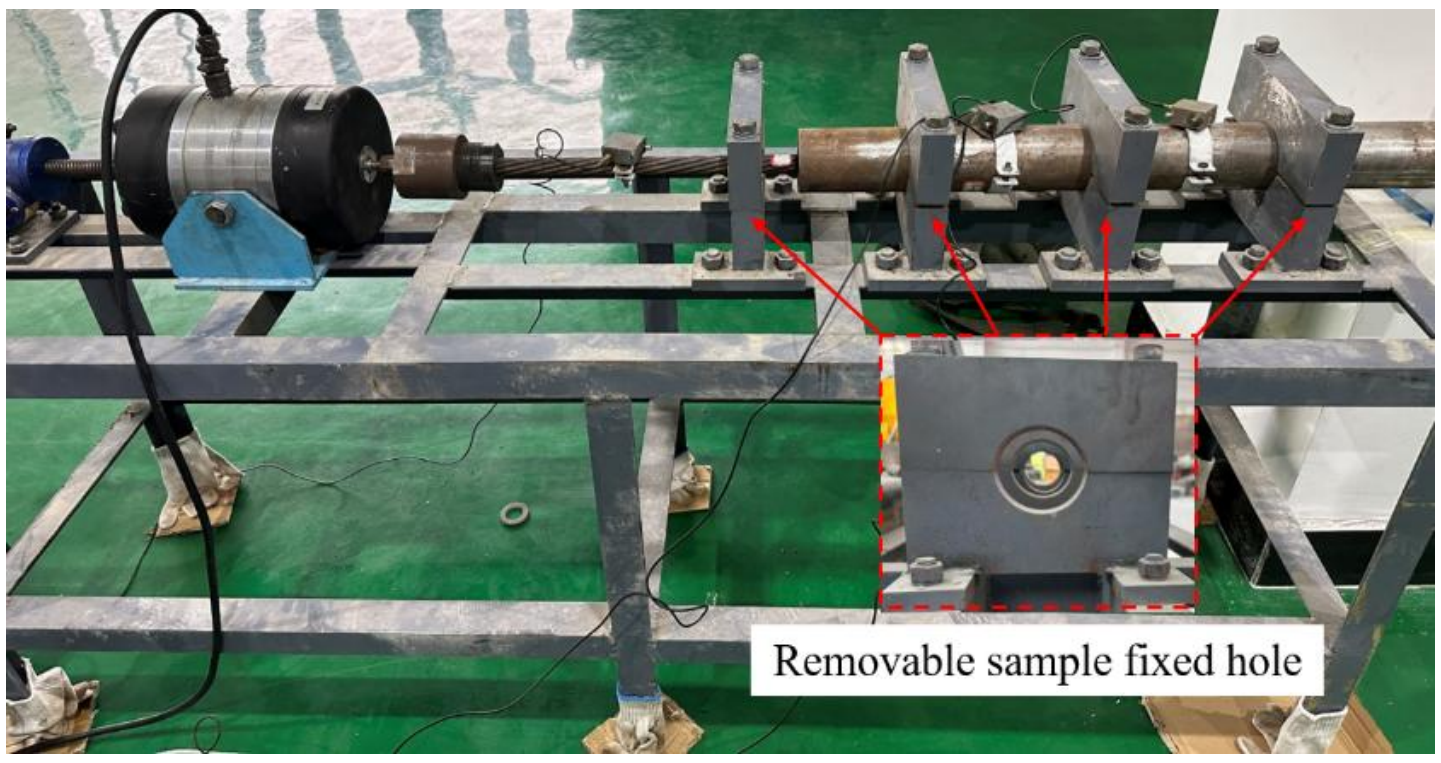

Fig 3. Anchor cable anchorage and external excitation test bench

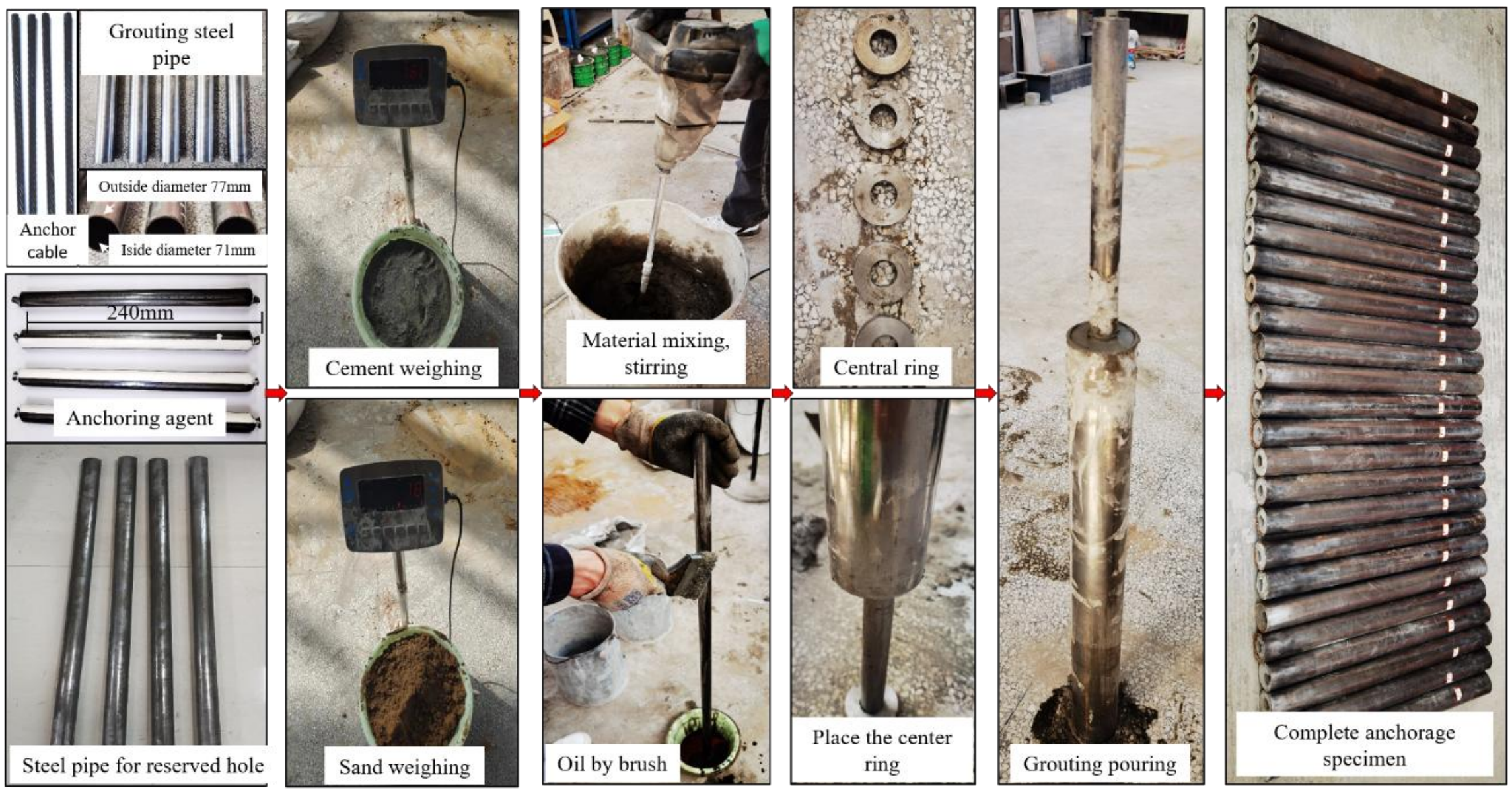

Fig 4. Preparation of test materials and anchorage matrix

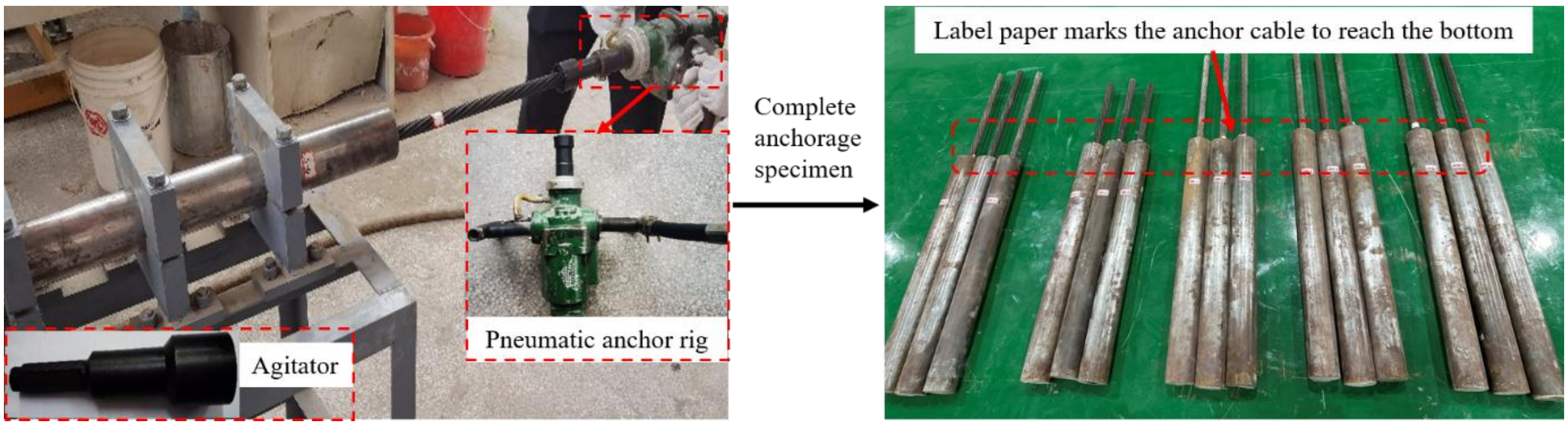

Fig 5. Anchoring specimen and complete specimen

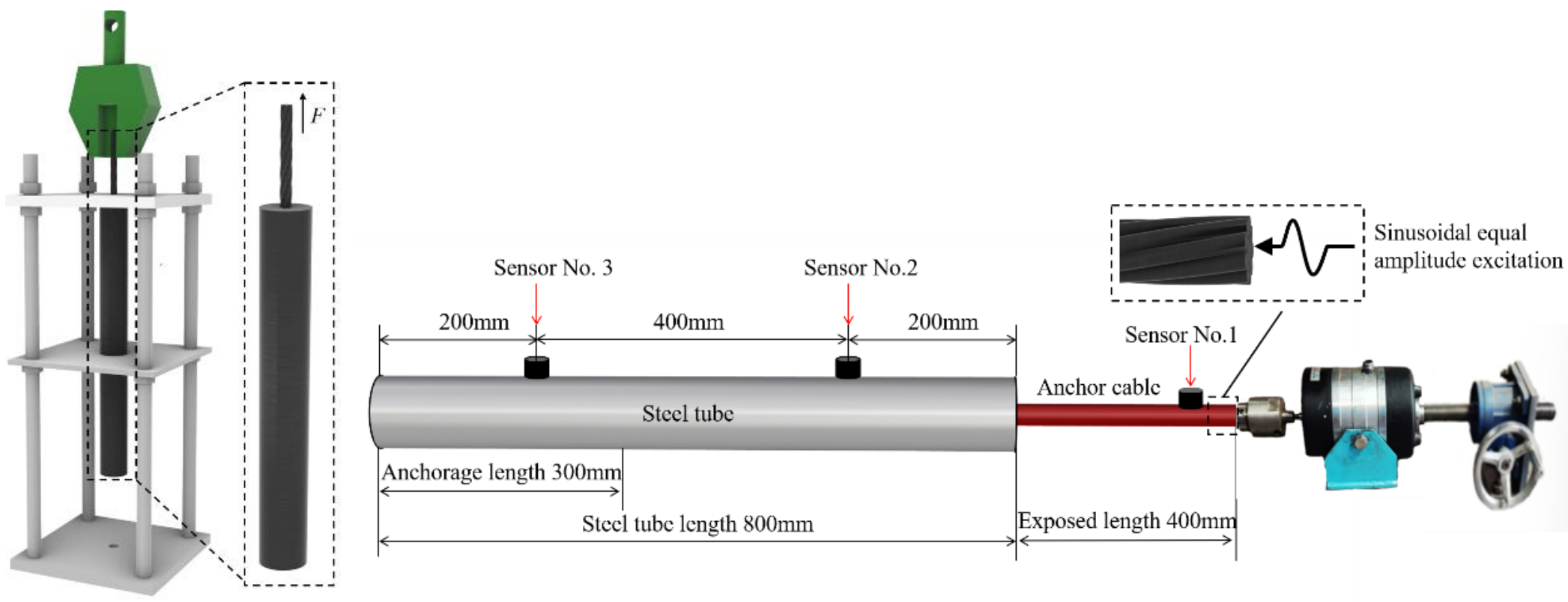

(a) Static load anchorage pull-out **(b)**External load excitation after vibration

Fig 6. Schematic diagram of the acceleration sensor and load application

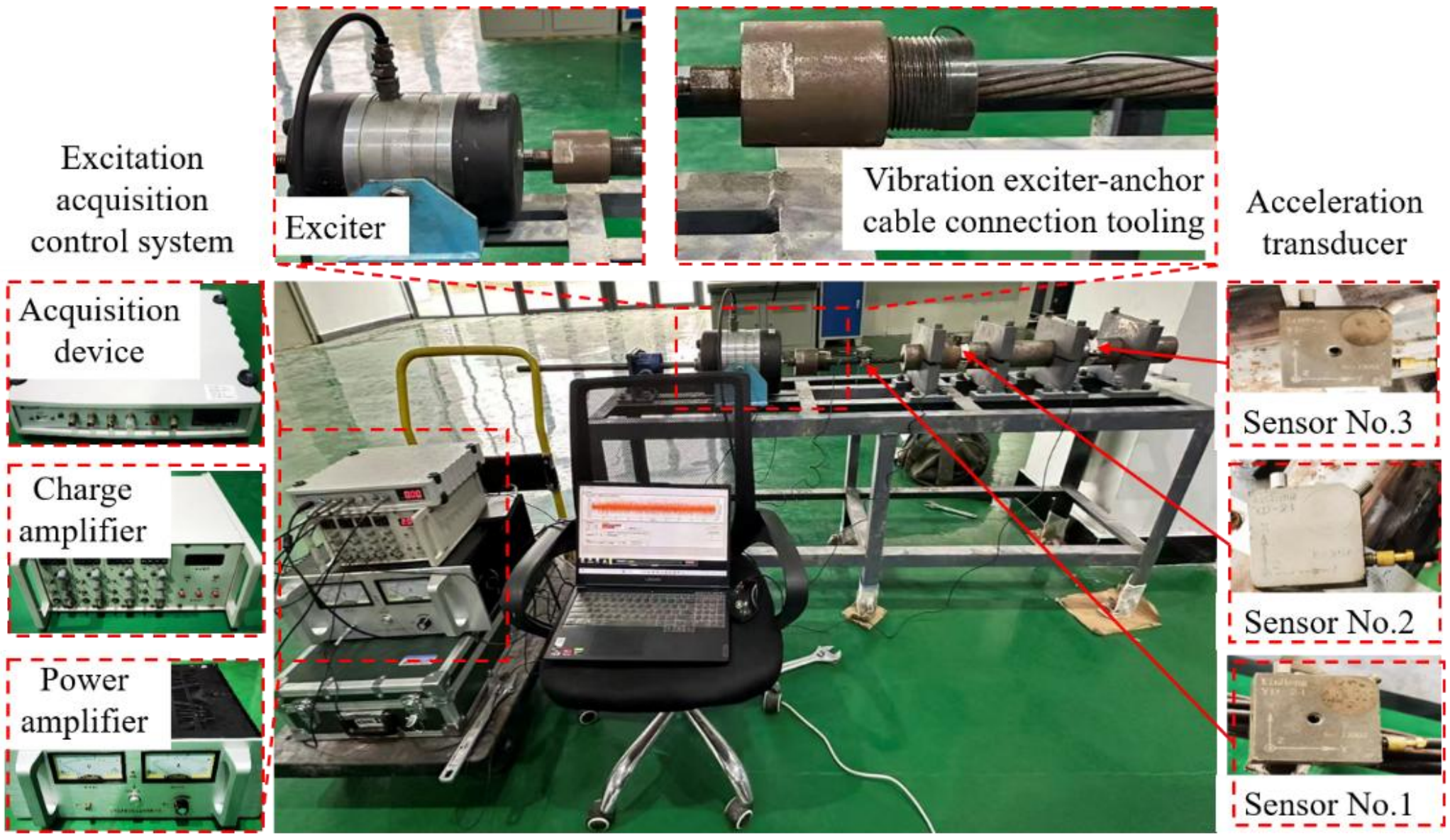

Fig 7. External sinusoidal load excitation test system of anchor cable anchorage specimen

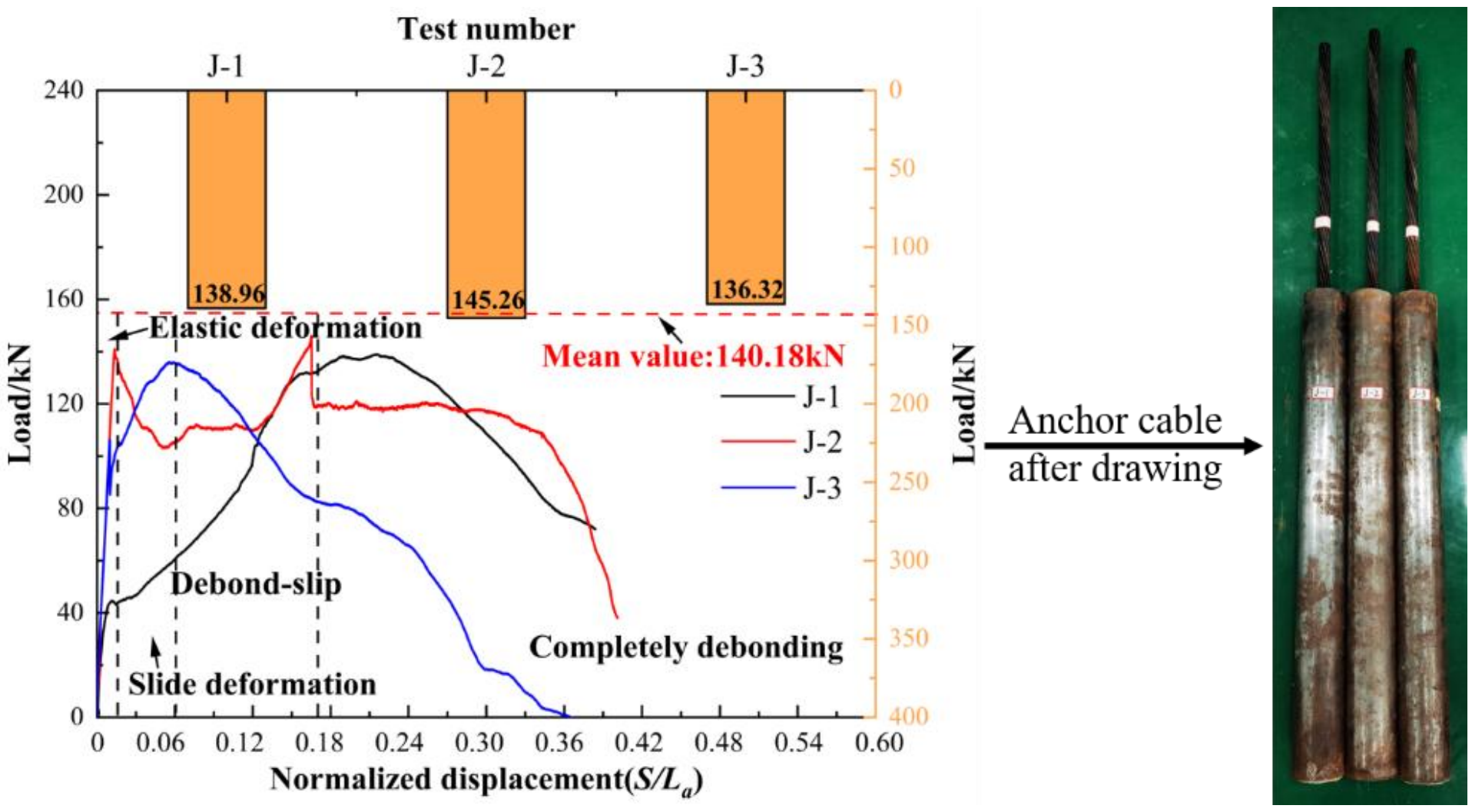

Fig 8. Normalized load-displacement curves under static load and post-test displacement of end-anchored cables

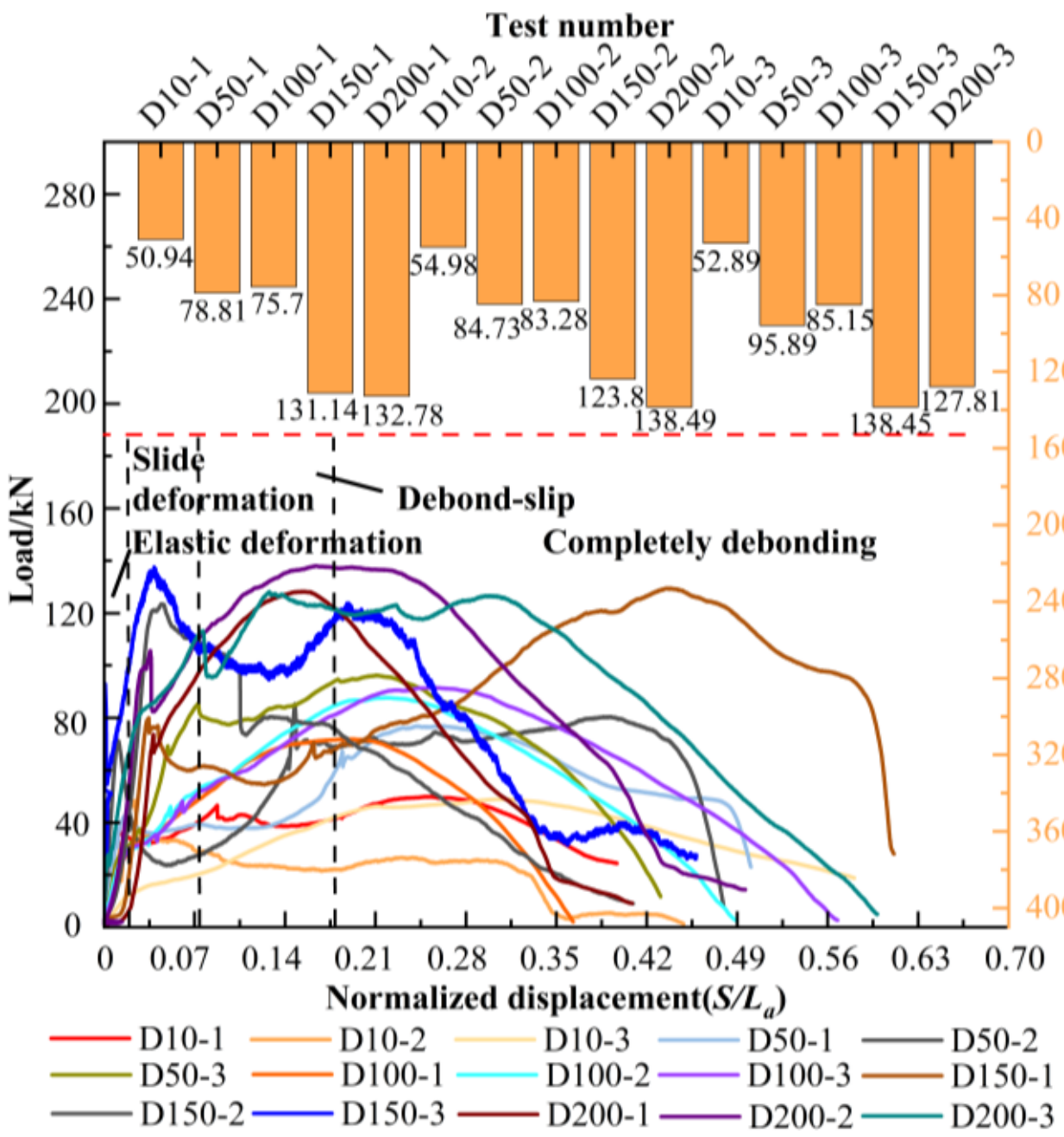

Fig 9. Normalized load-displacement curves of anchorage pull-out after sinusoidal load excitation at different disturbance frequencies

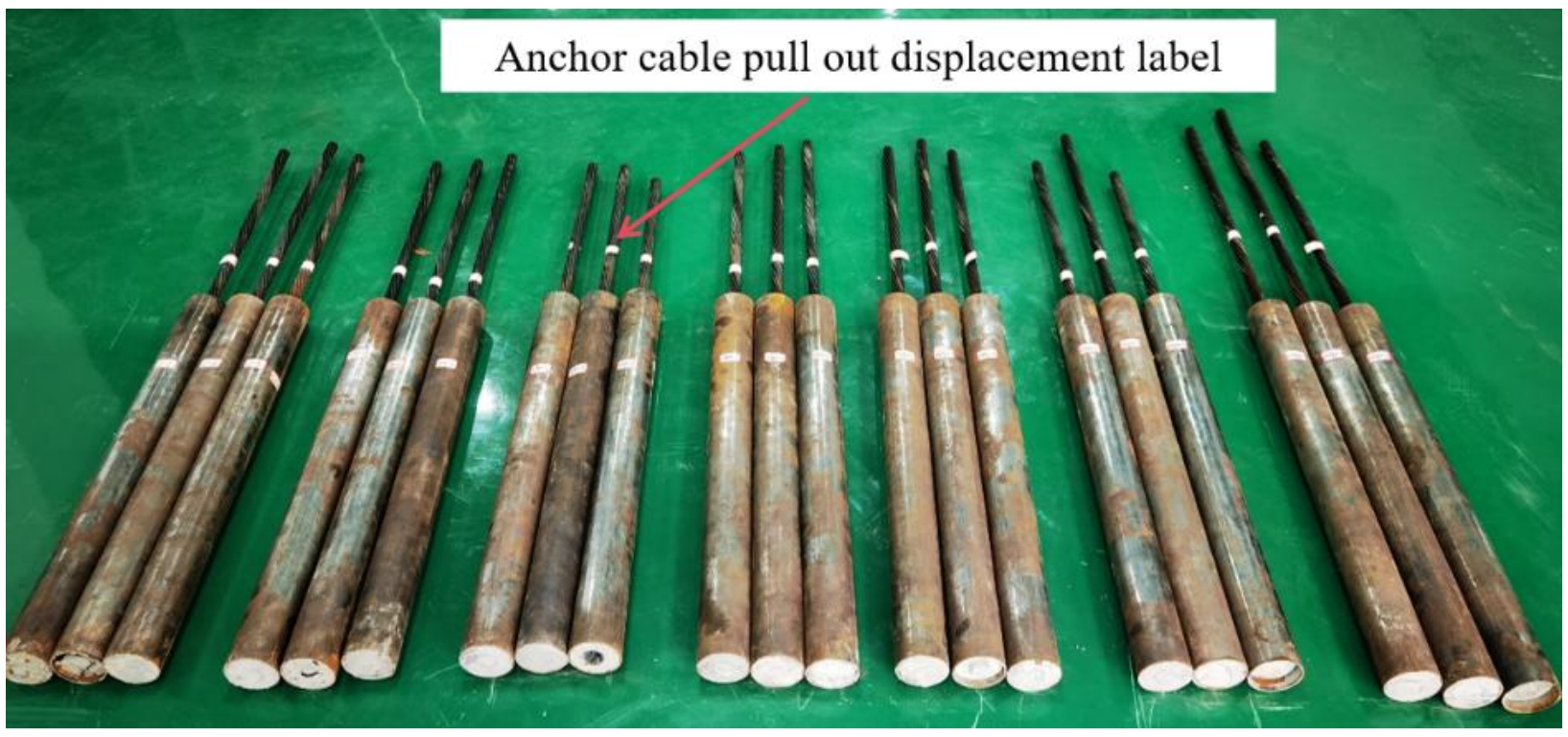

Fig 10. Displacement of anchor cables and steel pipe deformation after external sinusoidal excitation

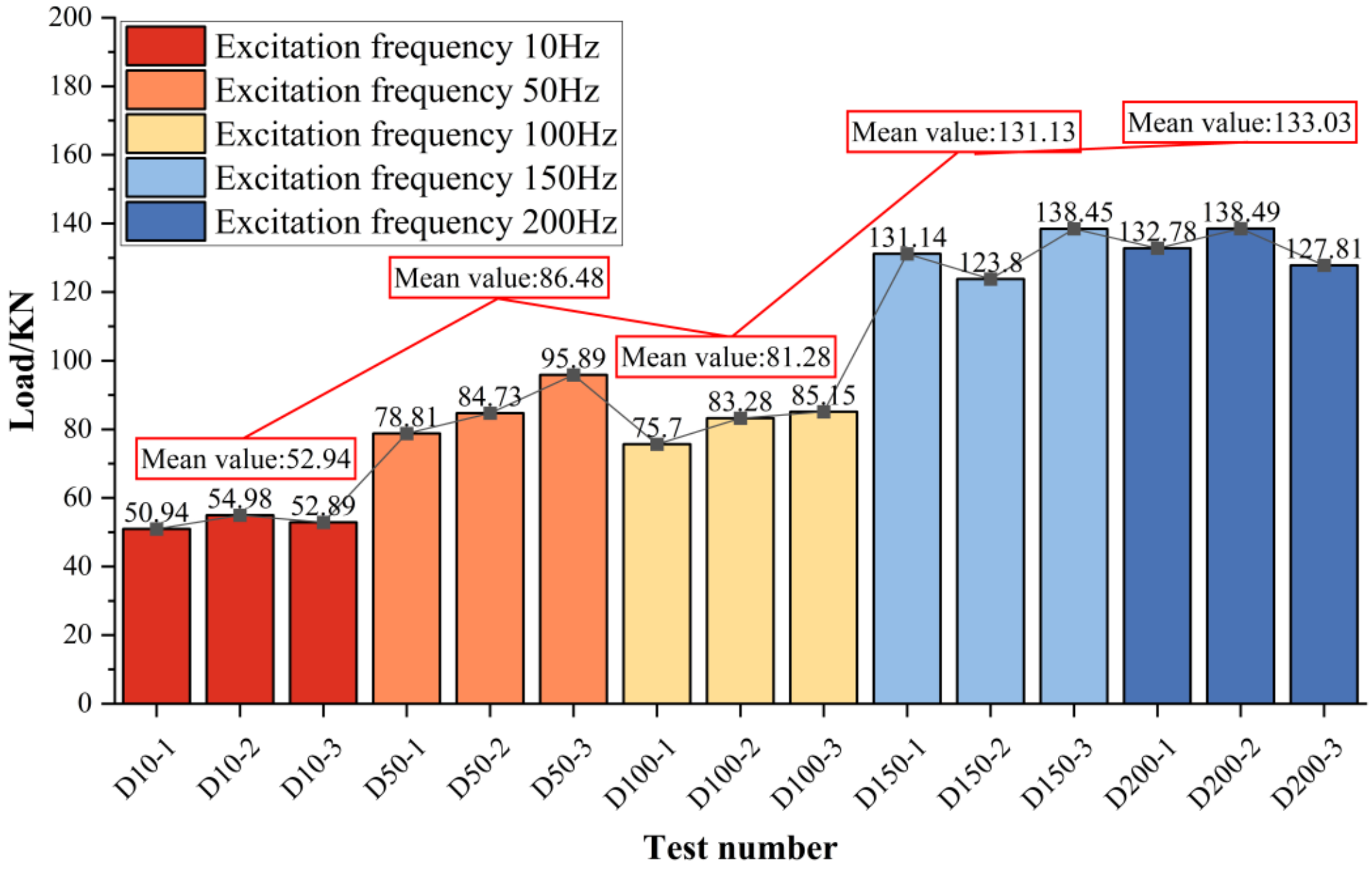

(a) Peak pull-out strength under different disturbance frequency excitation

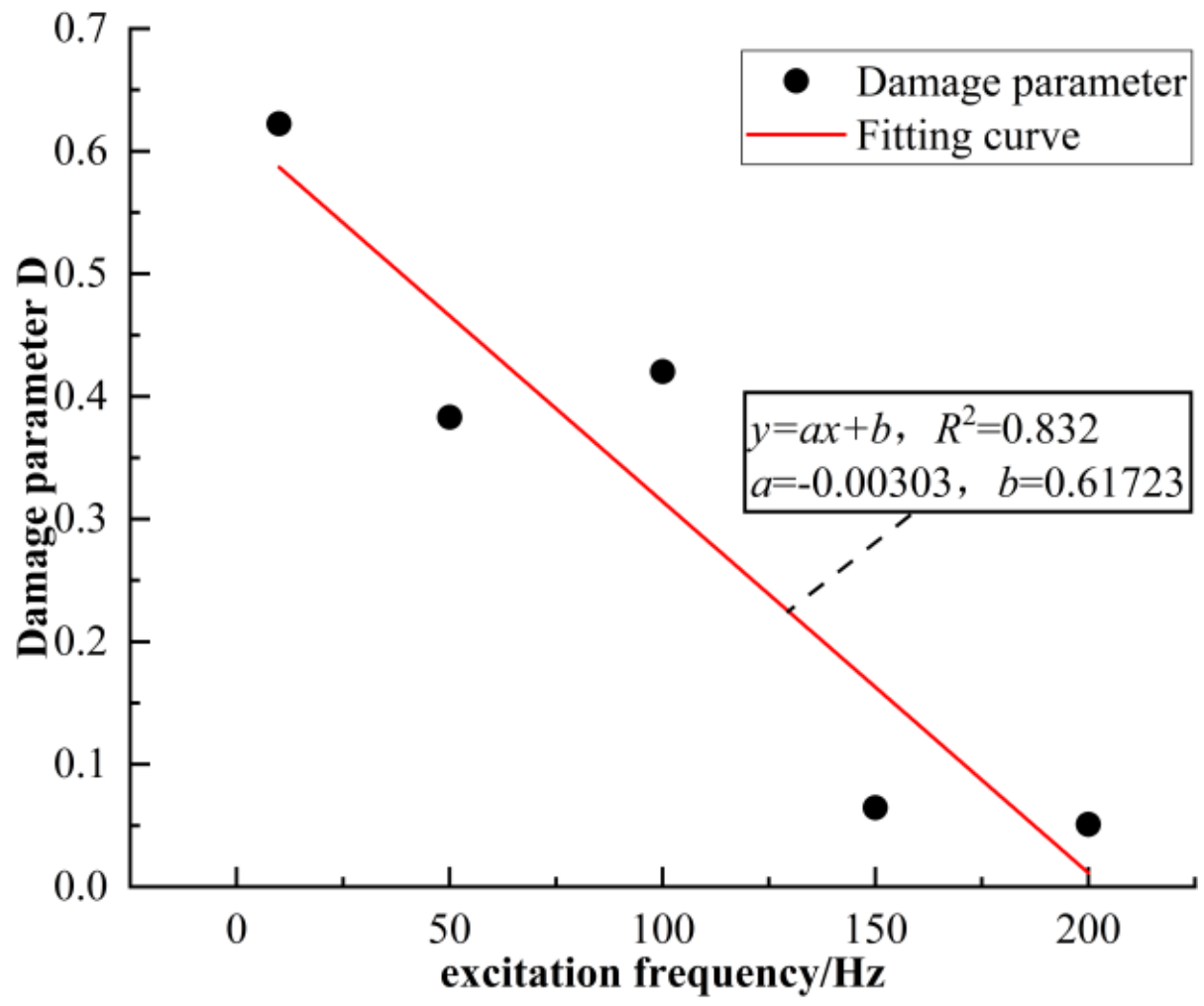

(b) Variation of damage parameters with excitation frequency

Fig 11. The change rule of damage degree of anchorage body after excitation

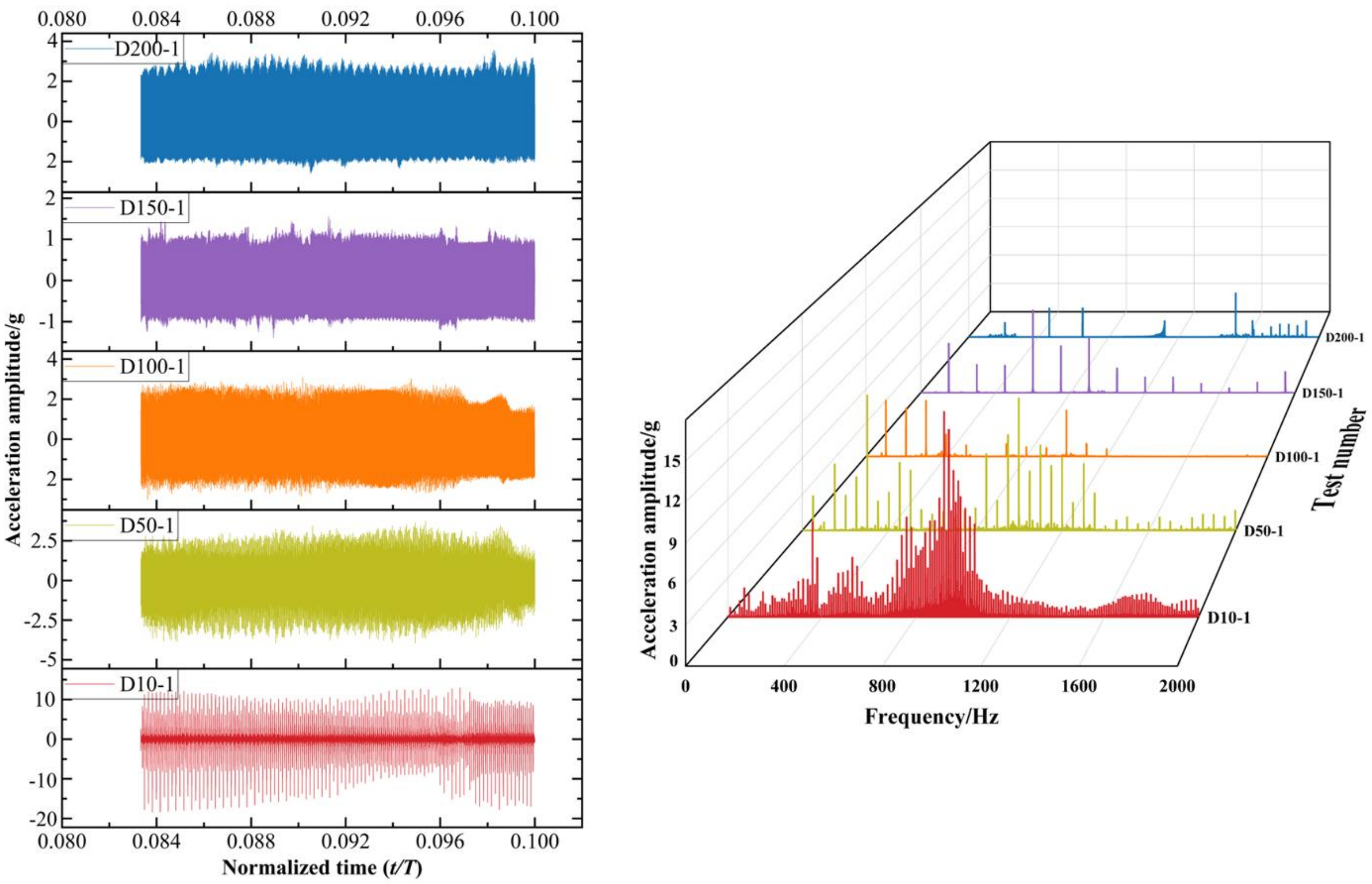

(a) Normalized time-domain waveform **(b)** Frequency-domain waveform

Fig 12. Normalized time-domain and frequency-domain acceleration response waveforms of anchor cable free segment under different disturbance frequencies

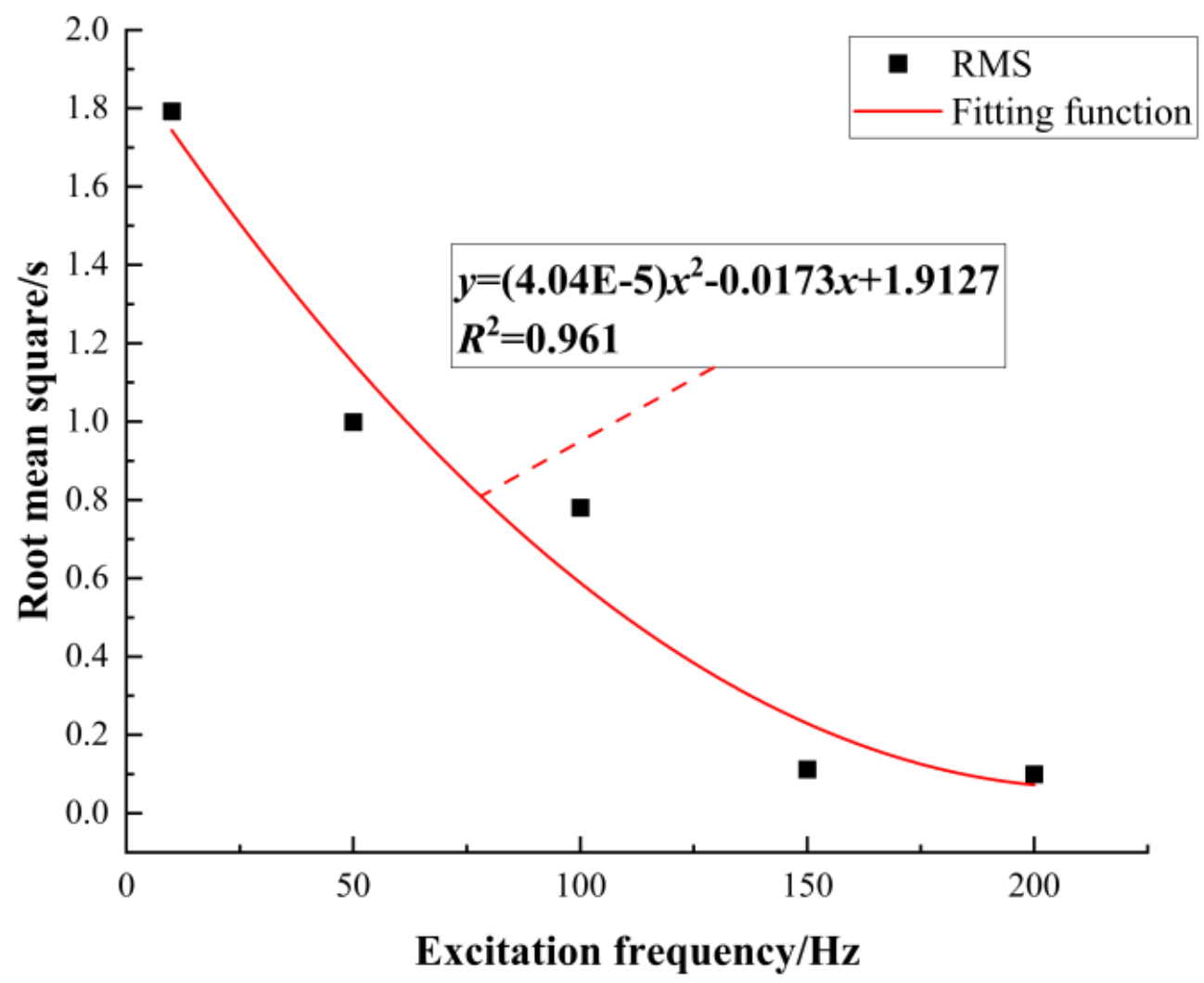

Fig 13. Fitting result of RMS and excitation frequency

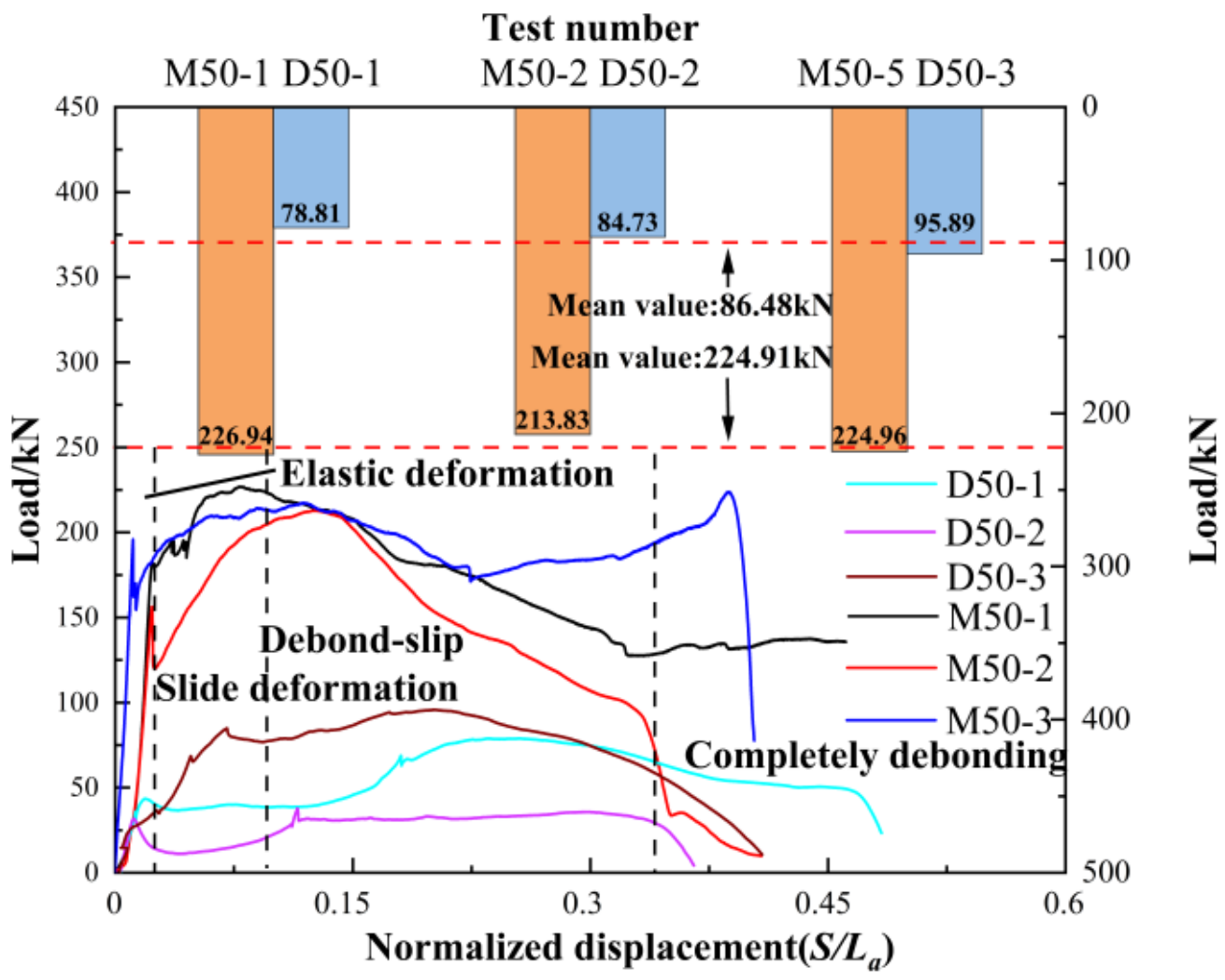

Fig 14. Comparison of normalized pull-out load-displacement curves of specimens with anchorage lengths of 300 mm and 600 mm

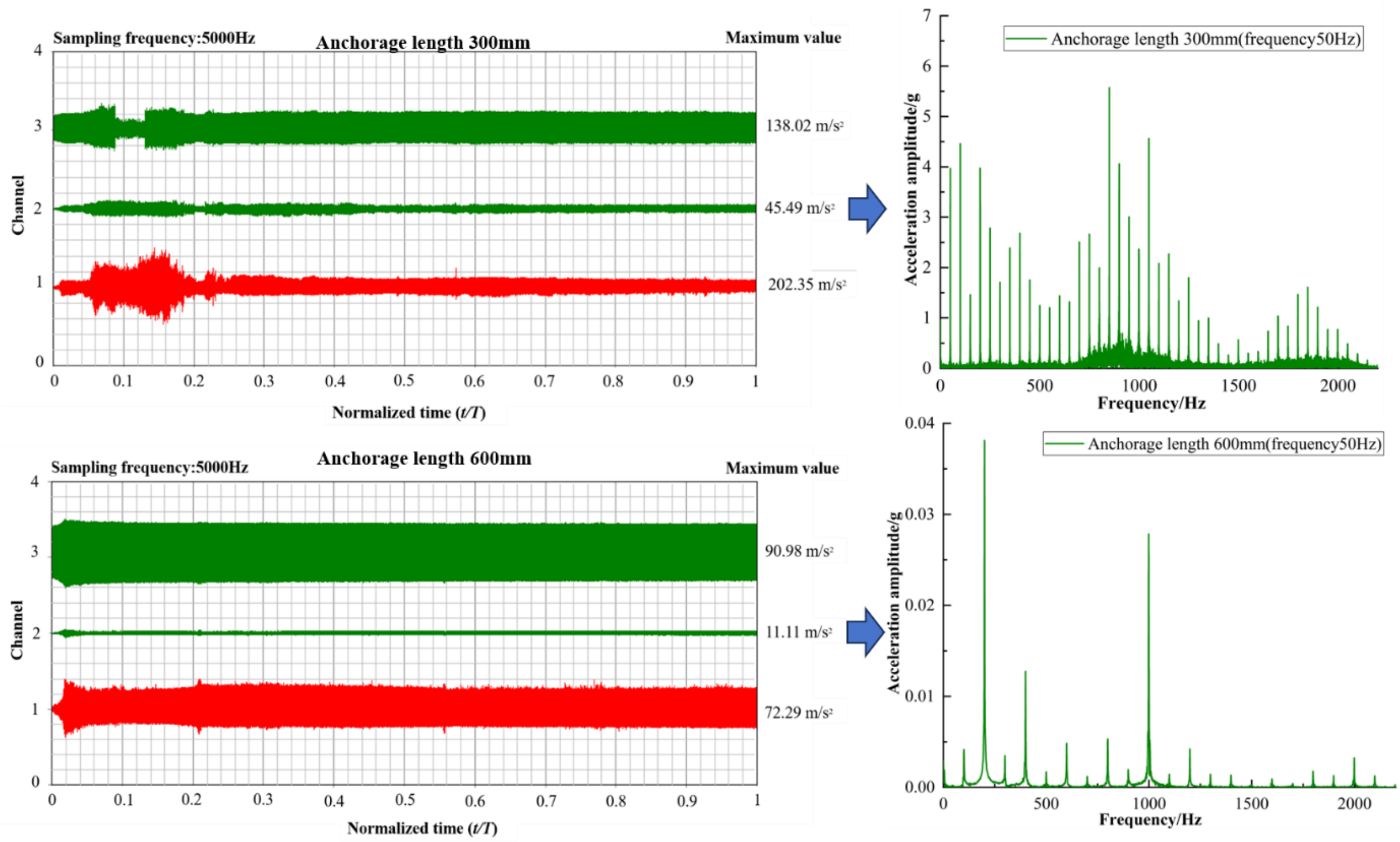

Fig 15. Comparison of normalized time-domain and frequency-domain waveforms of specimens with 300 mm and 600 mm anchorage lengths

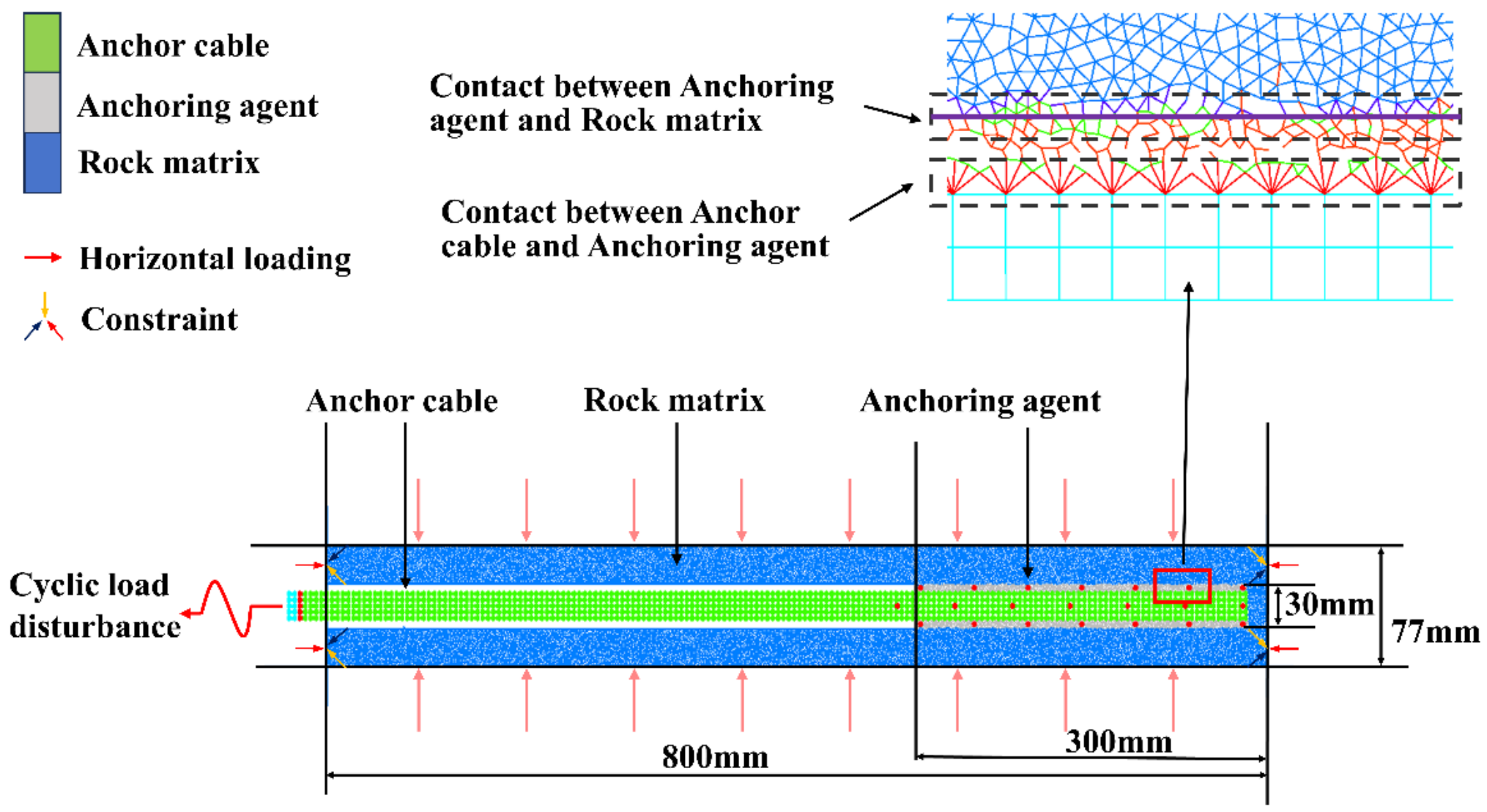

Fig 16. The establishment of a numerical model

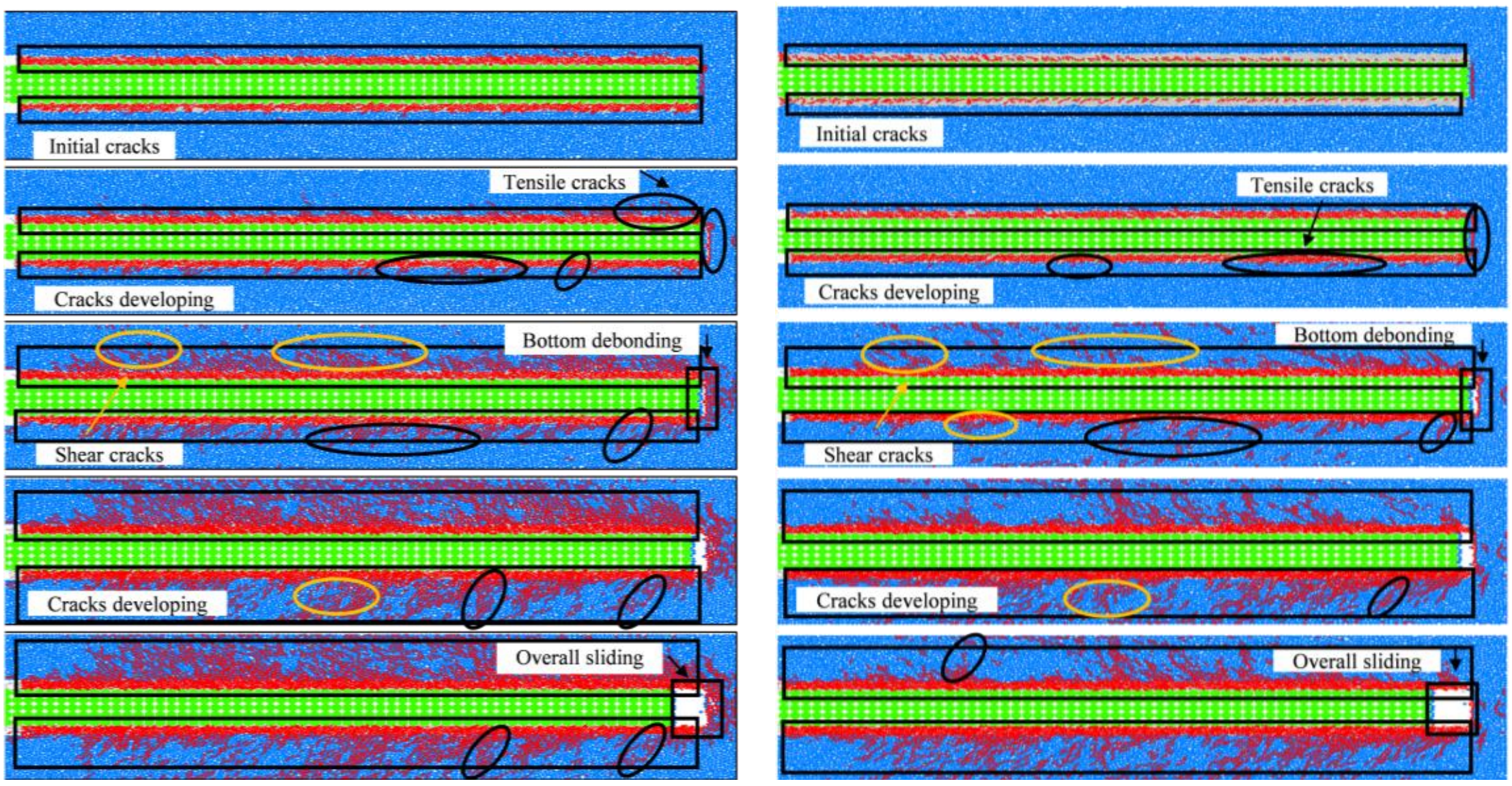

(a) Crack field evolution of anchorage body at 10 Hz **(b)** Crack field evolution of anchorage body at 50 Hz

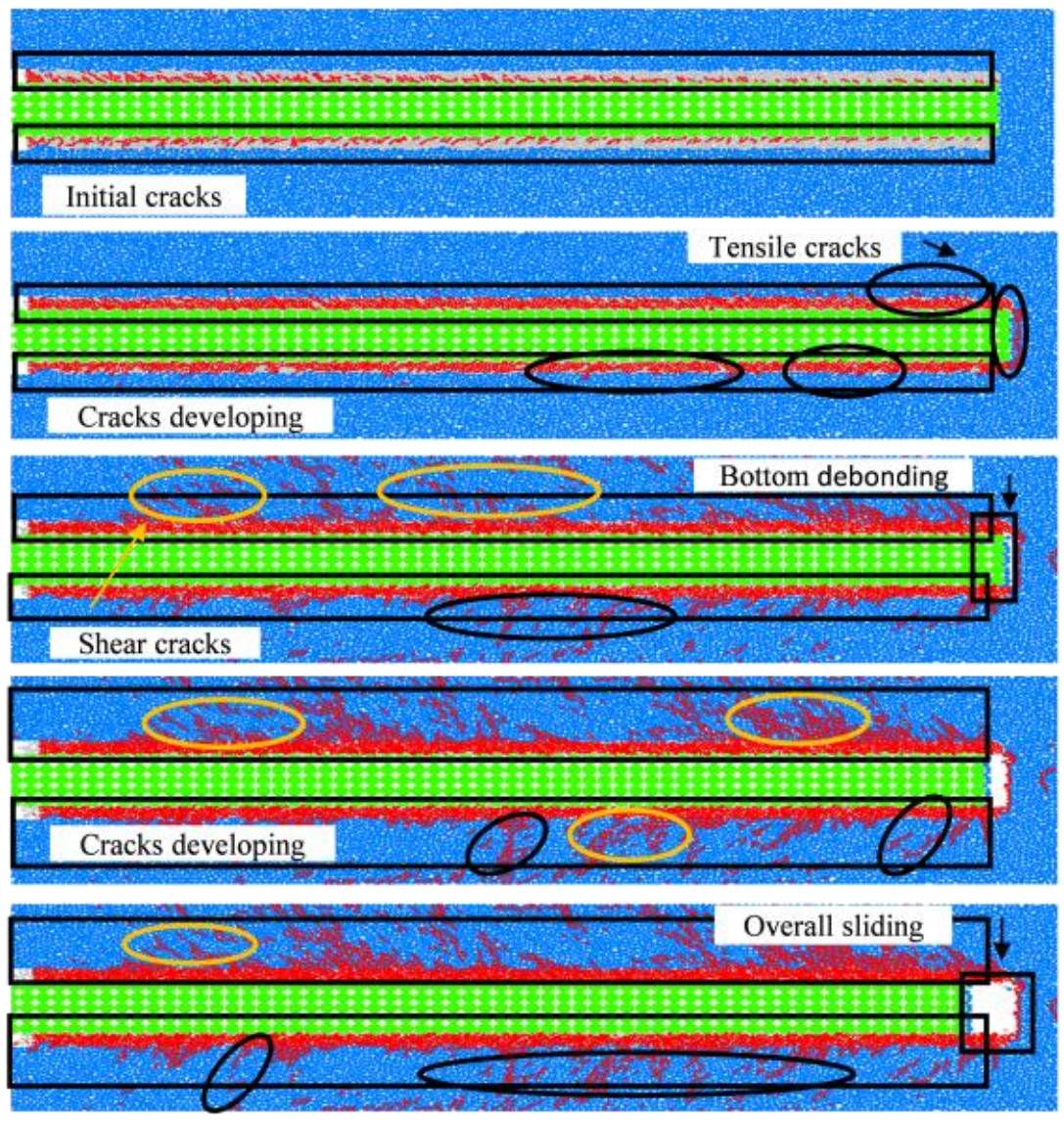

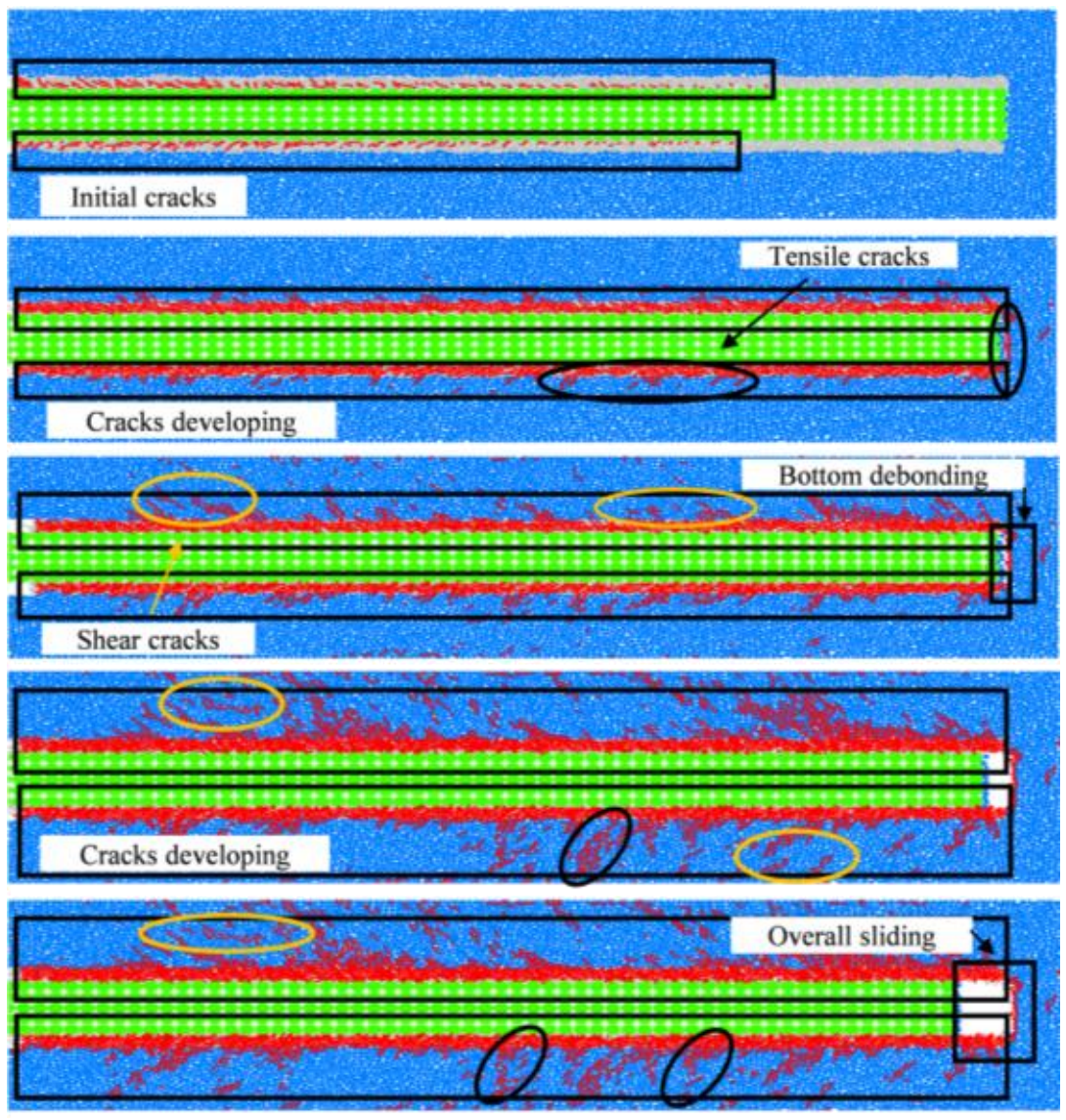

(c) Crack field evolution of anchorage body at 100 Hz **(d)** Crack field evolution of anchorage body at 150 Hz

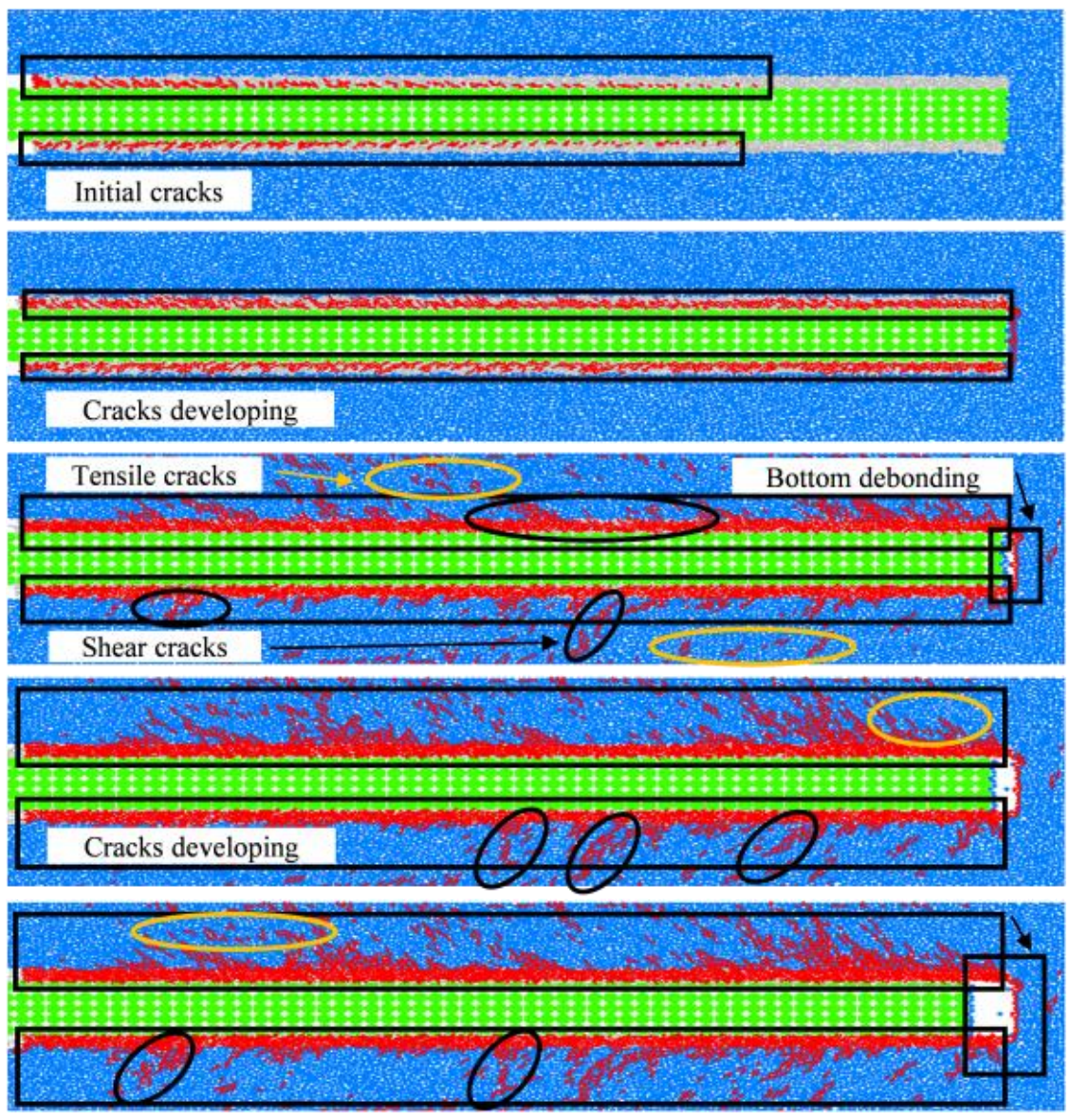

(e) Crack field evolution of anchorage body at 200 Hz

Fig 17. Evolution characteristics of internal fracture field in anchorage body under different disturbance frequencies

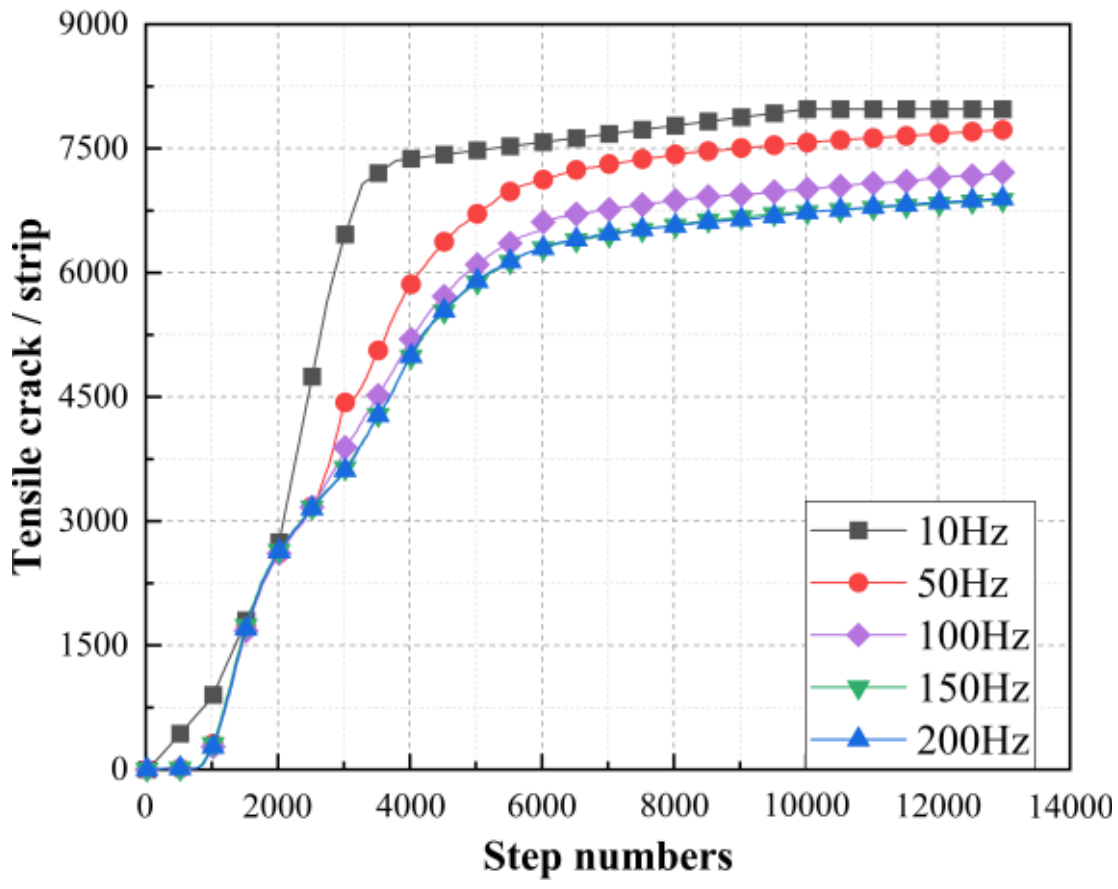

(a) Tension crack

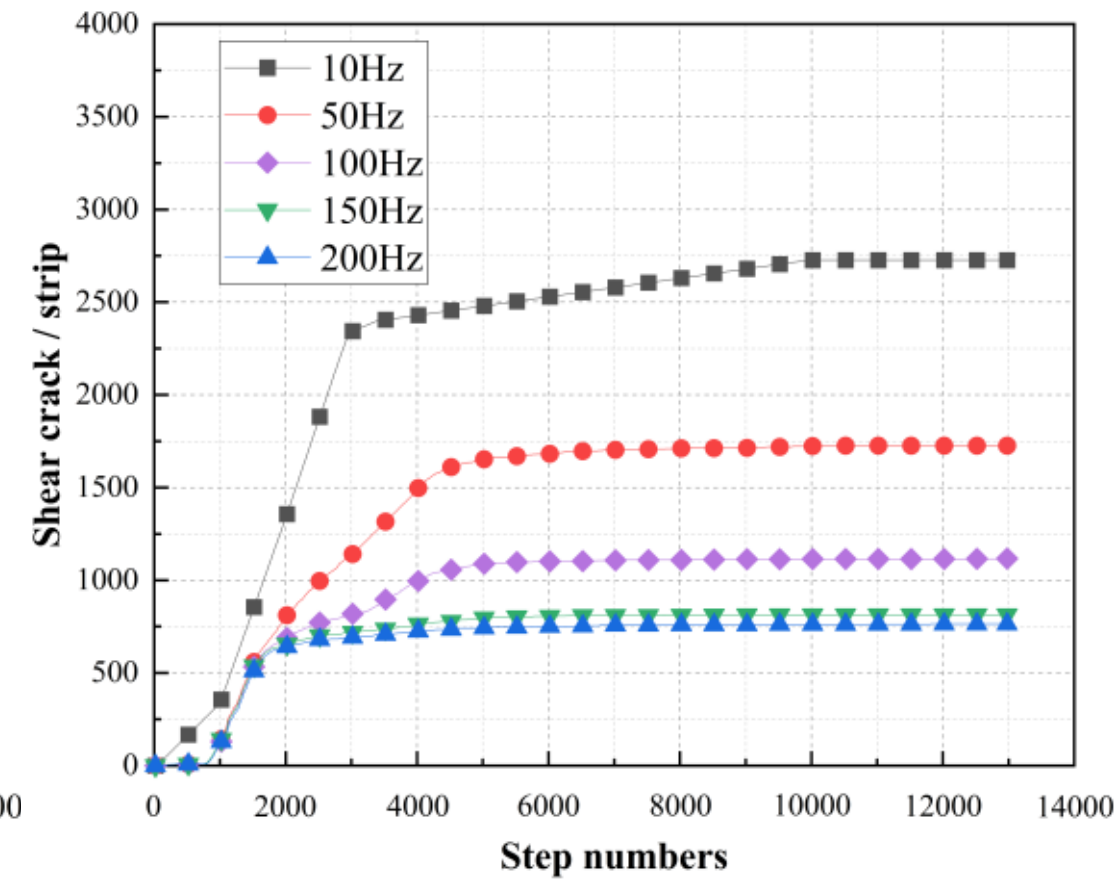

(b) Shear crack

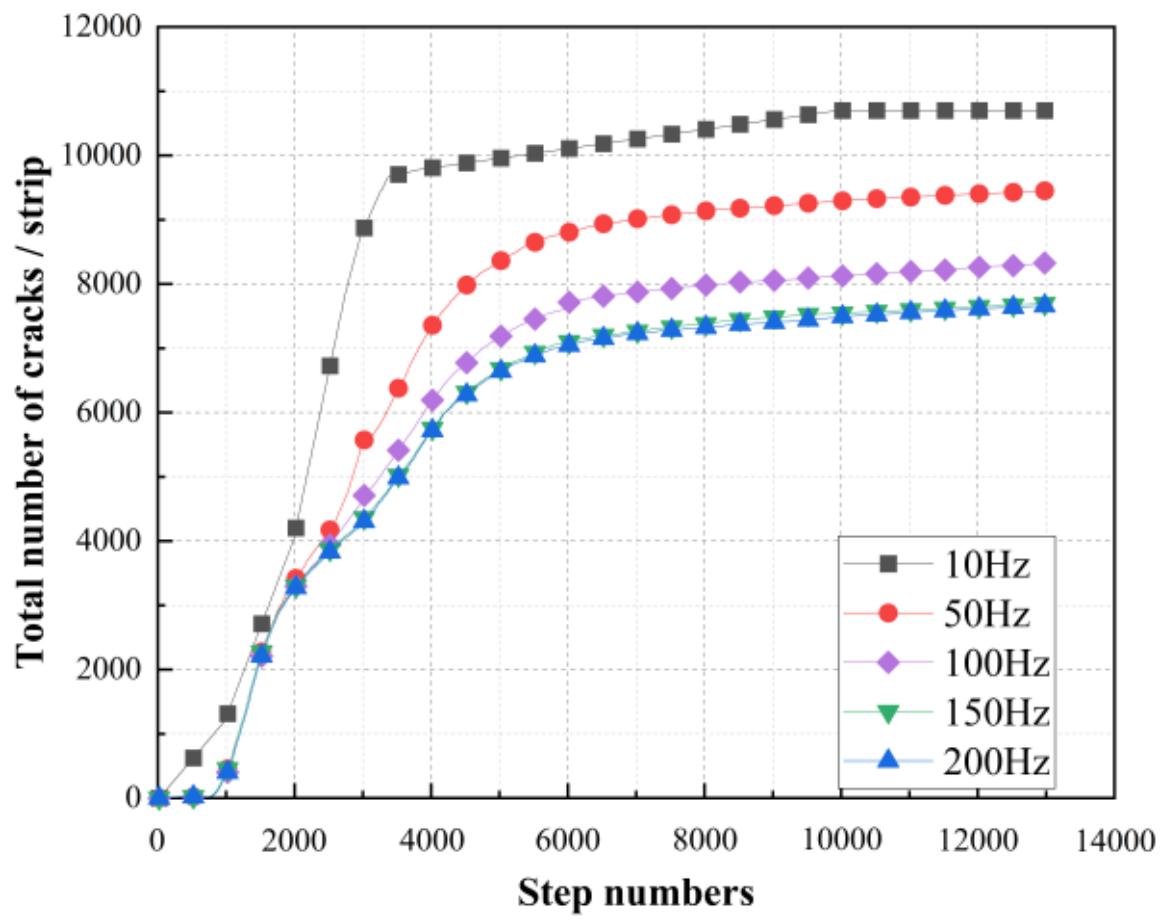

(c) Total number of cracks

Fig 18. Evolution curves of internal cracks in anchorage body under different disturbance frequencies

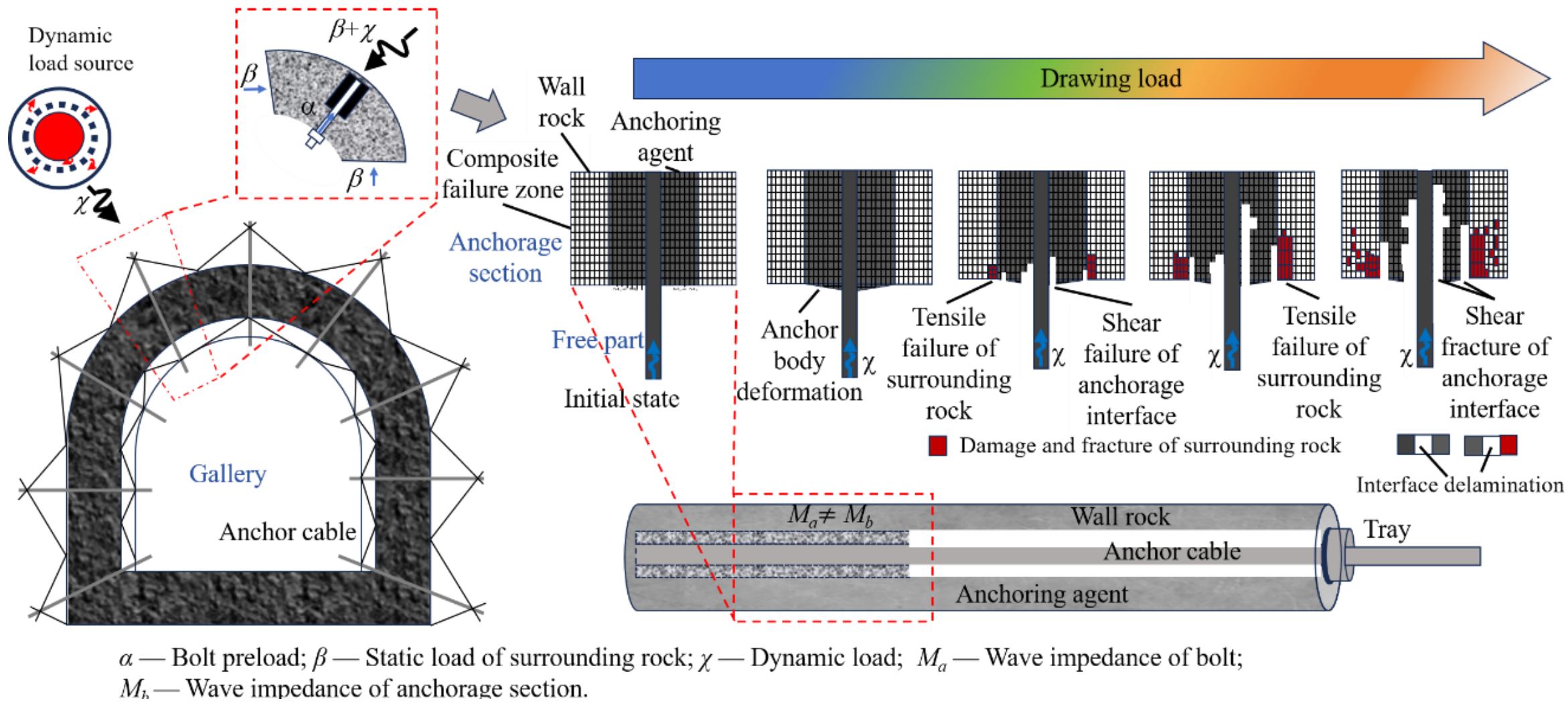

Fig 19. Progressive failure process of anchorage body under pull-out load

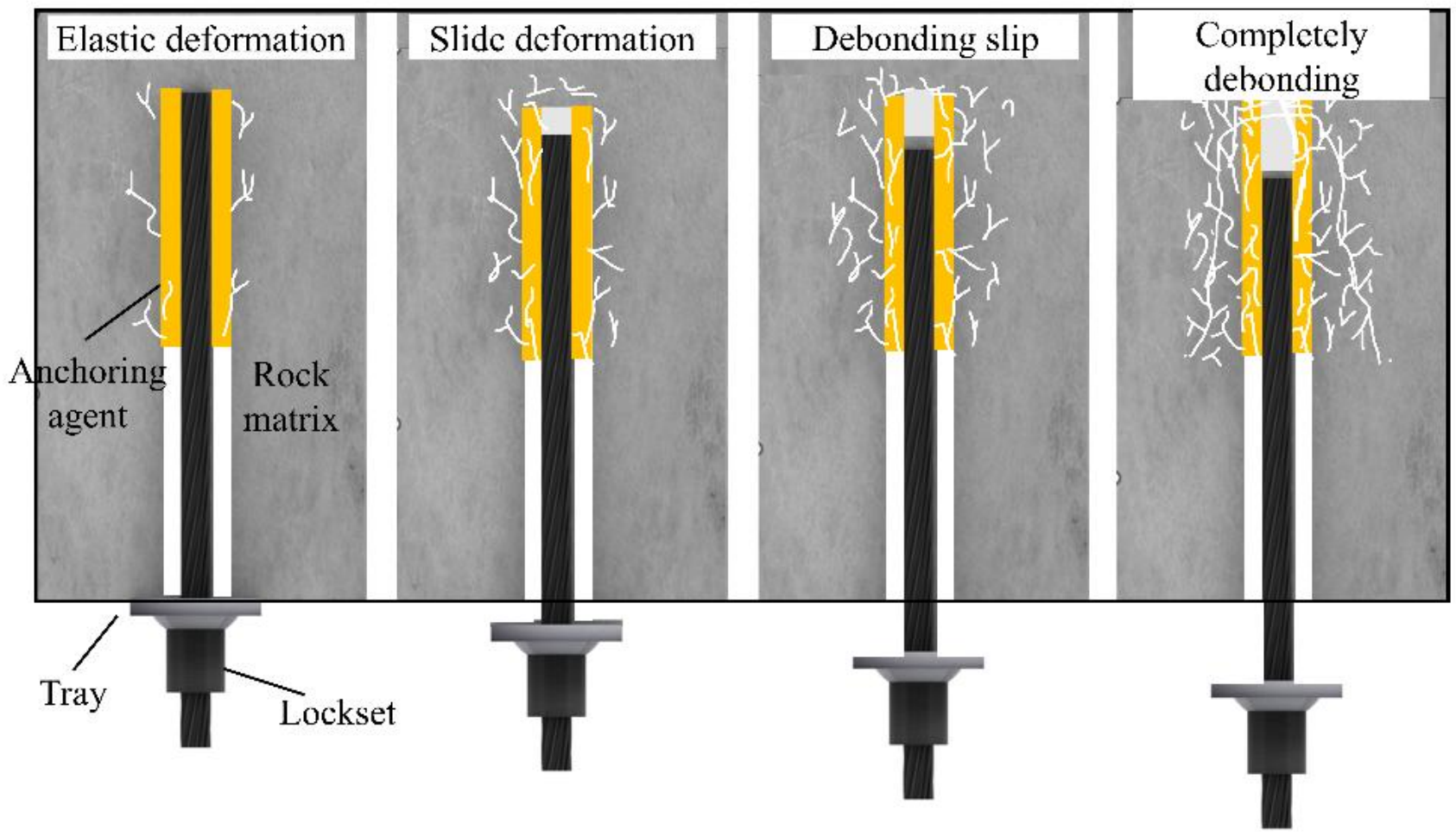

(a) Crack evolution under low-frequency disturbance

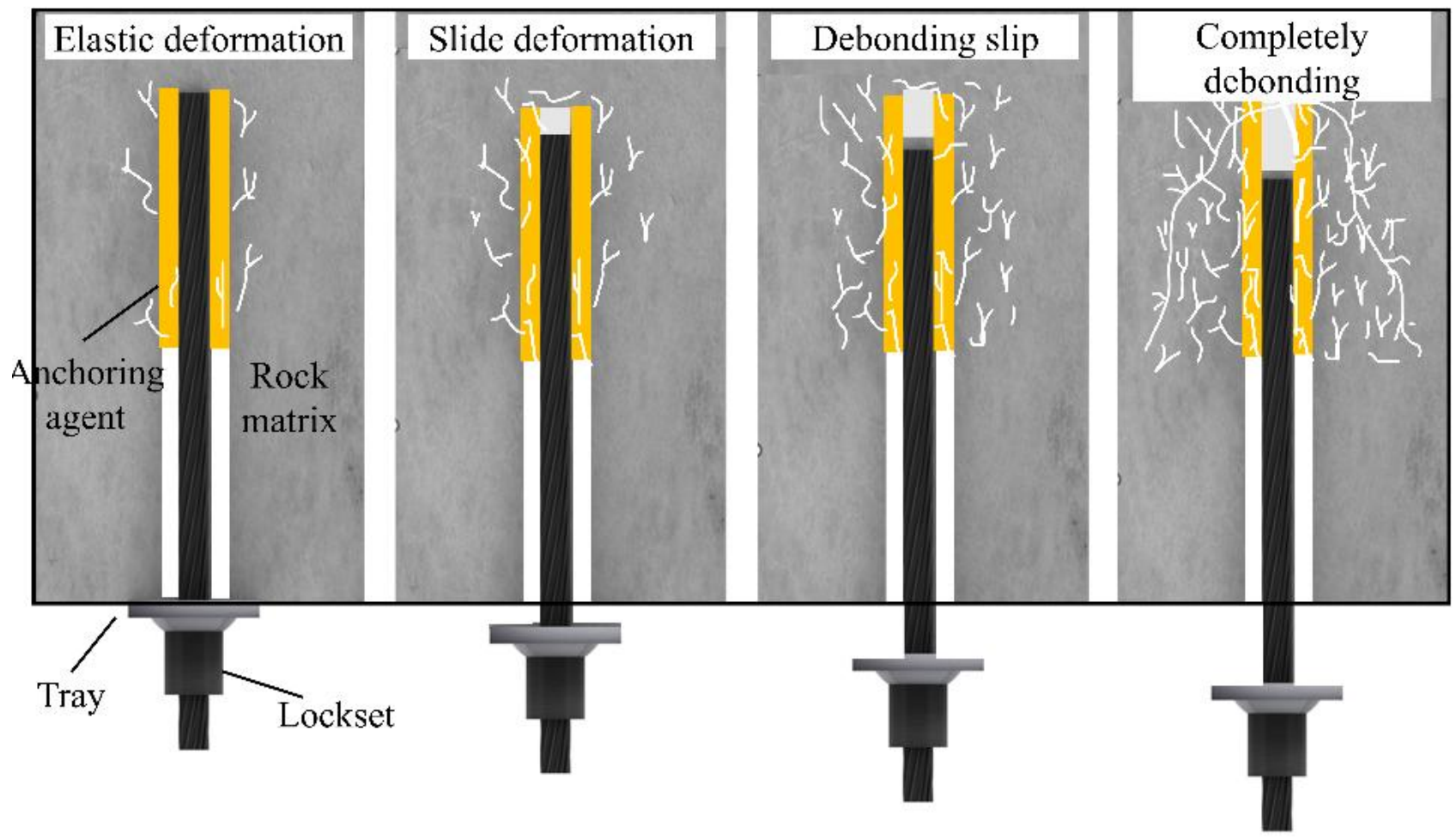

(b) Crack evolution under medium-high frequency disturbance

Fig 20. Schematic of crack evolution in anchorage bodies under different disturbance frequencies
